# The German Basic Psychological Needs Satisfaction in Physical Education Scale: Adaption and Multilevel Validation in a Sample of Sixth-Grade Girls

**DOI:** 10.3390/ijerph17051554

**Published:** 2020-02-28

**Authors:** David J. Sturm, Joachim Bachner, Stephan Haug, Yolanda Demetriou

**Affiliations:** 1 Department of Sport and Health Sciences, Professorship of Educational Science in Sport and Health, Technical University of Munich, 80992 Munich, Germany; joachim.bachner@tum.de (J.B.); yolanda.demetriou@tum.de (Y.D.); 2 Department of Mathematics, Chair of Mathematical Statistics, Technical University of Munich, 85748 Munich, Germany; haug@tum.de

**Keywords:** self-determination theory, basic psychological need satisfaction, questionnaire, multilevel validation, physical activity, questionnaire

## Abstract

(1) Background: Self-determination theory (SDT) claims that need supportive behavior is related to the satisfaction of the basic psychological needs: autonomy, relatedness and competence. The student–teacher relationship is of special interest to understand mechanisms of physical activity behavior change in physical education (PE). (2) Methods: In this cross-sectional study, 481 girls answered a German version of the Basic Psychological Need Satisfaction (BPNS) in PE Scale. Contrary to previous studies, the psychometric properties of this scale were examined by multilevel confirmatory factor analysis. (3) Results: A model with three latent factors on both levels showed acceptable fit and all items showed significant factor loadings. Although one item was excluded due to psychometric reasons, the scale showed good internal consistencies; α = 0.85 at the individual level and α = 0.84 at the class level. Subscales’ internal consistency at the individual levels was good, while at class level, the scores differed from poor to good. Small significant correlations of BPNS with moderate to vigorous physical activity support criterion validity. (4) Conclusion: The 11-item scale is a valid measurement tool to assess BPNS in PE and further application in the school setting would broaden the insights into the psychological impacts of SDT in PE.

## 1. Introduction

Numerous studies have pointed out the insufficient physical activity (PA) levels of children and adolescents in industrialized countries [1,2,3,4]. In light of the long-term consequences of physical inactivity, the World Health Organization (WHO) [5] predicts a generation of people suffering from chronic diseases all of which often exacerbated by too little PA. In Germany, there is a marked difference between actual and target conditions of PA, which is significantly more distinct for girls with a low socioeconomic status (SES), especially in the age group between 14 and 17 years [2]. Programs that promote children’s and adolescents’ PA are needed from an early age on [6]. The school setting provides the opportunity to implement appropriate programs [7], since every child and adolescent, independent of age and SES, is necessarily involved in activities embedded in the curriculum. Physical education (PE) teachers are well suited to motivate and educate children in adopting an active and healthy lifestyle, but how this support works in practice is in need of clearer definition in the context of PE.

**Figure 1:**
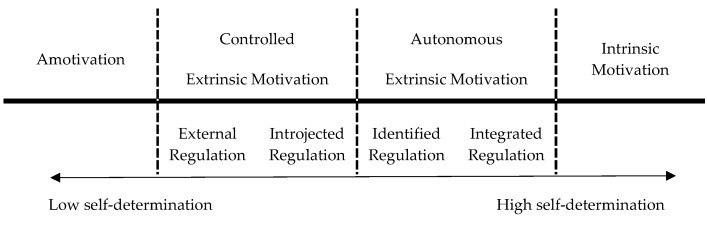
The self-determination continuum [10].

Clearly, we must also consider the environmental factors and individual circumstances which influence PA behavior [8]. Initially, the environment must provide an opportunity and motivational processes must be triggered so that the individual engages in PA. A sub-theory of the self-determination theory (SDT) explains the types of motivation. The organismic integration theory differentiates the degree of behavior regulation on a self-determination continuum, ranging from amotivation to intrinsic motivation (see Figure 1) [9]. Total lack of motivation characterizes amotivation. At the level of external regulation, the individual acts completely heteronomously, influenced by external interventions, such as reward and punishment. In this sense, an individual shows controlled extrinsic motivation, which includes also introjected regulation. Characterized by a successive increase of autonomy, the identified and integrated regulation accounts for autonomous forms of extrinsic motivation. Ultimately, intrinsic motivation is characterized by the most autonomous behavior activated by individual volition, personal interest or an exciting challenge [9].

According to SDT, every individual has the natural, constructive tendency to interact with other individuals in their environment, to act effectively in this milieu and experience themselves as proactive and autonomous [9]. The three basic psychological needs (BPN) autonomy, competence and relatedness derive therefrom. Philosophical definitions of autonomy do not necessarily equate it with an individual’s independence from their (social) environment. The preferred definition of autonomy is based on Ryan and Deci [9], i.e., if a person conforms to the stimuli and norms of their environment, they will likely adjust their own behavior to it voluntarily. Relatedness consists in the desire to feel connected with others, integrated and accepted as a member of the community. This belongingness is characterized by the recognition and positive value accorded to the related social environment [9]. The need for competence is defined as the subjective certainty that one can meet challenges in different situations based on one’s own competence [9].

In the context of PE, researchers have proven the positive correlations between support and satisfaction of BPN [11,12], which was recently underlined by a meta-analysis by Vasconcellos et al. [10]. BPN satisfaction (BPNS) in turn leads to autonomous forms of motivation [13]. For adolescents in particular, these types of motivation have been shown to be determinant in promoting PA behavior [14,15]. A closer look into the relations of BPNS and PA-related constructs reveals unambiguous results. In their systematic review, Teixeira et al. [16] claim that competence satisfaction in the school setting predicts more exercise participation across all ages. An experiment in PE context by Hagger et al. evinced the direct influence of perceived autonomy support towards leisure time PA [17]. Moreover, perceptions of relatedness, autonomy and competence serve as motivational predictor towards PA for adolescents [18].

In order to evaluate these theoretical considerations and to establish the SDT in a domain-specific sample, instruments to assess the BPNS are mandatory. Based on the original Basic Psychological Need Satisfaction and Frustration Scale (BPNSFS), several scales were developed by adapting them to specific criteria of domain, language and age. A confirmatory factor analysis (CFA) of the original 24 items by Chen et al. exhibited a good fit for a hypothesized 6-factor model, assuming separate dimensions for satisfaction and frustration for each of the three needs [19]. Internal consistencies of the satisfaction subscales of the three needs were at α = 0.81 and above. Haerens et al. validated the scale in a sample of Dutch-speaking students in the context of PE and established the hypothesized three-factor structure on both dimensions, showing good internal consistencies of α = 0.87 for the satisfaction subscale [20].

Given our focus on the satisfaction of the three innate psychological needs, the entire BPNSFS was not appropriate for the incorporation in a comprehensive questionnaire. Furthermore, negative need fulfillment has been pointed out as a distinct dimension, which justifies in light of adverse health outcomes a separate investigation [19,20]. Besides, two scales assessing the BPNS are relevant in the sports context. The Psychological Need Satisfaction in Exercise Scale [21] and the Basic Psychological Need Satisfaction Scale in Exercise [22], of which the latter one was validated and adapted for the PE context in Greek [23]. Containing 12 items, the BPNS-PE supported a three-factor structure and had high internal consistencies, which was recently confirmed by CFA in a Spanish [24] and English sample in PE [25]. Trigueros et al. examined an adequate fit for a four-factor solution of the Spanish Scale of the Satisfaction of Psychological Needs in Physical Education [26], including a fourth subscale to assess the newly introduced need of novelty [27], and estimated an acceptable reliability by Cronbach’s alpha of α > 0.70.

One limitation of previous validations derives by using CFA for data assessed in the school setting, since students are clustered in classes. Ignoring the clustered nature of data could lead to biased estimates and misinterpretations, since already small intercorrelations have an impact on model estimates and variances [28]. Furthermore, Haerens et al. provided indications that the need satisfaction had a significant variance on the class level by a multilevel analysis of their intervention effects [20]. Consequently, it is recommended to account for the clustered data structure by using a multilevel CFA (MCFA) [29].

As educational policy is a matter of the respective federal states in Germany, the curriculum differs between states. In secondary schools in Bavaria, two PE lessons (each 45 min) per week are mandatory at class stages 5 to 10. A male or female PE teacher carries out the gender-separated PE lessons, respectively. Girls represent a specific risk group regarding the effects of age, gender and SES on PA [2]. Therefore, single-sex interventions are necessary in order to meet the needs and interests of girls.

To date, no measurement instrument exists that is rigorously validated to examine the BPNS of German-speaking adolescents within the PE context. Questionnaires are needed that are specifically designed for adolescents by addressing their stage of development and language. The purpose of the study is to provide initial evidence of reliability and validity of scores derived by the German Basic Psychological Need Satisfaction in Physical Education Scale (GBPNS-PE). According to Huang’s MCFA approach [30], the factor structure and scale dimensionality of the GBPNS-PE were examined as well as the criterion validity in relation to device-based assessed PA by basic multilevel analysis. Moreover, internal consistencies were estimated for the individual (within) and the group (between) level.

## 2. Materials and Methods

### 2.1. Participants

The sample derived from the single-sex intervention study CReActivity, which aimed to promote PA especially for girls [31]. We sampled 507 girls (aged 11.61 ± 0.55, range: 9 to 14) from 33 all-girl PE classes of the sixth grade from secondary schools (Realschule) in the area of Munich, Germany. Twenty-six students were excluded from the analysis due to missing values.

### 2.2. Measures

#### 2.2.1. Basic Psychological Needs Satisfaction

The BPNS in PE was assessed by an adapted and translated version of the BPNSFS by Chen et al. [19]. Two English-speaking researchers translated the 12 satisfaction items into German independently and discussed differences between the two versions. For the purpose of the present study, we adjusted the scale by adding the stem “Im Sportunterricht…” (“In PE lessons…”) at the top of the questionnaire and adapted towards age-appropriate style and language. In this case, the recommended back-translation technique was not beneficial. Therefore, the scale was pilot tested with the pretest procedure. Consisting of three subscales with four items each, the scale surveys the satisfaction of autonomy, competence and relatedness. Items were rated on a 5-point Likert scale representing the level of agreement with the statements from 1 (completely disagree) to 5 (completely agree).

#### 2.2.2. Physical Activity

**Figure 2:**
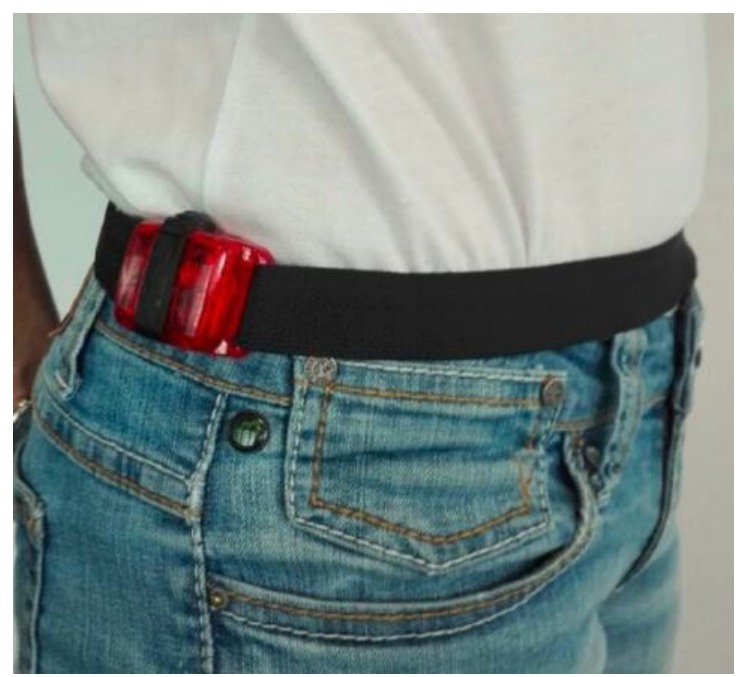
Students wore the ActiGraph GT3X-BT on the right hip attached with an elastic belt.

PA was assessed by accelerometry. Participants wore an ActiGraph GT3X or GT3X-BT (see Figure 2) for seven consecutive days except during water-based activities on their right hip. Participants had to put on the device after getting up, until nine pm or until they went to bed. Sampling rate was set to 30 Hz or 10 s for the older devices, respectively. ActiLife was used to initialize the devices and download the data [32]. A participant was included in the PA analysis if she achieved a valid wear time of at least 4 days with 8 hours wear time, of which one day was on the weekend. Data were observed during a period from five a.m. to nine p.m. Average duration of moderate-to-vigorous physical activities (MVPA) was analyzed using the cut points (≥3361 cpm) by Hänggi et al. [33]. Further details are described in Demetriou and Bachner’s work [34].

#### 2.2.3. Socio-Economic Status

Participants reported parents’ occupations and job description. A trained committee of four student research assistants coded the written answers according to the International Standard Classification of Occupation 2008 (ISCO-08). After revision by two researchers, open conflicts were solved and coded under consideration of the International Socio-Economic Index (ISEI) score [35].

#### 2.2.4. Age and Anthropometric Measures

Participants reported birth date before a research assistant assessed anthropometric data using a weight scale and stadiometer.

### 2.3. Procedures

The assessments were conducted at the beginning of the PE lessons. Individual codes ensured the anonymity of the participants. With regard to a previously defined protocol, research employees gave instructions to fill out the questionnaire and supervised the pupils during process time in order to answer any questions about the questionnaire without disturbing the other students. The PE class started after the research employee collected all completed questionnaires.

#### 2.3.1. Recruitment Procedure

The Ethics Committee of the Technical University of Munich in Germany approved the study, registered with 155/16S. Principals and parents councils approved the assessments in schools. Parents or legal guardians as well as children gave written informed consent to participate in the study.

#### 2.3.2. Statistical Analysis

Taking into account the clustered nature of the sample, we considered Huang’s [30] multilevel approach to analyze the data. Based on Hox’ five MCFA steps [29], Huang [30] provides the R code [36] to analyze the individual (named level 1 or within-group level) and the group level (named level 2 or between-group level) simultaneously, using the lavaan package [37].

Firstly, an adjusted single-level CFA was conducted under consideration of the pooled within-group covariance matrix instead of the total covariance matrix. In a second step, we specified the null model, ergo the factor structure of step 1 on both levels, using the pooled within- and the between-group covariance matrices. Here, we constrained equal factor loadings, variances and covariances for every manifest variable and latent factor. Thirdly, we incorporated new group-level latent variables with denial to covary in the so-called independence model, to estimate the variance at group level. In step 4, we reversed the denial and used all degrees of freedom at the between-group level to create a fully saturated model. As a last step, we specified the actually hypothesized models. Initially, including one latent factor to the between-group level ensured the correlation of the latent group-level factors [30].

In addition, exploring the scale dimensionality justifies model structures with one or three latent factors at level 2. Some estimated residual variances for the random intercepts at level 2 were negative but also close to zero. These variances were fixed to zero to allow the model to converge and find admissible solutions. This procedure is justified due to the small sample size at level 2 and intraclass correlations (ICCs) close to zero [29].

Several fit indices were adduced to evaluate the goodness of fit for the model, since all of them have limitations regarded separately [38]: the χ^2^ likelihood ratio statistic, the comparative fit index (CFI) [39], the Tucker–Lewis index (TLI), the root mean square error of approximation (RMSEA) and its associated 90% confidence interval [40], and the standardized root mean square residual (SRMR). Hu and Bentler’s reference work was applied to judge fit indices [41]. Thereby, CFI and TLI values greater than 0.95, RMSEA and SRMR values less than 0.08, support a good model fit [41]. For comparison of alternative models, the Akaike information criterion [42] was applied, which indicates better fitting models by smaller values.

Reliability scores for the scales at both levels respectively were calculated by the alpha function from the psych package [43]. Since the estimated pooled within the matrix was not positive definite, we computed the nearest positive definite matrix, using function nearDP from the Matrix package [44], to avoid miscalculated alpha scores. Correlations on both levels were investigated to analyze the contributions of each subscale predicting MVPA, using the statsBy function from the psych package [43]. Correlations of SES, body mass index (BMI) and age were controlled by using the rcorr function by the Hmisc package [45]. Whether missings were completely at random (MCAR) was investigated by using the Little’s MCAR test [46] executed by the LittleMCAR function from the BaylorEdPsych package [47]. A two-sided significance level of <0.05 was set for all analysis.

## 3. Results

Proportion of missing values from 1.04% to 2.50% and Little’s MCAR test (χ^2^ = 259.12, df = 310, *p* = 0.99) support that the missings are completely at random [46]. The average BMI value of 19.49 (±3.68, interquartile range (IQR) = 4.68, *N* = 386) kg/m² reflects a normal-weight sample. Responses of students whose height and weight were measured did not differ significantly from students, both apparently overweight and normal-weight girls, which refused to be weighed. Participants come from households with an average SES of 49.80 (±15.96, median = 48, IQR = 25.00, *N* = 412).

Table 1 shows descriptive statistics of all German items, including an explanatory back-translation to English. Items’ means ranged from 3.02 (±1.01) to 4.18 (±0.95). Average standard deviation is 1.03. Skewness and kurtosis values were low to moderate. ICCs vary from zero to 0.21 with an average of 0.04 (SD = 0.07; median = 0.011). We set three negative ICCs to zero, since the ICC should vary between 0 and 1 by definition:

(1) $$\rho ={\left(\Sigma_{B}^{2}+\Sigma_{W}^{2}\right)}^{-1}\;\Sigma_{B}^{2},$$

where $\Sigma_{W}^{2}$ represents the within-group variance. Because the between-group variance $\Sigma_{B}^{2}$ is estimated by a scaled difference between two diagonal entries of two empirical covariance matrices (the empirical within- and between-covariance matrices), it does not have to be positive for any sample size. Here, the estimated within-variance is larger than the estimated between-variance and led to negative ICCs.

Firstly, we evaluated the fitted models of the 12-items scale that converged to an admissible solution. In all models, item R4 had the lowest factor loadings. In addition, the distribution of the item was right-skewed, which contradicts the assumption of the model. Inspection of the item explained the right-skewness due to social desirability. Therefore, we removed R4.

In the following, we only report the analysis of the reduced scale because even the best model fit of the 12-item scale did not pass the cut-point criteria comprehensively [41]. Table 2 represents the fit indices for the five steps, including three hypothesized models that converged to an admissible solution fitted to the reduced scale data.

Representing step 1, the level one model with three factors at a single-level showed acceptable fit indices, except of the TLI, which is below 0.95. Poor fit of the null model allowed the tentative assumption that there might be a between-group variance. In addition, the independence model did not fit well. Meaning, that there might be a substantively interesting structural model and a substantial group-level variance. Resulting fits of the saturated model are, except CFI, quite poor. Since we can rule out any error, this indicates that the initial fit in step 1 was too poor. Nonetheless, we were interested in modeling the relationships at level 2 and legitimized the further MCFA due to the fact that Muthén et al. forwent the preliminary steps 2, 3 and 4 by Huang [30] in his MCFA procedure [18]. Moreover, “even slight departures [of the ICC] from zero can signify that the multilevel nature of data should be accounted for” [28] (p. 8).

Model A has moderate CFI, TLI and a RMSEA close to the reference cut-point, but SRMR of 0.12 is clearly out of bounds. Model B did not meet the criteria for acceptable model fit, since TLI is below 0.90 and RMSEA higher than 0.08. The reduced scale fitted Model C well. CFI and TLI are close to the cut point of 0.95, RMSEA is below 0.08, although the 90% confidence interval exceeds 0.09, as well as the SRMR value is way below 0.08. Chi-square difference tests revealed a significant preeminence of Model C.

Models with freely estimated loadings, variances and covariances did not converge to a solution for all tested factor structures. A model of type B with equality constraints for the within- and the between-group level, meaning that the same estimates are used at level 1 and 2, did not yield any admissible solution, since negative residual variances remained although variances were set to zero. A model of type C with a nested model structure, which means that the measurement model of level 1 is also included at level 2, revealed acceptable goodness of fit but most factor loadings were not significant and some loadings and covariances were not computed due to negative residual variances.

Table 3 presents standardized factor loadings and correlations of latent factors for the supported Model C. Factor loadings were significant and ranged from 0.54 to 0.90 at individual level and from 0.64 to 0.96 at class level. Significant correlates between factors vary from 0.37 to 0.60. Small non-significant correlations at the between level between factors appeared.

Reliability scores for the subscales at level 1 were adequate to good, ranging from 0.78 to 0.85 (see Table 1). At level 2, the subscales differ in reliability. At class level, an adequate score, α = 0.79, for relatedness and excellent score, α = 0.95, for autonomy could be derived, however, competence subscale drops off to α = 0.18. Problematic items such as R3 and C3 correlate with the subscale negatively and were reversed automatically. Composite reliability for the total scale was α = 0.85 at the individuals level and α = 0.84 at the class level.

On average, girls spent 80.44 (±21.01) minutes in MVPA per day (*N* = 374). Small significant correlations with device-based assessed MVPA can be evinced at level 1 with 481 individuals. While correlations of autonomy and competence subscale were significant by 0.13 (*p* = 0.01) and 0.19 (*p* > 0) respectively, there was no significant correlation of MVPA with the relatedness subscale (r = 0.03, *p* = 0.51). At group level (*n* = 33), the subscales competence, by 0.28 (*p* = 0.12), and relatedness, by 0.25 (*p* = 0.16), showed smaller non-significant correlations while autonomy correlated significantly by 0.38 (*p* = 0.03). There was no significant correlation between SES and MVPA (*ρ* = 0.06, *p* = 0.26), while BMI and age showed a negligible significant correlation of r = −0.14 (*p* < 0.01) and r = −0.16 (*p* < 0.01), respectively.

## 4. Discussion

Low levels of PA and high sedentary time of children and adolescents in industrialized countries reveal the need to understand motivational tendencies and behavior in order to increase the number of effective and economic interventions promoting PA in all stages of life. One auspicious approach is set by the solid theoretical foundation of SDT, already applied in several contexts and domains [9]. Specifically, BPN-supportive elements in PE increase students’ BPNS and in a further step, PA [48]. The evaluation of those mechanisms of PA behavior change is in need of validated measurement tools for specific contexts and samples.

This study validated the GBPNS-PE. In detail, indices for factor structure, internal consistency and criterion validity as well as scale dimensionality were determined. Considering the clustered data structure, we conducted a MCFA. Occurrence of inconclusive fit indices of the saturated model is a matter of interpretation. The ambiguous definition of the MCFA procedure raised concerns at this point.

Comparing the German sample with the other validation samples exhibits some differences in terms of items means and standard deviations regarding the BPNS. The item mean of 3.56 derived by the 9-item scale is comparable to the Dutch sample (M = 3.21) by Haerens et al. [20] but lower than the original validation with late adolescents by Chen et al. [19] in a US sample (M = 4.01) and a Belgium sample (M = 3.91). Standard deviations of those latter three samples vary from 0.73, 0.72 to 0.61, respectively, being lower than 1.03 of the German sample.

Our subscales had lower reliability values (0.76–0.84) as the original BPNS scale achieved higher alpha scores of 0.81 to 0.92 [22]. Additionally, the 12 satisfaction items of the BPNSFS achieved higher alpha scores ranging from 0.81 to 0.88 [19]. Moreover, our overall alpha score (0.85) is somewhat lower than Haerens et al.’s [20] scale with α = 0.87, even though the 11-item scale and each subscale at level 1 showed satisfactory reliability scores. One strength of the MCFA is the estimation of reliability scores at level 2. As aforementioned alpha scores at level 2 (0.95, 0.65, 0.18) differ from level 1 (0.78, 0.79, 0.85), especially the low score for the competence subscale at the class level could be most likely explained by a lack of variability on level 2, indicated by low ICC values.

The large within-class variability of the relatedness and competence construct implicates that classes with few participants produce scores with low reliabilities at the class level. In support of this contention is the lower within-class variability of the construct autonomy. Seemingly, the girls within one class appraise autonomy to the same extent, while girls valuate the constructs relatedness and competence differently throughout the class. Probably justifiable due to group-dynamic processes influencing the climate of each class, the reliability score decreases at the group level. We interpret the decrease of the competence reliability scores at level 2 due to heterogeneous classes in terms of sports prowess. Talented girls are often high-performers in their classes in PE, while in other classes, the overall physical performance of girls might be weaker. Moreover, even though the curriculum puts PE into a frame, the teacher determines the demands of challenges in PE lessons. These demands differ from teacher to teacher, ergo from class to class.

The main reason to prefer the 11-item scale is due to the improved goodness of fit in comparison to the 12-item scale under consideration of the model assumptions. An explorative reduction of items (e.g., A3 and C3) improved the goodness of fit of all models but at the same time, this procedure cannot be justified due to a mainly nontransparent reduction of significant items resulting in an over-estimation of the model.

Model A could be used to interpret the data, since it represents the procedure of an adjusted single-level CFA [30]. However, expanding the thoughts towards a three-factor solution at two levels resulted in an almost equivalent fit by refraining a specification of a nested structure. In line with theoretical considerations of SDT, the three-factor structure at the individual level of the BPNS scale was also confirmed by previous findings [19,20]. The separated structure of three latent factors on both levels makes Model C preferable because the cut-point criteria are nearly surpassed. Despite two intercorrelations of two latent factors at level 2, we showed the equivalence of the three-factor structure explicitly across levels. Subsequently, we assume that both within and between classes, the satisfaction of autonomy, relatedness and competence are perceived as three distinct constructs in PE. Furthermore, Model C provides detailed information on the three factors at the class level, contrary to a one-factor solution at level 2 and its parsimonious summary.

While SES seems to be not related to MVPA, our data indicate a weak negative relationship of BMI and age with MVPA. However, current literature states a contradictory position and underlines the need to incorporate social and environmental factors in the analysis of PA behavior of youth by an adequate analysis [2]. Especially the complexity of motivational behavior and its mediation and moderation effects foster a comprehensive analysis. Therefore, we confine the purpose of the present study to the validation of the GBPNS-PE and seek to examine intervention effects of the CReActivity study in a future work.

### Strengths and Limitations

This study provides detailed indications of the psychometric properties of GBPNS-PE for a specific target group in the school setting by an MCFA procedure. Nonetheless, an extension to other age groups, sex/gender and consideration of demographic domains would support generalizability of the psychometric properties of the scale, especially the incorporation of frustration items is sought in future investigations.

Three classes with less than seven individuals remained in the sample to retain a sufficient sample size on level 2, although clusters with few observations could bias the estimates and reliability scores [49]. We considered several clusterings (schools, regional clustering, and exclusion of small classes) for all models, though negligible changes in model fits and reliability scores were observable.

## 5. Conclusions

This validation study provides initial proof for the three-factor structure of the BPNS scale in a multilevel design. Facing the limited periods available to assess comprehensive data in the school setting, the GBPNS-PE is an efficient solution to evaluate the need satisfaction of students in PE. Further investigations with a validation sample would establish the GBPNS-PE as a valid measurement tool in the German-speaking area and contribute to a higher robustness of the scale.

## Figures and Tables

**Table 1:** Descriptive statistics for the items of the German Basic Psychological Need Satisfaction in Physical Education Scale.

Items	M	SD	Skewness	Kurtosis	ICC
**Autonomy (Composite Reliability: 4 items = 0.78)**					
**A1:** …können wir uns regelmäßig aussuchen, was wir machen möchten. (…we can regularly choose what we like to do.)	3.02	1.01	0.17	−0.01	0.219
**A2:** …machen wir häufig genau das, was ich wirklich machen will. (…we often do exactly what I really like to do.)	3.06	1.06	0.03	−0.47	0.059
**A3:** …lerne ich Sportarten, die wirklich gut zu mir passen. (…I learn sports, which really suit me.)	3.55	1.17	-0.32	−0.74	0.060
**A4:** …machen wir häufig das, was mich wirklich interessiert. (…we often do what really interests me.)	3.29	1.05	-0.09	−0.44	0.103
**Relatedness (Composite Reliability: 3 items = 0.79)**					
**R1:** …habe ich das Gefühl, dass die Klassenkameradinnen, die ich mag, auch mich mögen. (…I have the feeling the classmates that I like, also like me.)	4.04	1.00	−0.86	0.17	0.011
**R2:** …fühle ich mich mit den Klassenkameradinnen verbunden, die mich mögen und die ich auch mag. (…I feel connected with the classmates that like me and I like.)	3.88	1.05	−0.67	−0.25	0 **
**R3:** …fühle ich mich mit Klassenkameraden verbunden, die mir wichtig sind. (…I feel connected with the classmates who are important to me.)	3.97	1.07	−0.88	0.11	0 **
R4 *: …verstehe ich mich mit meinen Klassenkameradinnen sehr gut. (…I get along with my classmates very well.)	4.18	0.95	−0.98	0.42	-
**Competence (Composite Reliability: 4 items = 0.85)**					
**C1:** …bin ich gut. (…I am good.)	3.87	0.95	−0.48	−0.31	0.004
**C2:** …fühle ich mich talentiert. (…I feel talented.)	3.28	1.12	−0.25	−0.53	0 **
**C3:** …schaffe ich das, was ich mir vorgenommen habe. (…I can achieve what I aimed for.)	3.68	1.00	−0.36	−0.31	0.023
**C4:** …kann ich auch schwierige Aufgaben meistern. (…I can master difficult tasks.)	3.66	0.98	−0.28	−0.31	0.006

Note: *N* = 481; M = Mean; SD = standard deviation; ICC = intraclass correlation; * excluded in reduced scale ** per definition.

**Table 2:** Summary of the goodness of fit indices.

Fit index	Step 1	Step 2	Step 3	Step 4	Step 5		
	Level one model: three factors at single level	Null model	Independence model	Saturated model	A: Three factors on level 1, one factor on level 2, nested	B: Three factors on level 1, one factor on level 2, not nested	C: Three factors on both levels, not nested
**Short Scale (11 items)**							
χ^2^	124.159	273.515	244.328	142.171	209.600	253.129	186.377
df	41	107	97	42	86	87	82
χ^2^/df	3.03	2.56	2.51	3.38	2.44	2.91	2.27
CFI	0.954	0.918	0.927	0.950	0.939	0.918	0.948
TLI	0.939	0.915	0.917	0.870	0.922	0.896	0.931
RMSEA	0.071	0.085	0.084	0.105	0.081	0.094	0.077
90% conf	0.057–0.86	0.072–0.97	0.071–0.097	0.086–0.124	0.068–0.096	0.081–0.107	0.062–0.091
SRMR	0.054	0.091	0.136	0.104	0.119	0.065	0.058
AIC	11,021.06	12,058.25	12,049.06	12,056.90	12,036.33	12,077.86	12,021.11

Note: df = degrees of freedom; CFI = comparative fit index; TLI = Tucker–Lewis index; RMSEA = root mean square error of approximation; conf = confidence interval; SRMR = standardized root mean square residual; AIC = Akaike’s information criterion.

**Table 3:** Completely standardized factor loadings and correlations of latent factors for Model C: Three factors at level 1 and three factors at level 2, not nested.

		Level 1 (Individuals)			Level 2 (Classes)		
Factor	Item	F1	F2	F3	F1	F2	F3
Autonomy	A1	0.538			0.809		
	A2	0.720			0.904		
	A3	0.633			0.880		
	A4	0.871			0.961		
Relatedness	R1		0.587			0.644	
	R2		0.896			0.957	
	R3		0.773			0.670	
Competence	C1			0.847			0.890
	C2			0.801			0.942
	C3			0.677			0.920
	C4			0.765			0.876
Cor(F1,F2)		0.366			0.199 ^a^		
Cor(F1,F3)		0.595			0.561		
Cor(F2,F3)		0.416			0.413 ^a^		

^a^ Note: All loadings were significant at *p* < 0.05, except marked with.

## Supplementary Materials

The following are available online at https://www.mdpi.com/1660-4601/17/5/1554/s1. Dataset and R code are provided as supplementary material.
