# Supplementary material for: The German Basic Psychological Needs Satisfaction in Physical Education Scale: Adaption and Multilevel Validation in a Sample of Sixth-Grade Girls
Source: Int J Environ Res Public Health. 2020 Feb 28;17(5):1554. doi: 10.3390/ijerph17051554 (PMC7084701; doi:10.3390/ijerph17051554)
Supplement: Supplementary file 1 [file ijerph-17-01554-s001.zip › MCFA_GBPNS-PE-Scale.pdf]

MCFA_GBPNS-PE Scale

DS

15 Januar 2020

MCFA and reliability analysis, 11 items: R4 excluded

Load Packages

```
library(lavaan)
library(tidyverse)
library(Matrix)
library(psych)
```

```
# Load dataset AKS_U_Skala
AKS_PE <- read.csv2(file.choose())

##### Delete R4.
AKS_PE <- AKS_PE[,-c(9)]

# Delete empty cases.
maxNA <- 7
AKS_PE <- AKS_PE[!(base::rowSums(is.na(AKS_PE))>maxNA),]
```

Daten

Zur Veranschaulichung des Datensatzes

```
## 'data.frame':    481 obs. of  12 variables:
##  $ Gruppe          : int  5 5 5 5 5 5 5 5 5 5 ...
##  $ Autonomy_PE_1   : int  2 1 4 3 2 1 2 3 3 3 ...
##  $ Autonomy_PE_2   : int  2 2 3 3 3 1 3 2 4 2 ...
##  $ Autonomy_PE_3   : int  4 2 3 3 5 1 3 5 3 3 ...
##  $ Autonomy_PE_4   : int  4 2 4 4 4 1 3 2 4 3 ...
##  $ Relatedness_PE_1: int  5 5 5 5 5 5 4 5 3 3 ...
##  $ Relatedness_PE_2: int  2 5 5 4 5 5 4 5 3 3 ...
##  $ Relatedness_PE_3: int  2 5 5 3 5 5 4 5 3 3 ...
##  $ Competence_PE_1 : int  4 5 5 5 5 1 4 5 2 3 ...
##  $ Competence_PE_2 : int  3 4 4 4 5 1 2 3 2 3 ...
##  $ Competence_PE_3 : int  1 5 3 4 5 1 4 5 2 3 ...
##  $ Competence_PE_4 : int  5 4 1 4 5 1 4 4 3 2 ...
```

```
##      Gruppe      Autonomy_PE_1   Autonomy_PE_2   Autonomy_PE_3
##  Min.   : 1.00   Min.   :1.000   Min.   :1.000   Min.   :1.000
##  1st Qu.: 9.00   1st Qu.:2.000   1st Qu.:2.000   1st Qu.:3.000
##  Median :17.00   Median :3.000   Median :3.000   Median :3.000
##  Mean   :17.05   Mean   :3.015   Mean   :3.057   Mean   :3.546
##  3rd Qu.:25.00   3rd Qu.:3.000   3rd Qu.:4.000   3rd Qu.:5.000
##  Max.   :33.00   Max.   :5.000   Max.   :5.000   Max.   :5.000
##                  NA's   :5       NA's   :7       NA's   :5
##  Autonomy_PE_4   Relatedness_PE_1 Relatedness_PE_2 Relatedness_PE_3
##  Min.   :1.000   Min.   :1.000    Min.   :1.000    Min.   :1.00
##  1st Qu.:3.000   1st Qu.:3.000    1st Qu.:3.000    1st Qu.:3.00
##  Median :3.000   Median :4.000    Median :4.000    Median :4.00
##  Mean   :3.279   Mean   :4.038    Mean   :3.878    Mean   :3.96
##  3rd Qu.:4.000   3rd Qu.:5.000    3rd Qu.:5.000    3rd Qu.:5.00
##  Max.   :5.000   Max.   :5.000    Max.   :5.000    Max.   :5.00
##  NA's   :11      NA's   :9        NA's   :7        NA's   :10
##  Competence_PE_1 Competence_PE_2 Competence_PE_3 Competence_PE_4
##  Min.   :1.000   Min.   :1.000   Min.   :1.000   Min.   :1.000
##  1st Qu.:3.000   1st Qu.:3.000   1st Qu.:3.000   1st Qu.:3.000
##  Median :4.000   Median :3.000   Median :4.000   Median :4.000
##  Mean   :3.859   Mean   :3.279   Mean   :3.684   Mean   :3.656
##  3rd Qu.:5.000   3rd Qu.:4.000   3rd Qu.:4.000   3rd Qu.:4.000
##  Max.   :5.000   Max.   :5.000   Max.   :5.000   Max.   :5.000
##  NA's   :12      NA's   :8       NA's   :12      NA's   :7
```

MCFA

```
print(AKS_PE %>%
  group_by(Gruppe) %>%
  summarise(obs_per_class = n()) , n = Inf)
```

```
## # A tibble: 33 x 2
##    Gruppe obs_per_class
##     <int>         <int>
##  1      1            22
##  2      2            12
##  3      3            22
##  4      4            23
##  5      5            14
##  6      6             4
##  7      7             4
##  8      8            18
##  9      9             8
## 10     10             5
## 11     11            13
## 12     12            18
## 13     13             9
## 14     14            24
## 15     15            20
## 16     16            11
## 17     17            15
## 18     18             9
## 19     19            23
## 20     20            19
## 21     21            16
## 22     22            14
## 23     23            12
## 24     24            19
## 25     25             7
## 26     26            13
## 27     27            26
## 28     28            11
## 29     29            11
## 30     30            11
## 31     31            13
## 32     32            19
## 33     33            16
```

```
print(AKS_PE %>%
  group_by(Gruppe) %>%
  summarise_all(funs(mean = mean(., na.rm = T))) , width = Inf, n = Inf)
```

```
## # A tibble: 33 x 12
##    Gruppe Autonomy_PE_1_mean Autonomy_PE_2_mean Autonomy_PE_3_mean
##     <int>              <dbl>              <dbl>              <dbl>
##  1      1               3.09               3.09               3.59
##  2      2               3.42               3.5                3.83
##  3      3               2.68               3                  3.32
##  4      4               3.91               3.39               3.74
##  5      5               2.5                2.57               3.5
##  6      6               2.25               2.5                3.75
##  7      7               4                  3.25               4.25
##  8      8               3.06               2.89               3.67
##  9      9               3.38               3.38               4.12
## 10     10               2.6                3                  3.4
## 11     11               2.69               3.46               3.77
## 12     12               3.33               2.94               3.44
## 13     13               3.89               3.44               3.44
## 14     14               2.74               2.67               3.25
## 15     15               2.65               3.2                3.58
## 16     16               3.6                2.73               3.64
## 17     17               3.71               3.43               4.21
## 18     18               3.33               2.88               3.78
## 19     19               3.05               3.23               3.65
## 20     20               1.61               2.11               2.53
## 21     21               3.19               2.79               3.33
## 22     22               3.29               3.43               3.93
## 23     23               3.42               3.67               4.5
## 24     24               2.47               2.53               2.53
## 25     25               4                  3.86               4
## 26     26               2.77               2.92               3.17
## 27     27               3.15               3.16               3.76
## 28     28               2.55               3.18               3.45
## 29     29               2.82               3.1                3.91
## 30     30               4                  3.73               3.91
## 31     31               2.85               3                  3.23
## 32     32               2.42               3.11               3.47
## 33     33               2.81               2.81               3.44
##    Autonomy_PE_4_mean Relatedness_PE_1_mean Relatedness_PE_2_mean
##                 <dbl>                 <dbl>                 <dbl>
##  1               3.55                  3.64                  3.55
##  2               3.75                  3.5                   3.75
##  3               3.32                  4.05                  3.73
##  4               3.61                  4.26                  3.83
##  5               3.14                  4.36                  4
##  6               3                     4                     4.5
##  7               3.25                  3.5                   3.75
##  8               3.39                  4.39                  4.17
##  9               3.88                  3.88                  3.75
## 10               2.8                   4                     3.8
## 11               3.54                  4.38                  3.69
## 12               3.06                  4.28                  4
## 13               3.44                  3.89                  3.89
## 14               3.18                  3.75                  3.79
## 15               3.22                  4.05                  4.16
## 16               3.09                  3.91                  3.82
## 17               3.86                  4.07                  3.87
## 18               3.33                  3.78                  3.67
## 19               3.43                  4.36                  4.05
## 20               2.11                  4.06                  3.95
## 21               2.43                  3.75                  3.6
## 22               3.64                  3.71                  3.93
```

```
## 23                3.83                  4.25                  4.27
## 24                2.74                  3.84                  3.53
## 25                4.14                  4.57                  4
## 26                2.83                  3.77                  3.54
## 27                3.32                  4.31                  4.04
## 28                3.36                  4.3                   4.3
## 29                3.36                  3.91                  4
## 30                4.18                  4.36                  3.91
## 31                3.08                  3.46                  3.69
## 32                3.21                  4.42                  4.26
## 33                2.94                  3.87                  3.53
##    Relatedness_PE_3_mean Competence_PE_1_mean Competence_PE_2_mean
##                    <dbl>                <dbl>                <dbl>
##  1                   3.9                  3.64                 3.05
##  2                   3.92                 4                    3.58
##  3                   3.91                 3.95                 3.23
##  4                   3.83                 4                    3.10
##  5                   3.93                 3.86                 3.36
##  6                   3.5                  3.75                 3.25
##  7                   4                    3.5                  3.75
##  8                   4.22                 3.61                 2.72
##  9                   4                    4.38                 3.88
## 10                   4                    4                    3.8
## 11                   3.77                 4                    3.46
## 12                   3.89                 4.24                 3.67
## 13                   3.89                 3.88                 3
## 14                   3.83                 3.79                 3.25
## 15                   4                    3.9                  3.32
## 16                   3.91                 3.91                 2.9
## 17                   4.2                  4.08                 4
## 18                   3.88                 4.11                 3.38
## 19                   4.38                 4.09                 3.68
## 20                   4.16                 3.5                  2.79
## 21                   3.81                 3.94                 3
## 22                   4.07                 3.43                 3.14
## 23                   4.08                 4.33                 4
## 24                   3.63                 3.32                 2.89
## 25                   4.29                 3.43                 3.29
## 26                   3.67                 3.83                 3.38
## 27                   4.08                 4.12                 3.19
## 28                   4.2                  3.82                 3.09
## 29                   3.91                 3.64                 3.09
## 30                   3.73                 3.55                 3.18
## 31                   3.83                 3.92                 3.38
## 32                   4.21                 4.11                 3.42
## 33                   3.69                 3.62                 3.25
##    Competence_PE_3_mean Competence_PE_4_mean
##                   <dbl>                <dbl>
##  1                 3.41                 3.45
##  2                 3.67                 3.83
##  3                 3.5                  3.59
##  4                 3.83                 3.52
##  5                 3.64                 3.5
##  6                 4                    3.5
##  7                 3.67                 3.5
##  8                 3.89                 3.44
##  9                 4                    4
## 10                 3.8                  3.6
## 11                 4.15                 4.08
## 12                 3.72                 4.11
```

```
## 13                      4.11                    3.33
## 14                      3.46                    3.79
## 15                      4                       3.8
## 16                      3.7                     3.64
## 17                      3.93                    4
## 18                      3.67                    4.22
## 19                      4.05                    3.86
## 20                      3.39                    3.26
## 21                      3.07                    3.21
## 22                      3.5                     3.79
## 23                      4                       3.91
## 24                      3.26                    3.32
## 25                      4.14                    3.14
## 26                      3.54                    3.85
## 27                      3.8                     3.65
## 28                      3.6                     3.82
## 29                      4.09                    3.91
## 30                      3.55                    3.27
## 31                      3.23                    3.23
## 32                      4                       3.84
## 33                      3.19                    3.6
```

Define functions for MCFA

```
# Execute function mcfa.input with grouping variable and safe to object x
x <- mcfa_input("Gruppe", AKS_PE)
```

```
# Graphical representation of the pure within and between covariance matrix
melted_pw.cov <- reshape2::melt(x$pw.cov)
melted_b.cov <- reshape2::melt(x$b.cov)

ggplot(data = melted_pw.cov, aes(x=Var1, y=Var2, fill=value)) +
  geom_tile()

ggplot(data = melted_b.cov, aes(x=Var1, y=Var2, fill=value)) +
  geom_tile()
```

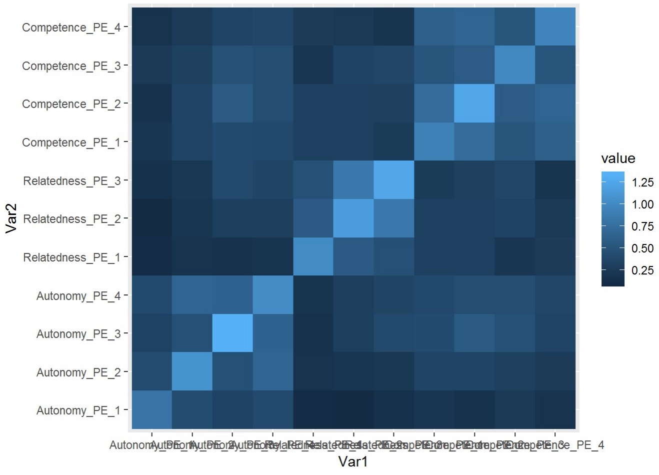

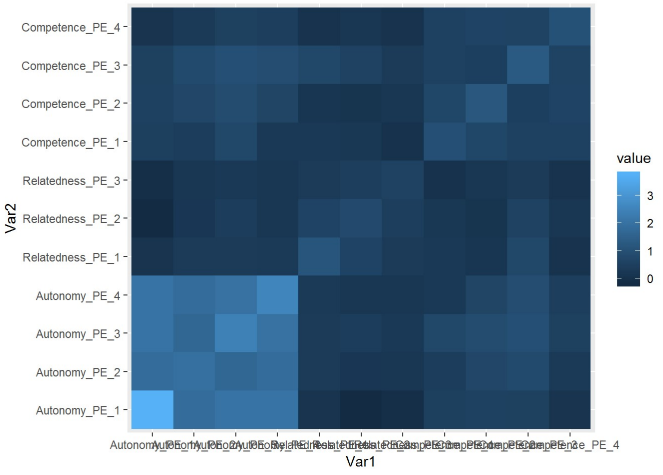

Indicates three factors at individual level but maybe only one factor at group level.

```
str(x)
```

```
## List of 10
##  $ b.cov : num [1:11, 1:11] 3.747 1.863 2.039 2.045 0.112 ...
##   ..- attr(*, "dimnames")=List of 2
##   .. ..$ : chr [1:11] "Autonomy_PE_1" "Autonomy_PE_2" "Autonomy_PE_3" "Autonomy_PE_4" ...
##   .. ..$ : chr [1:11] "Autonomy_PE_1" "Autonomy_PE_2" "Autonomy_PE_3" "Autonomy_PE_4" ...
##  $ pw.cov: num [1:11, 1:11] 0.805 0.428 0.344 0.41 0.106 ...
##   ..- attr(*, "dimnames")=List of 2
##   .. ..$ : chr [1:11] "Autonomy_PE_1" "Autonomy_PE_2" "Autonomy_PE_3" "Autonomy_PE_4" ...
##   .. ..$ : chr [1:11] "Autonomy_PE_1" "Autonomy_PE_2" "Autonomy_PE_3" "Autonomy_PE_4" ...
##  $ ab.cov: num [1:11, 1:11] 0.225466 0.110042 0.129883 0.125318 0.000456 ...
##   ..- attr(*, "dimnames")=List of 2
##   .. ..$ : chr [1:11] "Autonomy_PE_1" "Autonomy_PE_2" "Autonomy_PE_3" "Autonomy_PE_4" ...
##   .. ..$ : chr [1:11] "Autonomy_PE_1" "Autonomy_PE_2" "Autonomy_PE_3" "Autonomy_PE_4" ...
##  $ n     : int 433
##  $ G     : int 33
##  $ c.    : num 13
##  $ sqc   : num 3.61
##  $ icc   : Named num [1:11] 0.219 0.059 0.06 0.103 0.011 -0.023 -0.043 0.004 -0.002 0.023
...
##   ..- attr(*, "names")= chr [1:11] "Autonomy_PE_1" "Autonomy_PE_2" "Autonomy_PE_3" "Auton
omy_PE_4" ...
##  $ dfw   : int 400
##  $ dfb   : int 33
```

Calculate ICCs as indicator for the variance, which can be attributed to the the groups.

```
x$icc
```

```
##    Autonomy_PE_1    Autonomy_PE_2    Autonomy_PE_3    Autonomy_PE_4
##            0.219            0.059            0.060            0.103
## Relatedness_PE_1 Relatedness_PE_2 Relatedness_PE_3  Competence_PE_1
##            0.011           -0.023           -0.043            0.004
##  Competence_PE_2  Competence_PE_3  Competence_PE_4
##           -0.002            0.023            0.006
```

ICCs vary heavily.

```
max(x$icc)
```

```
## [1] 0.219
```

minimal

```
min(x$icc)
```

```
## [1] -0.043
```

```
mean(c(0.219,0.059,0.060,0.103,0.011,0,0,0.004,0,0.023,0.006))
```

```
## [1] 0.04409091
```

```
median(c(0.219,0.059,0.060,0.103,0.011,0,0,0.004,0,0.023,0.006))
```

```
## [1] 0.011
```

```
sd(c(0.219,0.059,0.060,0.103,0.011,0,0,0.004,0,0.023,0.006))
```

```
## [1] 0.06705886
```

Note negative ICCs. Three negative ICCs were set to zero, since the ICC

$$\rho = (\Sigma_B^2 + \Sigma_W^2)^{-1} * \Sigma_B^2$$

should vary between 0 and 1 by definition. Due to the fact that the between group variance $??_B^2$ is estimated by a scaled difference between two diagonal entries of two empirical covariance matrices (the empirical within and between covariance matrices), it does not have to be positive for any sample size. Here, the estimated within-variance is larger than the estimated between-variance and led to negative ICCs.

Graphical analysis

```
AKS_PE_aut <- AKS_PE %>%
  select(starts_with("Auto"))

ggplot(gather(AKS_PE_aut), aes(x = value)) + geom_bar() + facet_wrap(~ key)
```

```
## Warning: Removed 28 rows containing non-finite values (stat_count).
```

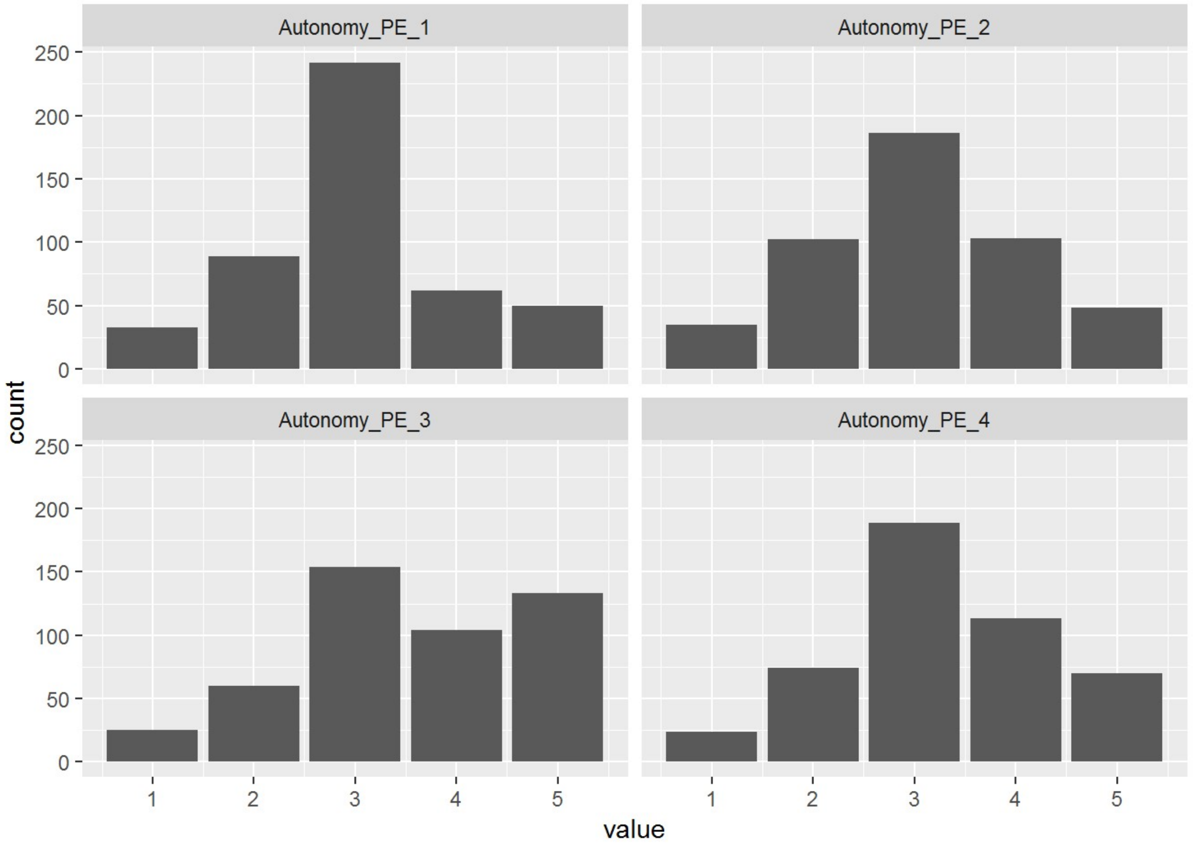


```
AKS_PE_comp <- AKS_PE %>%
  select(starts_with("Comp"))

ggplot(gather(AKS_PE_comp), aes(x = value)) + geom_bar() + facet_wrap(~ key)
```

```
## Warning: Removed 39 rows containing non-finite values (stat_count).
```

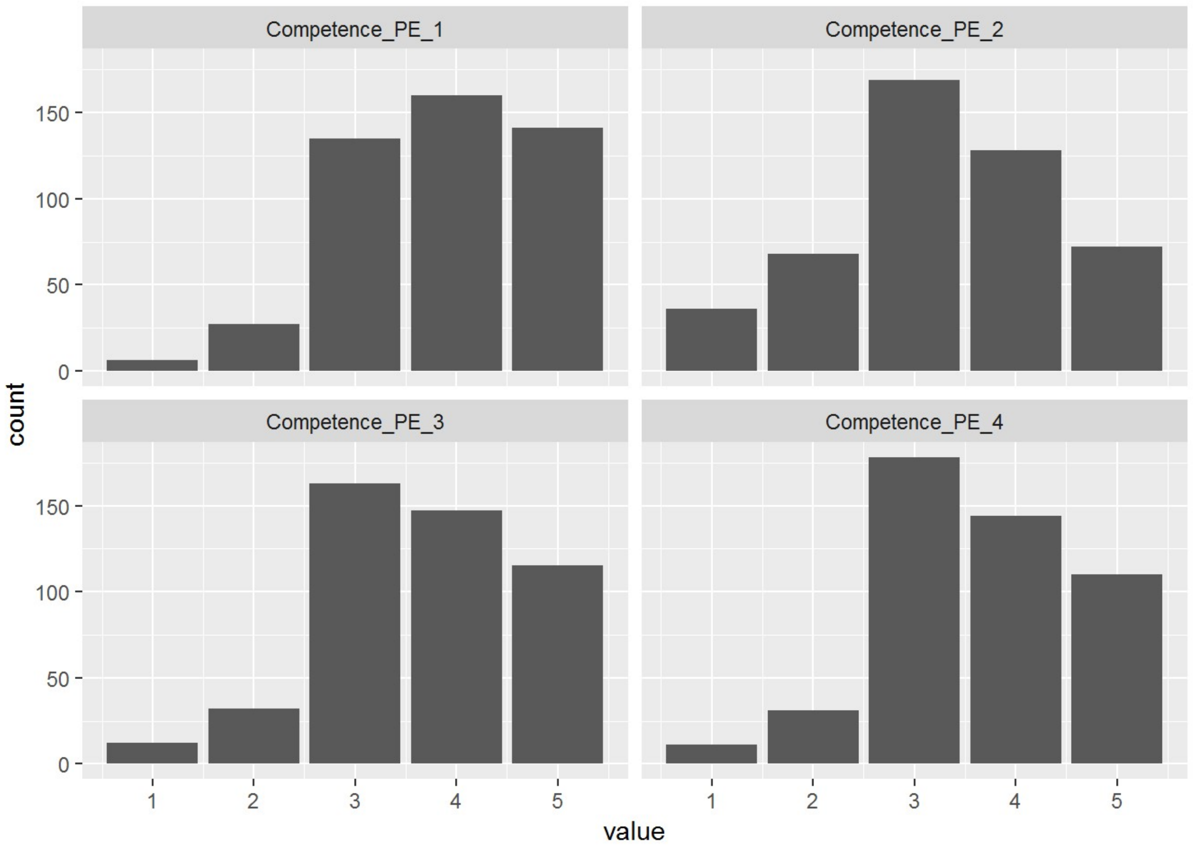


```
AKS_PE_related <- AKS_PE %>%
  select(starts_with("Related"))

ggplot(gather(AKS_PE_related), aes(x = value)) + geom_bar() + facet_wrap(~ key)
```

```
## Warning: Removed 26 rows containing non-finite values (stat_count).
```

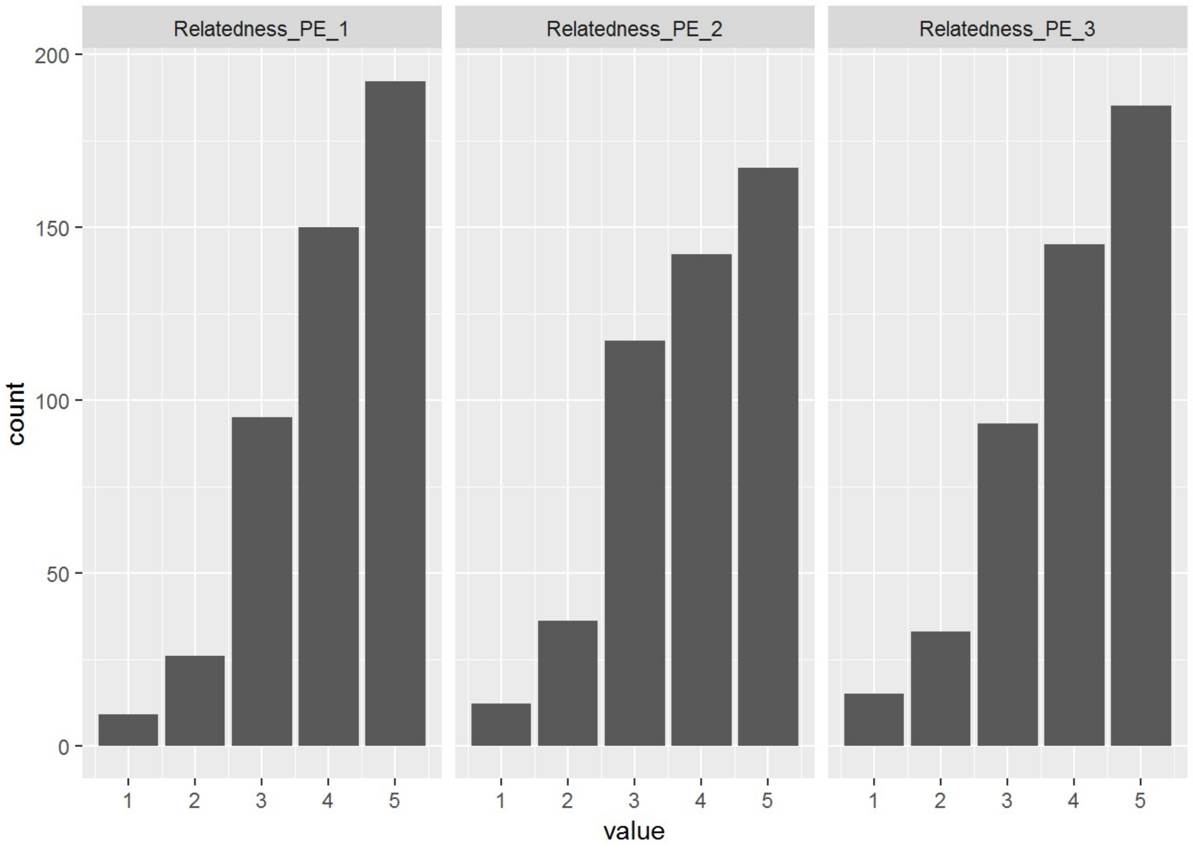

The data is clearly unbalanced. Hence $\mathbf{S}_{Bd}$ is an estimator of

$$\mathbf{\Sigma}_W + c_d \mathbf{\Sigma}_B$$

for each distinct group of size n_d.

This means that one gets a different covariance decomposition for each different group size, which makes estimating these models a very complex task. Therefore Muthen (1989,1990) suggested to ignore the unbalance and compute a single $\mathbf{S}_B$ using the estimator $c.$ of the average group size. Hence we get

$$\widehat{\mathbf{\Sigma}_W + c.\,\mathbf{\Sigma}_B} := \mathbf{S}_B\,.$$

The scaling factor c. for the given data is

```
x$c
```

```
## [1] 13.04835
```

which is smaller then the average group size

```
mean(table(AKS_PE$Gruppe))
```

```
## [1] 14.57576
```

due to the fact that the data contains a few groups with rather few observations.

Estimation algorithm

Step 1: The level one model

Check if one or three factors at level 1.

specify models, one factor und three factor model

```
onefactor <- 'f1 =~ Autonomy_PE_1 + Relatedness_PE_1 + Competence_PE_1 + Autonomy_PE_2 + Rel
atedness_PE_2 + Competence_PE_2 + Autonomy_PE_3 + Relatedness_PE_3 + Competence_PE_3 + Auton
omy_PE_4  + Competence_PE_4';
threefactor <- 'f1 =~ Autonomy_PE_1 + Autonomy_PE_2 + Autonomy_PE_3 + Autonomy_PE_4;
f2 =~ Relatedness_PE_1 + Relatedness_PE_2 + Relatedness_PE_3 ;
f3 =~ Competence_PE_1 + Competence_PE_2 + Competence_PE_3 + Competence_PE_4'
```

- fit models using the pooled within group variance matrix as input; output saved into an object called results1
- first argument is model object containing model definition
- second argument is source and type of data to be analyzed
- third argument is effective number of observations

```
# fit models using the pooled within group variance matrix as input; output saved into an ob
ject called results1
# first argument is model object containing model definition
# second argument is source and type of data to be analyzed
# third argument is effective number of observations

models$mod_one_fac <- cfa(onefactor, sample.cov = x$pw.cov, sample.nobs = x$n - x$G)
models$mod_three_fac <- cfa(threefactor, sample.cov = x$pw.cov, sample.nobs = x$n - x$G)
```

Providing measures of model fit and factor loadings in standardized form

```r
# Providing measures of model fit and factor loadings in standardized form
sapply(models[1:2], function(X) summary(X, fit.measures = T, standardized = T))
```

```
## lavaan 0.6-3 ended normally after 36 iterations
##
##   Optimization method                           NLMINB
##   Number of free parameters                         22
##
##   Number of observations                           400
##
##   Estimator                                         ML
##   Model Fit Test Statistic                     650.536
##   Degrees of freedom                                44
##   P-value (Chi-square)                           0.000
##
## Model test baseline model:
##
##   Minimum Function Test Statistic             1878.559
##   Degrees of freedom                                55
##   P-value                                        0.000
##
## User model versus baseline model:
##
##   Comparative Fit Index (CFI)                    0.667
##   Tucker-Lewis Index (TLI)                       0.584
##
## Loglikelihood and Information Criteria:
##
##   Loglikelihood user model (H0)              -5748.719
##   Loglikelihood unrestricted model (H1)      -5423.451
##
##   Number of free parameters                         22
##   Akaike (AIC)                               11541.438
##   Bayesian (BIC)                             11629.251
##   Sample-size adjusted Bayesian (BIC)        11559.443
##
## Root Mean Square Error of Approximation:
##
##   RMSEA                                          0.186
##   90 Percent Confidence Interval          0.173  0.198
##   P-value RMSEA <= 0.05                          0.000
##
## Standardized Root Mean Square Residual:
##
##   SRMR                                           0.117
##
## Parameter Estimates:
##
##   Information                                 Expected
##   Information saturated (h1) model          Structured
##   Standard Errors                             Standard
##
## Latent Variables:
##                    Estimate  Std.Err  z-value  P(>|z|)   Std.lv  Std.all
##   f1 =~
##     Autonomy_PE_1     1.000                               0.345    0.385
##     Relatdnss_PE_1    1.183    0.207    5.704    0.000    0.409    0.408
##     Competenc_PE_1    2.187    0.296    7.397    0.000    0.755    0.789
##     Autonomy_PE_2     1.538    0.241    6.395    0.000    0.531    0.513
##     Relatdnss_PE_2    1.394    0.233    5.987    0.000    0.482    0.446
##     Competenc_PE_2    2.444    0.334    7.320    0.000    0.844    0.756
##     Autonomy_PE_3     1.946    0.289    6.738    0.000    0.672    0.583
##     Relatdnss_PE_3    1.403    0.238    5.901    0.000    0.484    0.434
```

```
##     Competenc_PE_3    2.010    0.281    7.164    0.000    0.694    0.699
##     Autonomy_PE_4     1.850    0.266    6.950    0.000    0.639    0.635
##     Competenc_PE_4    1.998    0.278    7.182    0.000    0.690    0.705
##
## Variances:
##                    Estimate  Std.Err  z-value  P(>|z|)   Std.lv  Std.all
##    .Autonomy_PE_1     0.684    0.050   13.805    0.000    0.684    0.852
##    .Relatdnss_PE_1    0.838    0.061   13.756    0.000    0.838    0.834
##    .Competenc_PE_1    0.347    0.032   10.919    0.000    0.347    0.378
##    .Autonomy_PE_2     0.790    0.059   13.450    0.000    0.790    0.737
##    .Relatdnss_PE_2    0.931    0.068   13.660    0.000    0.931    0.801
##    .Competenc_PE_2    0.534    0.046   11.526    0.000    0.534    0.428
##    .Autonomy_PE_3     0.879    0.067   13.144    0.000    0.879    0.661
##    .Relatdnss_PE_3    1.010    0.074   13.692    0.000    1.010    0.811
##    .Competenc_PE_3    0.504    0.041   12.274    0.000    0.504    0.511
##    .Autonomy_PE_4     0.603    0.047   12.825    0.000    0.603    0.597
##    .Competenc_PE_4    0.481    0.039   12.207    0.000    0.481    0.502
##     f1                0.119    0.032    3.767    0.000    1.000    1.000
##
## lavaan 0.6-3 ended normally after 33 iterations
##
##   Optimization method                           NLMINB
##   Number of free parameters                         25
##
##   Number of observations                           400
##
##   Estimator                                         ML
##   Model Fit Test Statistic                     124.159
##   Degrees of freedom                                41
##   P-value (Chi-square)                           0.000
##
## Model test baseline model:
##
##   Minimum Function Test Statistic             1878.559
##   Degrees of freedom                                55
##   P-value                                        0.000
##
## User model versus baseline model:
##
##   Comparative Fit Index (CFI)                    0.954
##   Tucker-Lewis Index (TLI)                       0.939
##
## Loglikelihood and Information Criteria:
##
##   Loglikelihood user model (H0)              -5485.531
##   Loglikelihood unrestricted model (H1)      -5423.451
##
##   Number of free parameters                         25
##   Akaike (AIC)                               11021.062
##   Bayesian (BIC)                             11120.848
##   Sample-size adjusted Bayesian (BIC)        11041.522
##
## Root Mean Square Error of Approximation:
##
##   RMSEA                                          0.071
##   90 Percent Confidence Interval          0.057  0.086
##   P-value RMSEA <= 0.05                          0.008
##
## Standardized Root Mean Square Residual:
##
```

```
##   SRMR                                           0.054
##
## Parameter Estimates:
##
##   Information                                 Expected
##   Information saturated (h1) model          Structured
##   Standard Errors                             Standard
##
## Latent Variables:
##                    Estimate  Std.Err  z-value  P(>|z|)   Std.lv  Std.all
##   f1 =~
##     Autonomy_PE_1     1.000                               0.482    0.538
##     Autonomy_PE_2     1.545    0.157    9.842    0.000    0.745    0.720
##     Autonomy_PE_3     1.514    0.165    9.155    0.000    0.730    0.633
##     Autonomy_PE_4     1.815    0.174   10.446    0.000    0.876    0.871
##   f2 =~
##     Relatdnss_PE_1    1.000                               0.589    0.587
##     Relatdnss_PE_2    1.642    0.148   11.120    0.000    0.966    0.896
##     Relatdnss_PE_3    1.466    0.130   11.258    0.000    0.863    0.773
##   f3 =~
##     Competenc_PE_1    1.000                               0.811    0.847
##     Competenc_PE_2    1.102    0.063   17.612    0.000    0.894    0.801
##     Competenc_PE_3    0.829    0.058   14.299    0.000    0.672    0.677
##     Competenc_PE_4    0.922    0.055   16.662    0.000    0.748    0.765
##
## Covariances:
##                    Estimate  Std.Err  z-value  P(>|z|)   Std.lv  Std.all
##   f1 ~~
##     f2                0.104    0.021    4.924    0.000    0.366    0.366
##     f3                0.233    0.033    7.057    0.000    0.595    0.595
##   f2 ~~
##     f3                0.199    0.034    5.923    0.000    0.416    0.416
##
## Variances:
##                    Estimate  Std.Err  z-value  P(>|z|)   Std.lv  Std.all
##    .Autonomy_PE_1     0.570    0.044   13.090    0.000    0.570    0.710
##    .Autonomy_PE_2     0.517    0.047   11.079    0.000    0.517    0.482
##    .Autonomy_PE_3     0.797    0.065   12.352    0.000    0.797    0.599
##    .Autonomy_PE_4     0.244    0.040    6.091    0.000    0.244    0.242
##    .Relatdnss_PE_1    0.658    0.052   12.736    0.000    0.658    0.655
##    .Relatdnss_PE_2    0.229    0.057    4.049    0.000    0.229    0.197
##    .Relatdnss_PE_3    0.500    0.056    8.879    0.000    0.500    0.402
##    .Competenc_PE_1    0.259    0.029    8.850    0.000    0.259    0.283
##    .Competenc_PE_2    0.446    0.043   10.376    0.000    0.446    0.358
##    .Competenc_PE_3    0.534    0.043   12.416    0.000    0.534    0.541
##    .Competenc_PE_4    0.397    0.035   11.213    0.000    0.397    0.415
##     f1                0.233    0.043    5.383    0.000    1.000    1.000
##     f2                0.346    0.058    5.955    0.000    1.000    1.000
##     f3                0.658    0.066    9.928    0.000    1.000    1.000
```

```
## $mod_one_fac
## NULL
##
## $mod_three_fac
## NULL
```

Fit measures

Hox: Simulation research shows that all these indices still depend on sample size and estimation method (e.g., ML or GLS), with the CFI and the TLI/NNFI showing the best overall performance (Chou & Bentler, 1995; Kaplan, 1995). If the

model fits perfectly, these fit indices should have the value ***1****. Usually, a value of at least* ***.90*** *is required to accept a model, while a value of at least* ***.95*** *is required to judge the model fit as good. However, these are just rules of thumb. A different approach to model fit is to accept that models are only approximations, and that a perfect fit may be too much to ask for. Instead, the problem is to assess how well a given model approximates the true model. This view led to the development of an index called RMSEA, for root mean square error of approximation (Browne & Cudeck, 1992). If the approximation is good, the RMSEA should be small. Typically, an RMSEA of less than .08 is required (Kline, 2004), and an RMSEA of less than .05 is required to judge the model fit as good. Statistical tests or confidence intervals can be computed to test if the RMSEA is significantly larger than this lower bound. Given the many possible goodness-of-fit indices, the customary advice is to assess fit by inspecting several fit indices that derive from different principles.* ***Therefore, for the confirmatory factor model for the family data, I have reported the chi-square test, and the fit indices CFI and RMSEA.***

Path diagramm

```
semPlot::semPaths(models$mod_three_fac,"std",edge.label.cex=0.5, curvePivot = TRUE)
```

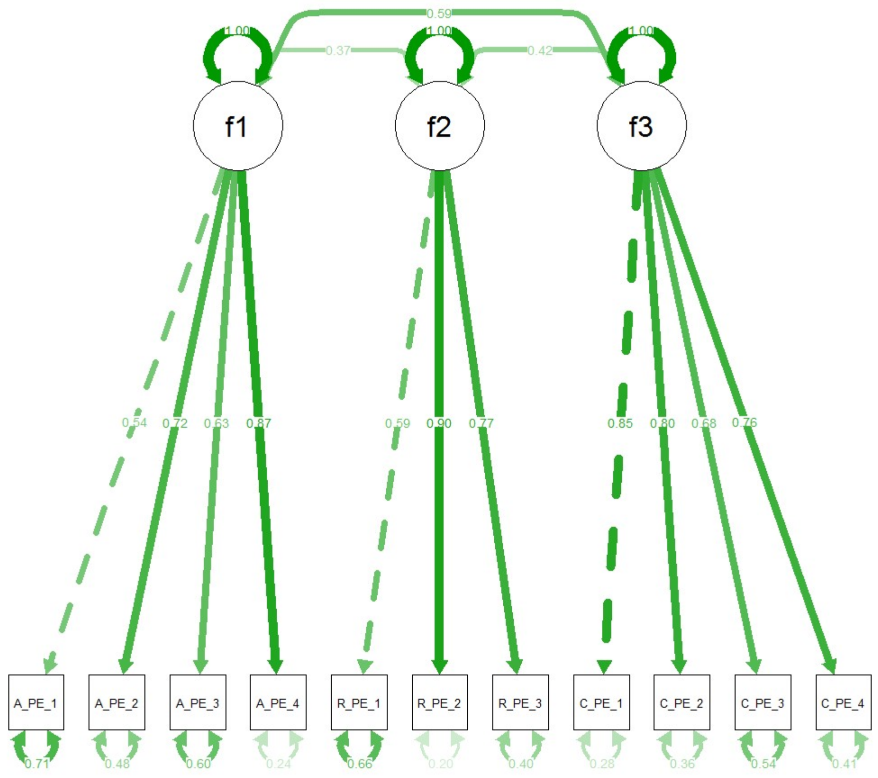

`threefactor` model fits better than `onefactor` model!

If an adequate fit is not found, there is little point in proceeding and researchers should revisit their theory behind their CFA (Huang, 2017).

Step 2: The null model

Assumption to be checked: 2 levels, 3 factors each, no variance between levels regarding factor loadings, variances and covariances

Store multiple input covariances (PW and B) and sample sizes for each in a list object

```
combined.cov <- list(within = x$pw.cov, between = x$b.cov)
combined.n <- list(within = x$n - x$G, between = x$G)
```

Specify model imposing equality constraints for within and between models (in this case for both levels)

```
nullmodel <- '
f1 =~ Autonomy_PE_1 + c(a,a)*Autonomy_PE_2 + c(b,b)*Autonomy_PE_3 + c(c,c)*Autonomy_PE_4
f2 =~ Relatedness_PE_1 + c(d,d)*Relatedness_PE_2 + c(e,e)*Relatedness_PE_3
f3 =~ Competence_PE_1 + c(g,g)*Competence_PE_2 + c(h,h)*Competence_PE_3 + c(i,i)*Competence_
PE_4
Autonomy_PE_1 ~~ c(j,j)*Autonomy_PE_1
Autonomy_PE_2 ~~ c(k,k)*Autonomy_PE_2
Autonomy_PE_3 ~~ c(l,l)*Autonomy_PE_3
Autonomy_PE_4 ~~ c(m,m)*Autonomy_PE_4
Relatedness_PE_1 ~~ c(n,n)*Relatedness_PE_1
Relatedness_PE_2 ~~ c(o,o)*Relatedness_PE_2
Relatedness_PE_3 ~~ c(p,p)*Relatedness_PE_3
Competence_PE_1 ~~ c(r,r)*Competence_PE_1
Competence_PE_2 ~~ c(s,s)*Competence_PE_2
Competence_PE_3 ~~ c(t,t)*Competence_PE_3
Competence_PE_4 ~~ c(u,u)*Competence_PE_4
f1 ~~ c(v,v)*f1
f2 ~~ c(w,w)*f2
f3 ~~ c(x,x)*f3
f1 ~~ c(y,y)*f2
f1 ~~ c(z,z)*f3
f2 ~~ c(aa,aa)*f3
'
# Fit null model
models$nullmodel_fit <- cfa(nullmodel, sample.cov = combined.cov, sample.nobs = combined.n)

# viewing summary statistics (in standardisierter Form)
fitMeasures(models$nullmodel_fit)
```

```
##                npar                fmin               chisq
##              25.000               0.316             273.515
##                  df              pvalue      baseline.chisq
##             107.000               0.000            2130.224
##         baseline.df     baseline.pvalue                 cfi
##             110.000               0.000               0.918
##                 tli                nnfi                 rfi
##               0.915               0.915               0.868
##                 nfi                pnfi                 ifi
##               0.872               0.848               0.918
##                 rni                logl   unrestricted.logl
##               0.918           -6004.124           -5867.367
##                 aic                 bic              ntotal
##           12058.249           12160.017             433.000
##                bic2               rmsea      rmsea.ci.lower
##           12080.681               0.085               0.072
##      rmsea.ci.upper        rmsea.pvalue                 rmr
##               0.097               0.000               0.116
##          rmr_nomean                srmr         srmr_bentler
##               0.116               0.091               0.091
## srmr_bentler_nomean                crmr         crmr_nomean
##               0.091               0.069               0.069
##          srmr_mplus   srmr_mplus_nomean               cn_05
##               0.081               0.081             210.197
##               cn_01                 gfi                agfi
##             228.870               0.910               0.889
##                pgfi                 mfi
##               0.738               0.825
```

Fits indicate there might be a variance between levels. At this stage Multilevel validation is justified.

Step 3: The independence model

Assumption to be checked: 2 levels, 3 factors each, no variance between levels regarding factor loadings, variances and covariances and there are latent factors at level 2, every manifest variable is composed by pooled-within and between variance.

Here, we use the scaling factor c, which is calculated by mcfa.input().

```
x$c
```

```
## [1] 13.04835
```

```
x$sqc
```

```
## [1] 3.612251
```

Now, we start to estimate the between variance of the model by estimation of 11 new group-level “factors”."

Every group-level factor has a specific loading of

```
x$sqc
```

```
## [1] 3.612251
```

on its manifest variable (Autonomy_PE_1 bis Competence_PE_4).

Autonomy_PE_1b etc. is the name of the level 2 factor and the 0 as first argument in brackets, denies the estimation of Autonomy_PE_1b at the first level (within level).

This model is called independence model, because by using “orthogonal = T” we deny that the new latent group level factors covary with each other.

```
independence <- '
f1 =~ Autonomy_PE_1 + c(a,a)*Autonomy_PE_2 + c(b,b)*Autonomy_PE_3 + c(c,c)*Autonomy_PE_4
f2 =~ Relatedness_PE_1 + c(d,d)*Relatedness_PE_2 + c(e,e)*Relatedness_PE_3
f3 =~ Competence_PE_1 + c(g,g)*Competence_PE_2 + c(h,h)*Competence_PE_3 + c(i,i)*Competence_
PE_4
Autonomy_PE_1 ~~ c(j,j)*Autonomy_PE_1
Autonomy_PE_2 ~~ c(k,k)*Autonomy_PE_2
Autonomy_PE_3 ~~ c(l,l)*Autonomy_PE_3
Autonomy_PE_4 ~~ c(m,m)*Autonomy_PE_4
Relatedness_PE_1 ~~ c(n,n)*Relatedness_PE_1
Relatedness_PE_2 ~~ c(o,o)*Relatedness_PE_2
Relatedness_PE_3 ~~ c(p,p)*Relatedness_PE_3
Competence_PE_1 ~~ c(r,r)*Competence_PE_1
Competence_PE_2 ~~ c(s,s)*Competence_PE_2
Competence_PE_3 ~~ c(t,t)*Competence_PE_3
Competence_PE_4 ~~ c(u,u)*Competence_PE_4
f1 ~~ c(v,v)*f1
f2 ~~ c(w,w)*f2
f3 ~~ c(x,x)*f3
f1 ~~ c(y,y)*f2
f1 ~~ c(z,z)*f3
f2 ~~ c(aa,aa)*f3
Autonomy_PE_1b =~ c(0,x$sqc)*Autonomy_PE_1
Autonomy_PE_1b ~~ c(0,NA)*Autonomy_PE_1b
Autonomy_PE_2b =~ c(0,x$sqc)*Autonomy_PE_2
Autonomy_PE_2b ~~ c(0,NA)*Autonomy_PE_2b
Autonomy_PE_3b =~ c(0,x$sqc)*Autonomy_PE_3
Autonomy_PE_3b ~~ c(0,NA)*Autonomy_PE_3b
Autonomy_PE_4b =~ c(0,x$sqc)*Autonomy_PE_4
Autonomy_PE_4b ~~ c(0,NA)*Autonomy_PE_4b
Relatedness_PE_1b =~ c(0,x$sqc)*Relatedness_PE_1
Relatedness_PE_1b ~~ c(0,NA)*Relatedness_PE_1b
Relatedness_PE_2b =~ c(0,x$scq)*Relatedness_PE_2
Relatedness_PE_2b ~~ c(0,NA)*Relatedness_PE_2b
Relatedness_PE_3b =~ c(0,x$scq)*Relatedness_PE_3
Relatedness_PE_3b ~~ c(0,NA)*Relatedness_PE_3b
Competence_PE_1b =~ c(0,x$scq)*Competence_PE_1
Competence_PE_1b ~~ c(0,NA)*Competence_PE_1b
Competence_PE_2b =~ c(0,x$scq)*Competence_PE_2
Competence_PE_2b ~~ c(0,NA)*Competence_PE_2b
Competence_PE_3b =~ c(0,x$scq)*Competence_PE_3
Competence_PE_3b ~~ c(0,NA)*Competence_PE_3b
Competence_PE_4b =~ c(0,x$scq)*Competence_PE_4
Competence_PE_4b ~~ c(0,NA)*Competence_PE_4b
'
# Fit measures
models$mod_ind_fit <- cfa(independence, sample.cov = combined.cov, sample.nobs = combined.n,
orthogonal = T)
```

```
## Warning in lav_object_post_check(object): lavaan WARNING: some estimated lv
## variances are negative
```

```
# Summary
fitMeasures(models$mod_ind_fit)
```

```
##                npar                fmin               chisq 
##              35.000               0.282             244.328 
##                  df              pvalue      baseline.chisq 
##              97.000               0.000            2130.224 
##         baseline.df     baseline.pvalue                 cfi 
##             110.000               0.000               0.927 
##                 tli                nnfi                 rfi 
##               0.917               0.917               0.870 
##                 nfi                pnfi                 ifi 
##               0.885               0.781               0.928 
##                 rni                logl   unrestricted.logl 
##               0.927           -5989.530           -5867.367 
##                 aic                 bic              ntotal 
##           12049.061           12191.537             433.000 
##                bic2               rmsea      rmsea.ci.lower 
##           12080.466               0.084               0.071 
##      rmsea.ci.upper        rmsea.pvalue                 rmr 
##               0.097               0.000               0.157 
##          rmr_nomean                srmr        srmr_bentler 
##               0.157               0.136               0.136 
## srmr_bentler_nomean                crmr         crmr_nomean 
##               0.136               0.077               0.077 
##          srmr_mplus   srmr_mplus_nomean               cn_05 
##               0.099               0.099             215.419 
##               cn_01                 gfi                agfi 
##             235.479               0.915               0.884 
##                pgfi                 mfi 
##               0.672               0.844
```

If the independence model fit well, the conclusion would be that there is substantial group-level variance but there is no substantively interesting structural model (Hox 2002). If the independence model did not fit well, that suggests that there is some kind of structural model at the group level that should be modeled (Huang, 2017).

Step 4: The saturated model

Now, we allow the introduced latent group level factors to covary.

Since we have 11 latent variables we need to estimate 55 covariances. (k*(k-1))/2, k = number of variables.

Keep the argument orthogonal = T.

```
saturated <- '
f1 =~ Autonomy_PE_1 + c(a,a)*Autonomy_PE_2 + c(b,b)*Autonomy_PE_3 + c(c,c)*Autonomy_PE_4
f2 =~ Relatedness_PE_1 + c(d,d)*Relatedness_PE_2 + c(e,e)*Relatedness_PE_3
f3 =~ Competence_PE_1 + c(g,g)*Competence_PE_2 + c(h,h)*Competence_PE_3 + c(i,i)*Competence_
PE_4
Autonomy_PE_1 ~~ c(j,j)*Autonomy_PE_1
Autonomy_PE_2 ~~ c(k,k)*Autonomy_PE_2
Autonomy_PE_3 ~~ c(l,l)*Autonomy_PE_3
Autonomy_PE_4 ~~ c(m,m)*Autonomy_PE_4
Relatedness_PE_1 ~~ c(n,n)*Relatedness_PE_1
Relatedness_PE_2 ~~ c(o,o)*Relatedness_PE_2
Relatedness_PE_3 ~~ c(p,p)*Relatedness_PE_3
Competence_PE_1 ~~ c(r,r)*Competence_PE_1
Competence_PE_2 ~~ c(s,s)*Competence_PE_2
Competence_PE_3 ~~ c(t,t)*Competence_PE_3
Competence_PE_4 ~~ c(u,u)*Competence_PE_4
f1 ~~ c(v,v)*f1
f2 ~~ c(w,w)*f2
f3 ~~ c(x,x)*f3
f1 ~~ c(y,y)*f2
f1 ~~ c(z,z)*f3
f2 ~~ c(aa,aa)*f3
Autonomy_PE_1b =~ c(0,x$scq)*Autonomy_PE_1
Autonomy_PE_1b ~~ c(0,NA)*Autonomy_PE_1b
Autonomy_PE_2b =~ c(0,x$scq)*Autonomy_PE_2
Autonomy_PE_2b ~~ c(0,NA)*Autonomy_PE_2b
Autonomy_PE_3b =~ c(0,x$scq)*Autonomy_PE_3
Autonomy_PE_3b ~~ c(0,NA)*Autonomy_PE_3b
Autonomy_PE_4b =~ c(0,x$scq)*Autonomy_PE_4
Autonomy_PE_4b ~~ c(0,NA)*Autonomy_PE_4b
Relatedness_PE_1b =~ c(0,x$scq)*Relatedness_PE_1
Relatedness_PE_1b ~~ c(0,NA)*Relatedness_PE_1b
Relatedness_PE_2b =~ c(0,x$scq)*Relatedness_PE_2
Relatedness_PE_2b ~~ c(0,NA)*Relatedness_PE_2b
Relatedness_PE_3b =~ c(0,x$sqc)*Relatedness_PE_3
Relatedness_PE_3b ~~ c(0,NA)*Relatedness_PE_3b
Competence_PE_1b =~ c(0,x$scq)*Competence_PE_1
Competence_PE_1b ~~ c(0,NA)*Competence_PE_1b
Competence_PE_2b =~ c(0,x$scq)*Competence_PE_2
Competence_PE_2b ~~ c(0,NA)*Competence_PE_2b
Competence_PE_3b =~ c(0,x$scq)*Competence_PE_3
Competence_PE_3b ~~ c(0,NA)*Competence_PE_3b
Competence_PE_4b =~ c(0,x$scq)*Competence_PE_4
Competence_PE_4b ~~ c(0,NA)*Competence_PE_4b

Autonomy_PE_1b ~~ c(0,NA)*Autonomy_PE_2b + c(0,NA)*Autonomy_PE_3b + c(0,NA)*Autonomy_PE_4b +
c(0,NA)*Relatedness_PE_1b + c(0,NA)*Relatedness_PE_2b + c(0,NA)*Relatedness_PE_3b +  + c(0,N
A)*Competence_PE_1b + c(0,NA)*Competence_PE_2b + c(0,NA)*Competence_PE_3b + c(0,NA)*Competen
ce_PE_4b

Autonomy_PE_2b ~~ c(0,NA)*Autonomy_PE_3b + c(0,NA)*Autonomy_PE_4b + c(0,NA)*Relatedness_PE_1
b + c(0,NA)*Relatedness_PE_2b + c(0,NA)*Relatedness_PE_3b + c(0,NA)*Competence_PE_1b + c(0,N
A)*Competence_PE_2b + c(0,NA)*Competence_PE_3b + c(0,NA)*Competence_PE_4b

Autonomy_PE_3b ~~ c(0,NA)*Autonomy_PE_4b + c(0,NA)*Relatedness_PE_1b + c(0,NA)*Relatedness_P
E_2b + c(0,NA)*Relatedness_PE_3b + c(0,NA)*Competence_PE_1b + c(0,NA)*Competence_PE_2b + c
(0,NA)*Competence_PE_3b + c(0,NA)*Competence_PE_4b

Autonomy_PE_4b ~~ c(0,NA)*Relatedness_PE_1b + c(0,NA)*Relatedness_PE_2b + c(0,NA)*Relatednes
s_PE_3b + c(0,NA)*Competence_PE_1b + c(0,NA)*Competence_PE_2b + c(0,NA)*Competence_PE_3b + c
```

```
(0,NA)*Competence_PE_4b

Relatedness_PE_1b ~~ c(0,NA)*Relatedness_PE_2b + c(0,NA)*Relatedness_PE_3b + c(0,NA)*Compete
nce_PE_1b + c(0,NA)*Competence_PE_2b + c(0,NA)*Competence_PE_3b + c(0,NA)*Competence_PE_4b

Relatedness_PE_2b ~~ c(0,NA)*Relatedness_PE_3b + c(0,NA)*Competence_PE_1b + c(0,NA)*Competen
ce_PE_2b + c(0,NA)*Competence_PE_3b + c(0,NA)*Competence_PE_4b

Relatedness_PE_3b ~~ c(0,NA)*Competence_PE_1b + c(0,NA)*Competence_PE_2b + c(0,NA)*Competenc
e_PE_3b + c(0,NA)*Competence_PE_4b

Competence_PE_1b ~~ c(0,NA)*Competence_PE_2b + c(0,NA)*Competence_PE_3b + c(0,NA)*Competence
_PE_4b

Competence_PE_2b ~~ c(0,NA)*Competence_PE_3b + c(0,NA)*Competence_PE_4b

Competence_PE_3b ~~ c(0,NA)*Competence_PE_4b
'
# Fit saturated model
models$mod_sat_fit <- cfa(saturated, sample.cov = combined.cov, sample.nobs = combined.n, or
thogonal = T)
```

```
## Warning in lav_object_post_check(object): lavaan WARNING: some estimated lv
## variances are negative
```

```
fitMeasures(models$mod_sat_fit)
```

```
##                npar                fmin               chisq 
##              90.000               0.164             142.171 
##                  df              pvalue      baseline.chisq 
##              42.000               0.000            2130.224 
##         baseline.df     baseline.pvalue                 cfi 
##             110.000               0.000               0.950 
##                 tli                nnfi                 rfi 
##               0.870               0.870               0.825 
##                 nfi                pnfi                 ifi 
##               0.933               0.356               0.952 
##                 rni                logl   unrestricted.logl 
##               0.950           -5938.452           -5867.367 
##                 aic                 bic              ntotal 
##           12056.904           12423.271             433.000 
##                bic2               rmsea      rmsea.ci.lower 
##           12137.661               0.105               0.086 
##      rmsea.ci.upper        rmsea.pvalue                 rmr 
##               0.124               0.000               0.107 
##          rmr_nomean                srmr        srmr_bentler 
##               0.107               0.104               0.104 
## srmr_bentler_nomean                crmr         crmr_nomean 
##               0.104               0.062               0.062 
##          srmr_mplus   srmr_mplus_nomean               cn_05 
##               0.076               0.076             178.024 
##               cn_01                 gfi                agfi 
##             202.640               0.943               0.822 
##                pgfi                 mfi 
##               0.300               0.891
```

Mixed fit indices indicate an error or that the fit in step 1 was bad (based on Huang). The bad fit could be taken as indicator that there might be no interesting structural model at level 2. Since we can assure that no mistakes were made we continue with step 5.

Step 5

3x1-Modell

Here, we specify our hypothesized model. Initially, we model a latent overall factor. Secondly, we model the theory-based three factor model at level 2.

Therefore, we remove the 55 freely estimated covariances by the assumed level 2 model.

```
mod_3x1 <- '
f1 =~ Autonomy_PE_1 + c(a,a)*Autonomy_PE_2 + c(b,b)*Autonomy_PE_3 + c(c,c)*Autonomy_PE_4
f2 =~ Relatedness_PE_1 + c(d,d)*Relatedness_PE_2 + c(e,e)*Relatedness_PE_3
f3 =~ Competence_PE_1 + c(g,g)*Competence_PE_2 + c(h,h)*Competence_PE_3 + c(i,i)*Competence_
PE_4
Autonomy_PE_1 ~~ c(j,j)*Autonomy_PE_1
Autonomy_PE_2 ~~ c(k,k)*Autonomy_PE_2
Autonomy_PE_3 ~~ c(l,l)*Autonomy_PE_3
Autonomy_PE_4 ~~ c(m,m)*Autonomy_PE_4
Relatedness_PE_1 ~~ c(n,n)*Relatedness_PE_1
Relatedness_PE_2 ~~ c(o,o)*Relatedness_PE_2
Relatedness_PE_3 ~~ c(p,p)*Relatedness_PE_3
Competence_PE_1 ~~ c(r,r)*Competence_PE_1
Competence_PE_2 ~~ c(s,s)*Competence_PE_2
Competence_PE_3 ~~ c(t,t)*Competence_PE_3
Competence_PE_4 ~~ c(u,u)*Competence_PE_4
f1 ~~ c(v,v)*f1
f2 ~~ c(w,w)*f2
f3 ~~ c(x,x)*f3
f1 ~~ c(y,y)*f2
f1 ~~ c(z,z)*f3
f2 ~~ c(aa,aa)*f3
Autonomy_PE_1b =~ c(0,x$sqc)*Autonomy_PE_1
Autonomy_PE_1b ~~ c(0,NA)*Autonomy_PE_1b
Autonomy_PE_2b =~ c(0,x$sqc)*Autonomy_PE_2
Autonomy_PE_2b ~~ c(0,NA)*Autonomy_PE_2b
Autonomy_PE_3b =~ c(0,x$sqc)*Autonomy_PE_3
Autonomy_PE_3b ~~ c(0,NA)*Autonomy_PE_3b
Autonomy_PE_4b =~ c(0,x$sqc)*Autonomy_PE_4
Autonomy_PE_4b ~~ c(0,NA)*Autonomy_PE_4b
Relatedness_PE_1b =~ c(0,x$sqc)*Relatedness_PE_1
Relatedness_PE_1b ~~ c(0,NA)*Relatedness_PE_1b
Relatedness_PE_2b =~ c(0,x$sqc)*Relatedness_PE_2
Relatedness_PE_2b ~~ c(0,NA)*Relatedness_PE_2b
Relatedness_PE_3b =~ c(0,x$sqc)*Relatedness_PE_3
Relatedness_PE_3b ~~ c(0,NA)*Relatedness_PE_3b
Competence_PE_1b =~ c(0,x$sqc)*Competence_PE_1
Competence_PE_1b ~~ c(0,NA)*Competence_PE_1b
Competence_PE_2b =~ c(0,x$sqc)*Competence_PE_2
Competence_PE_2b ~~ c(0,NA)*Competence_PE_2b
Competence_PE_3b =~ c(0,x$sqc)*Competence_PE_3
Competence_PE_3b ~~ c(0,NA)*Competence_PE_3b
Competence_PE_4b =~ c(0,x$sqc)*Competence_PE_4
Competence_PE_4b ~~ c(0,NA)*Competence_PE_4b
f1b =~ c(0,1)*Autonomy_PE_1b + c(0,NA)*Autonomy_PE_2b + c(0,NA)*Autonomy_PE_3b + c(0,NA)*Aut
onomy_PE_4b + c(0,NA)*Relatedness_PE_1b + c(0,NA)*Relatedness_PE_2b + c(0,NA)*Relatedness_PE
_3b + c(0,NA)*Competence_PE_1b + c(0,NA)*Competence_PE_2b + c(0,NA)*Competence_PE_3b + c(0,N
A)*Competence_PE_4b
f1b ~~ c(0,NA)*f1b + c(0,0)*f1 + c(0,0)*f2 + c(0,0)*f3
'
# Fit hypothesized model
models$mod_3x1_fit <- cfa(mod_3x1, sample.cov = combined.cov, sample.nobs = combined.n, orth
ogonal = T)
```

```
## Warning in lav_object_post_check(object): lavaan WARNING: some estimated lv
## variances are negative
```

```
summary(models$mod_3x1_fit, fit.measures = T, standardized = T)
```

```
## lavaan 0.6-3 ended normally after 108 iterations
##
##   Optimization method                           NLMINB
##   Number of free parameters                         70
##   Number of equality constraints                    24
##
##   Number of observations per group
##   within                                           400
##   between                                           33
##
##   Estimator                                         ML
##   Model Fit Test Statistic                     209.600
##   Degrees of freedom                                86
##   P-value (Chi-square)                           0.000
##
## Chi-square for each group:
##
##   within                                       143.049
##   between                                       66.551
##
## Model test baseline model:
##
##   Minimum Function Test Statistic             2130.224
##   Degrees of freedom                               110
##   P-value                                        0.000
##
## User model versus baseline model:
##
##   Comparative Fit Index (CFI)                    0.939
##   Tucker-Lewis Index (TLI)                       0.922
##
## Loglikelihood and Information Criteria:
##
##   Loglikelihood user model (H0)              -5972.167
##   Loglikelihood unrestricted model (H1)      -5867.367
##
##   Number of free parameters                         46
##   Akaike (AIC)                               12036.333
##   Bayesian (BIC)                             12223.587
##   Sample-size adjusted Bayesian (BIC)        12077.609
##
## Root Mean Square Error of Approximation:
##
##   RMSEA                                          0.081
##   90 Percent Confidence Interval          0.068  0.096
##   P-value RMSEA <= 0.05                          0.000
##
## Standardized Root Mean Square Residual:
##
##   SRMR                                           0.119
```

```
## Warning in sqrt(ETA2): NaNs wurden erzeugt
```

```
## Warning in sqrt(ETA2): NaNs wurden erzeugt

## Warning in sqrt(ETA2): NaNs wurden erzeugt
```

```
##
## Parameter Estimates:
##
##   Information                                 Expected
##   Information saturated (h1) model          Structured
##   Standard Errors                             Standard
##
##
## Group 1 [within]:
##
## Latent Variables:
##                        Estimate  Std.Err  z-value  P(>|z|)   Std.lv
##   f1 =~
##     Atnmy_PE_1            1.000                               0.505
##     Atnmy_PE_2 (a)        1.540    0.147   10.507    0.000    0.778
##     Atnmy_PE_3 (b)        1.551    0.157    9.885    0.000    0.783
##     Atnmy_PE_4 (c)        1.805    0.162   11.173    0.000    0.911
##   f2 =~
##     Rltdn_PE_1            1.000                               0.608
##     Rltdn_PE_2 (d)        1.625    0.135   12.025    0.000    0.987
##     Rltdn_PE_3 (e)        1.385    0.115   12.079    0.000    0.841
##   f3 =~
##     Cmptn_PE_1            1.000                               1.000
##     Cmptn_PE_2 (g)        0.999    0.042   23.832    0.000    0.999
##     Cmptn_PE_3 (h)        0.753    0.041   18.138    0.000    0.753
##     Cmptn_PE_4 (i)        0.840    0.038   22.079    0.000    0.840
##   Autonomy_PE_1b =~
##     Atnmy_PE_1 (x)        1.000                               0.000
##   Autonomy_PE_2b =~
##     Atnmy_PE_2 (x)        1.000                               0.000
##   Autonomy_PE_3b =~
##     Atnmy_PE_3 (x)        1.000                               0.000
##   Autonomy_PE_4b =~
##     Atnmy_PE_4 (x)        1.000                               0.000
##   Relatedness_PE_1b =~
##     Rltdn_PE_1 (x)        1.000                               0.000
##   Relatedness_PE_2b =~
##     Rltdn_PE_2 (x)        1.000                               0.000
##   Relatedness_PE_3b =~
##     Rltdn_PE_3 (x)        1.000                               0.000
##   Competence_PE_1b =~
##     Cmptn_PE_1 (x)        1.000                               0.000
##   Competence_PE_2b =~
##     Cmptn_PE_2 (x)        1.000                               0.000
##   Competence_PE_3b =~
##     Cmptn_PE_3 (x)        1.000                               0.000
##   Competence_PE_4b =~
##     Cmptn_PE_4 (x)        1.000                               0.000
##   f1b =~
##     Atnmy_PE_1            0.000                                 NaN
##     Atnmy_PE_2            0.000                                 NaN
##     Atnmy_PE_3            0.000                                 NaN
##     Atnmy_PE_4            0.000                                 NaN
##     Rltdn_PE_1            0.000                                 NaN
##     Rltdn_PE_2            0.000                                 NaN
##     Rltdn_PE_3            0.000                                 NaN
##     Cmptn_PE_1            0.000                                 NaN
##     Cmptn_PE_2            0.000                                 NaN
##     Cmptn_PE_3            0.000                                 NaN
##     Cmptn_PE_4            0.000                                 NaN
```

```
##   Std.all
##
##     0.556
##     0.734
##     0.661
##     0.877
##
##     0.600
##     0.905
##     0.760
##
##     0.899
##     0.829
##     0.715
##     0.798
##
##     0.000
##
##     0.000
##
##     0.000
##
##     0.000
##
##     0.000
##
##     0.000
##
##     0.000
##
##     0.000
##
##     0.000
##
##     0.000
##
##     0.000
##
##       NaN
##       NaN
##       NaN
##       NaN
##       NaN
##       NaN
##       NaN
##       NaN
##       NaN
##       NaN
##       NaN
##
## Covariances:
##                    Estimate  Std.Err  z-value  P(>|z|)   Std.lv  Std.all
##   f1 ~~
##     f2          (y)    0.123    0.022    5.520    0.000    0.401    0.401
##     f3          (z)    0.324    0.035    9.162    0.000    0.642    0.642
##   f2 ~~
##     f3         (aa)    0.276    0.036    7.581    0.000    0.454    0.454
##   f1 ~~
##     f1b                0.000                                 NaN      NaN
##   f2 ~~
```

```
##     f1b               0.000                               NaN      NaN
##   f3 ~~
##     f1b               0.000                               NaN      NaN
##
## Variances:
##                    Estimate  Std.Err  z-value  P(>|z|)   Std.lv  Std.all
##    .Atnmy_PE_1 (j)    0.570    0.043   13.103    0.000    0.570    0.691
##    .Atnmy_PE_2 (k)    0.517    0.046   11.208    0.000    0.517    0.461
##    .Atnmy_PE_3 (l)    0.791    0.064   12.288    0.000    0.791    0.563
##    .Atnmy_PE_4 (m)    0.250    0.039    6.447    0.000    0.250    0.232
##    .Rltdn_PE_1 (n)    0.657    0.052   12.708    0.000    0.657    0.640
##    .Rltdn_PE_2 (o)    0.214    0.055    3.922    0.000    0.214    0.180
##    .Rltdn_PE_3 (p)    0.518    0.054    9.680    0.000    0.518    0.422
##    .Cmptn_PE_1 (r)    0.238    0.029    8.060    0.000    0.238    0.192
##    .Cmptn_PE_2 (s)    0.456    0.042   10.798    0.000    0.456    0.313
##    .Cmptn_PE_3 (t)    0.542    0.043   12.609    0.000    0.542    0.489
##    .Cmptn_PE_4 (u)    0.402    0.035   11.505    0.000    0.402    0.363
##     f1         (v)    0.255    0.045    5.700    0.000    1.000    1.000
##     f2         (w)    0.369    0.058    6.395    0.000    1.000    1.000
##     f3         (x)    1.000                               1.000    1.000
##     Atnmy_PE_1        0.000                               NaN      NaN
##     Atnmy_PE_2        0.000                               NaN      NaN
##     Atnmy_PE_3        0.000                               NaN      NaN
##     Atnmy_PE_4        0.000                               NaN      NaN
##     Rltdn_PE_1        0.000                               NaN      NaN
##     Rltdn_PE_2        0.000                               NaN      NaN
##     Rltdn_PE_3        0.000                               NaN      NaN
##     Cmptn_PE_1        0.000                               NaN      NaN
##     Cmptn_PE_2        0.000                               NaN      NaN
##     Cmptn_PE_3        0.000                               NaN      NaN
##     Cmptn_PE_4        0.000                               NaN      NaN
##     f1b               0.000                               NaN      NaN
##
##
## Group 2 [between]:
##
## Latent Variables:
##                        Estimate  Std.Err  z-value  P(>|z|)   Std.lv
##   f1 =~
##     Atn_PE_1              1.000                               0.505
##     Atn_PE_2   (a)        1.540    0.147   10.507    0.000    0.778
##     Atn_PE_3   (b)        1.551    0.157    9.885    0.000    0.783
##     Atn_PE_4   (c)        1.805    0.162   11.173    0.000    0.911
##   f2 =~
##     Rlt_PE_1              1.000                               0.608
##     Rlt_PE_2   (d)        1.625    0.135   12.025    0.000    0.987
##     Rlt_PE_3   (e)        1.385    0.115   12.079    0.000    0.841
##   f3 =~
##     Cmp_PE_1              1.000                               1.000
##     Cmp_PE_2   (g)        0.999    0.042   23.832    0.000    0.999
##     Cmp_PE_3   (h)        0.753    0.041   18.138    0.000    0.753
##     Cmp_PE_4   (i)        0.840    0.038   22.079    0.000    0.840
##   Autonomy_PE_1b =~
##     Atn_PE_1 (sqc)        1.000                               1.694
##   Autonomy_PE_2b =~
##     Atn_PE_2 (sqc)        1.000                               0.935
##   Autonomy_PE_3b =~
##     Atn_PE_3 (sqc)        1.000                               0.924
##   Autonomy_PE_4b =~
##     Atn_PE_4 (sqc)        1.000                               1.254
```

```
##    Relatedness_PE_1b =~
##      Rlt_PE_1 (sqc)        1.000                                   0.206
##    Relatedness_PE_2b =~
##      Rlt_PE_2 (sqc)        1.000                                     NaN
##    Relatedness_PE_3b =~
##      Rlt_PE_3 (sqc)        1.000                                     NaN
##    Competence_PE_1b =~
##      Cmp_PE_1 (sqc)        1.000                                   0.332
##    Competence_PE_2b =~
##      Cmp_PE_2 (sqc)        1.000                                   0.267
##    Competence_PE_3b =~
##      Cmp_PE_3 (sqc)        1.000                                   0.532
##    Competence_PE_4b =~
##      Cmp_PE_4 (sqc)        1.000                                   0.314
##    f1b =~
##      Atn_PE_1              1.000                                   0.842
##      Atn_PE_2              0.724    0.172    4.221    0.000    1.104
##      Atn_PE_3              0.726    0.186    3.907    0.000    1.120
##      Atn_PE_4              0.835    0.197    4.246    0.000    0.950
##      Rlt_PE_1              0.096    0.147    0.655    0.513    0.666
##      Rlt_PE_2             -0.008    0.151   -0.051    0.960      NaN
##      Rlt_PE_3              0.038    0.145    0.265    0.791      NaN
##      Cmp_PE_1              0.070    0.166    0.423    0.672    0.301
##      Cmp_PE_2              0.174    0.172    1.014    0.311    0.928
##      Cmp_PE_3              0.263    0.162    1.625    0.104    0.706
##      Cmp_PE_4              0.019    0.159    0.118    0.906    0.085
##    Std.all
##
##      0.263
##      0.550
##      0.521
##      0.559
##
##      0.588
##      0.953
##      0.831
##
##      0.861
##      0.809
##      0.638
##      0.765
##
##      0.881
##
##      0.662
##
##      0.615
##
##      0.770
##
##      0.200
##
##        NaN
##
##        NaN
##
##      0.286
##
##      0.216
##
```

```
##     0.451
##
##     0.286
##
##     0.842
##     1.104
##     1.120
##     0.950
##     0.666
##       NaN
##       NaN
##     0.301
##     0.928
##     0.706
##     0.085
##
## Covariances:
##                    Estimate  Std.Err  z-value  P(>|z|)   Std.lv  Std.all
##   f1 ~~
##     f2          (y)    0.123    0.022    5.520    0.000    0.401    0.401
##     f3          (z)    0.324    0.035    9.162    0.000    0.642    0.642
##   f2 ~~
##     f3         (aa)    0.276    0.036    7.581    0.000    0.454    0.454
##   f1 ~~
##     f1b                0.000                               0.000    0.000
##   f2 ~~
##     f1b                0.000                               0.000    0.000
##   f3 ~~
##     f1b                0.000                               0.000    0.000
##
## Variances:
##                    Estimate  Std.Err  z-value  P(>|z|)   Std.lv  Std.all
##    .Atnmy_PE_1 (j)    0.570    0.043   13.103    0.000    0.570    0.154
##    .Atnmy_PE_2 (k)    0.517    0.046   11.208    0.000    0.517    0.259
##    .Atnmy_PE_3 (l)    0.791    0.064   12.288    0.000    0.791    0.350
##    .Atnmy_PE_4 (m)    0.250    0.039    6.447    0.000    0.250    0.094
##    .Rltdn_PE_1 (n)    0.657    0.052   12.708    0.000    0.657    0.615
##    .Rltdn_PE_2 (o)    0.214    0.055    3.922    0.000    0.214    0.200
##    .Rltdn_PE_3 (p)    0.518    0.054    9.680    0.000    0.518    0.506
##    .Cmptn_PE_1 (r)    0.238    0.029    8.060    0.000    0.238    0.176
##    .Cmptn_PE_2 (s)    0.456    0.042   10.798    0.000    0.456    0.299
##    .Cmptn_PE_3 (t)    0.542    0.043   12.609    0.000    0.542    0.390
##    .Cmptn_PE_4 (u)    0.402    0.035   11.505    0.000    0.402    0.334
##     f1          (v)    0.255    0.045    5.700    0.000    1.000    1.000
##     f2          (w)    0.369    0.058    6.395    0.000    1.000    1.000
##     f3          (x)    1.000                               1.000    1.000
##     Atnmy_PE_1         0.837    0.462    1.814    0.070    0.292    0.292
##     Atnmy_PE_2        -0.191    0.131   -1.462    0.144   -0.219   -0.219
##     Atnmy_PE_3        -0.217    0.186   -1.164    0.244   -0.254   -0.254
##     Atnmy_PE_4         0.154    0.163    0.947    0.343    0.098    0.098
##     Rltdn_PE_1         0.024    0.184    0.129    0.897    0.557    0.557
##     Rltdn_PE_2        -0.117    0.106   -1.102    0.270      NaN      NaN
##     Rltdn_PE_3        -0.205    0.115   -1.784    0.074      NaN      NaN
##     Cmptn_PE_1         0.100    0.135    0.744    0.457    0.910    0.910
##     Cmptn_PE_2         0.010    0.158    0.063    0.950    0.139    0.139
##     Cmptn_PE_3         0.142    0.194    0.731    0.465    0.502    0.502
##     Cmptn_PE_4         0.098    0.156    0.627    0.530    0.993    0.993
##     f1b                2.032    0.923    2.202    0.028    1.000    1.000
```

Path diagramm

```
semPlot::semPaths(models$mod_3x1_fit,"std",edge.label.cex=0.5, curvePivot = TRUE)
```

```
## Warning in sqrt(ETA2): NaNs wurden erzeugt

## Warning in sqrt(ETA2): NaNs wurden erzeugt

## Warning in sqrt(ETA2): NaNs wurden erzeugt
```

```
## Warning in qgraph::qgraph(Edgelist, labels = nLab, bidirectional = Bidir, :
## Non-finite weights are omitted

## Warning in qgraph::qgraph(Edgelist, labels = nLab, bidirectional = Bidir, :
## Non-finite weights are omitted
```

1

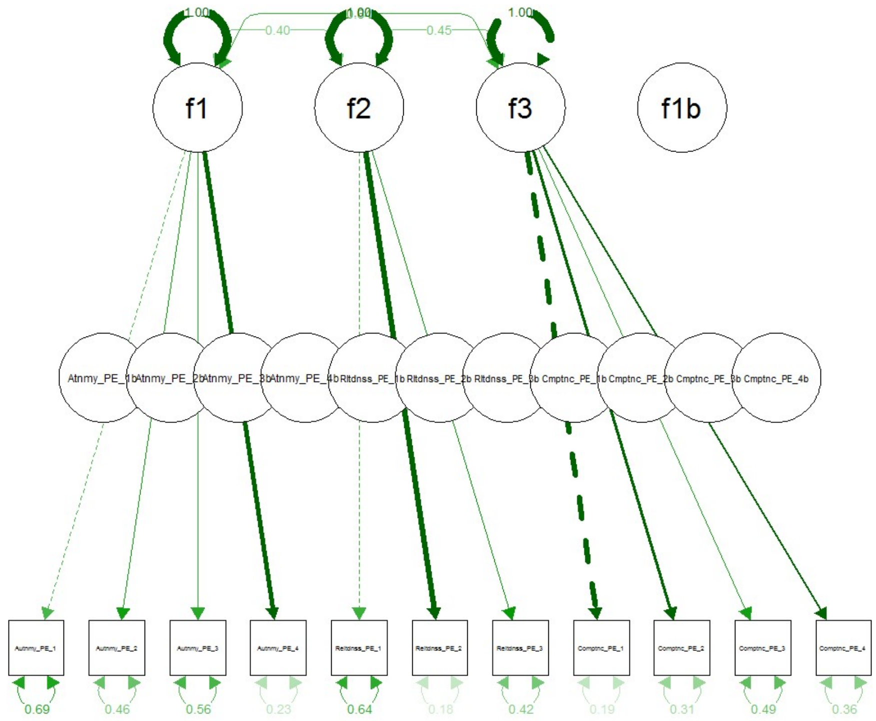

2

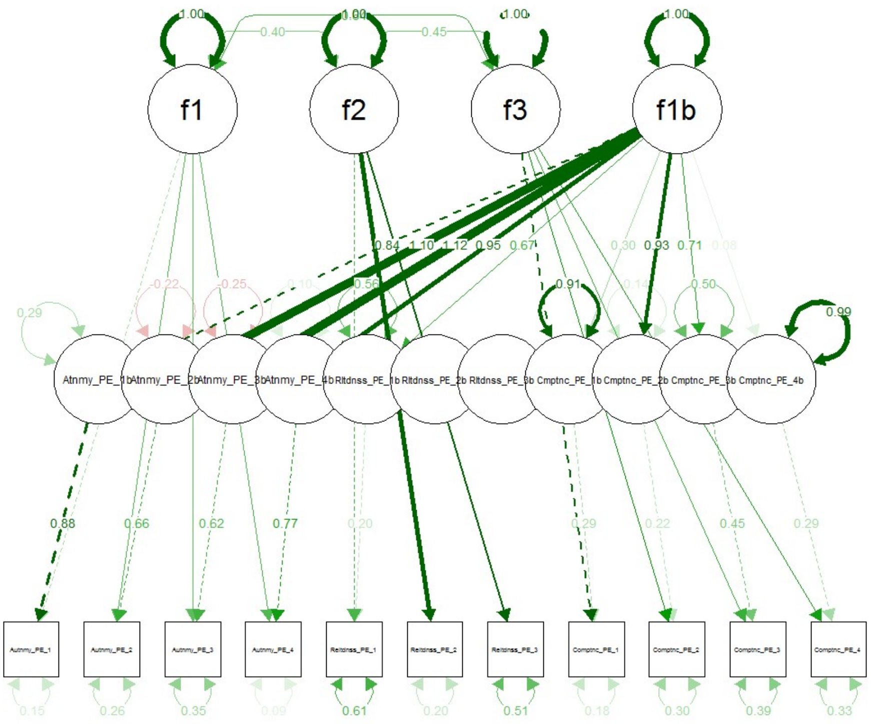

3x1, variances set to 0

Huang & Cornell, 2016: The presence of small negative residual variance estimates at the school level required residual variances to be fixed to zero for five items (see Table 1). Fixing residual variances at the group level to zero is often done in MLCFA and is recommended to allow the model to converge… **Model A**

```
mod_3x1_var <- '
f1 =~ Autonomy_PE_1 + c(a,a)*Autonomy_PE_2 + c(b,b)*Autonomy_PE_3 + c(c,c)*Autonomy_PE_4
f2 =~ Relatedness_PE_1 + c(d,d)*Relatedness_PE_2 + c(e,e)*Relatedness_PE_3
f3 =~ Competence_PE_1 + c(g,g)*Competence_PE_2 + c(h,h)*Competence_PE_3 + c(i,i)*Competence_
PE_4
Autonomy_PE_1 ~~ c(j,j)*Autonomy_PE_1
Autonomy_PE_2 ~~ c(NA,0)*Autonomy_PE_2
Autonomy_PE_3 ~~ c(NA,0)*Autonomy_PE_3
Autonomy_PE_4 ~~ c(m,m)*Autonomy_PE_4
Relatedness_PE_1 ~~ c(n,n)*Relatedness_PE_1
Relatedness_PE_2 ~~ c(NA,0)*Relatedness_PE_2
Relatedness_PE_3 ~~ c(NA,0)*Relatedness_PE_3
Competence_PE_1 ~~ c(r,r)*Competence_PE_1
Competence_PE_2 ~~ c(s,s)*Competence_PE_2
Competence_PE_3 ~~ c(t,t)*Competence_PE_3
Competence_PE_4 ~~ c(u,u)*Competence_PE_4
f1 ~~ c(v,v)*f1
f2 ~~ c(w,w)*f2
f3 ~~ c(x,x)*f3
f1 ~~ c(y,y)*f2
f1 ~~ c(z,z)*f3
f2 ~~ c(aa,aa)*f3
Autonomy_PE_1b =~ c(0,x$sqc)*Autonomy_PE_1
Autonomy_PE_1b ~~ c(0,NA)*Autonomy_PE_1b
Autonomy_PE_2b =~ c(0,x$sqc)*Autonomy_PE_2
Autonomy_PE_2b ~~ c(0,NA)*Autonomy_PE_2b
Autonomy_PE_3b =~ c(0,x$sqc)*Autonomy_PE_3
Autonomy_PE_3b ~~ c(0,NA)*Autonomy_PE_3b
Autonomy_PE_4b =~ c(0,x$sqc)*Autonomy_PE_4
Autonomy_PE_4b ~~ c(0,NA)*Autonomy_PE_4b
Relatedness_PE_1b =~ c(0,x$sqc)*Relatedness_PE_1
Relatedness_PE_1b ~~ c(0,NA)*Relatedness_PE_1b
Relatedness_PE_2b =~ c(0,x$sqc)*Relatedness_PE_2
Relatedness_PE_2b ~~ c(0,NA)*Relatedness_PE_2b
Relatedness_PE_3b =~ c(0,x$sqc)*Relatedness_PE_3
Relatedness_PE_3b ~~ c(0,NA)*Relatedness_PE_3b
Competence_PE_1b =~ c(0,x$sqc)*Competence_PE_1
Competence_PE_1b ~~ c(0,NA)*Competence_PE_1b
Competence_PE_2b =~ c(0,x$sqc)*Competence_PE_2
Competence_PE_2b ~~ c(0,NA)*Competence_PE_2b
Competence_PE_3b =~ c(0,x$sqc)*Competence_PE_3
Competence_PE_3b ~~ c(0,NA)*Competence_PE_3b
Competence_PE_4b =~ c(0,x$sqc)*Competence_PE_4
Competence_PE_4b ~~ c(0,NA)*Competence_PE_4b
f1b =~ c(0,1)*Autonomy_PE_1b + c(0,NA)*Autonomy_PE_2b + c(0,NA)*Autonomy_PE_3b + c(0,NA)*Aut
onomy_PE_4b + c(0,NA)*Relatedness_PE_1b + c(0,NA)*Relatedness_PE_2b + c(0,NA)*Relatedness_PE
_3b + c(0,NA)*Competence_PE_1b + c(0,NA)*Competence_PE_2b + c(0,NA)*Competence_PE_3b + c(0,N
A)*Competence_PE_4b
f1b ~~ c(0,NA)*f1b + c(0,0)*f1 + c(0,0)*f2 + c(0,0)*f3
'
# Fit hypothesized model
models$mod_3x1_var_fit <- cfa(mod_3x1_var, sample.cov = combined.cov, sample.nobs = combine
d.n, orthogonal = T)
summary(models$mod_3x1_var_fit, fit.measures = T, standardized = T)
```

```
## lavaan 0.6-3 ended normally after 111 iterations
##
##   Optimization method                           NLMINB
##   Number of free parameters                         66
##   Number of equality constraints                    20
##
##   Number of observations per group
##   within                                           400
##   between                                           33
##
##   Estimator                                         ML
##   Model Fit Test Statistic                     209.600
##   Degrees of freedom                                86
##   P-value (Chi-square)                           0.000
##
## Chi-square for each group:
##
##   within                                       143.049
##   between                                       66.551
##
## Model test baseline model:
##
##   Minimum Function Test Statistic             2130.224
##   Degrees of freedom                               110
##   P-value                                        0.000
##
## User model versus baseline model:
##
##   Comparative Fit Index (CFI)                    0.939
##   Tucker-Lewis Index (TLI)                       0.922
##
## Loglikelihood and Information Criteria:
##
##   Loglikelihood user model (H0)              -5972.167
##   Loglikelihood unrestricted model (H1)      -5867.367
##
##   Number of free parameters                         46
##   Akaike (AIC)                               12036.333
##   Bayesian (BIC)                             12223.587
##   Sample-size adjusted Bayesian (BIC)        12077.609
##
## Root Mean Square Error of Approximation:
##
##   RMSEA                                          0.081
##   90 Percent Confidence Interval          0.068  0.096
##   P-value RMSEA <= 0.05                          0.000
##
## Standardized Root Mean Square Residual:
##
##   SRMR                                           0.119
##
## Parameter Estimates:
##
##   Information                                 Expected
##   Information saturated (h1) model          Structured
##   Standard Errors                             Standard
##
##
## Group 1 [within]:
##
```

```
## Latent Variables:
##                        Estimate  Std.Err  z-value  P(>|z|)   Std.lv
##   f1 =~
##     Atnmy_PE_1            1.000                               0.505
##     Atnmy_PE_2 (a)        1.540    0.147   10.507    0.000    0.778
##     Atnmy_PE_3 (b)        1.551    0.157    9.885    0.000    0.783
##     Atnmy_PE_4 (c)        1.805    0.162   11.173    0.000    0.911
##   f2 =~
##     Rltdn_PE_1            1.000                               0.608
##     Rltdn_PE_2 (d)        1.625    0.135   12.025    0.000    0.987
##     Rltdn_PE_3 (e)        1.385    0.115   12.079    0.000    0.841
##   f3 =~
##     Cmptn_PE_1            1.000                               1.000
##     Cmptn_PE_2 (g)        0.999    0.042   23.832    0.000    0.999
##     Cmptn_PE_3 (h)        0.753    0.041   18.138    0.000    0.753
##     Cmptn_PE_4 (i)        0.840    0.038   22.079    0.000    0.840
##   Autonomy_PE_1b =~
##     Atnmy_PE_1 (x)        1.000                               0.000
##   Autonomy_PE_2b =~
##     Atnmy_PE_2 (x)        1.000                               0.000
##   Autonomy_PE_3b =~
##     Atnmy_PE_3 (x)        1.000                               0.000
##   Autonomy_PE_4b =~
##     Atnmy_PE_4 (x)        1.000                               0.000
##   Relatedness_PE_1b =~
##     Rltdn_PE_1 (x)        1.000                               0.000
##   Relatedness_PE_2b =~
##     Rltdn_PE_2 (x)        1.000                               0.000
##   Relatedness_PE_3b =~
##     Rltdn_PE_3 (x)        1.000                               0.000
##   Competence_PE_1b =~
##     Cmptn_PE_1 (x)        1.000                               0.000
##   Competence_PE_2b =~
##     Cmptn_PE_2 (x)        1.000                               0.000
##   Competence_PE_3b =~
##     Cmptn_PE_3 (x)        1.000                               0.000
##   Competence_PE_4b =~
##     Cmptn_PE_4 (x)        1.000                               0.000
##   f1b =~
##     Atnmy_PE_1            0.000                                 NaN
##     Atnmy_PE_2            0.000                                 NaN
##     Atnmy_PE_3            0.000                                 NaN
##     Atnmy_PE_4            0.000                                 NaN
##     Rltdn_PE_1            0.000                                 NaN
##     Rltdn_PE_2            0.000                                 NaN
##     Rltdn_PE_3            0.000                                 NaN
##     Cmptn_PE_1            0.000                                 NaN
##     Cmptn_PE_2            0.000                                 NaN
##     Cmptn_PE_3            0.000                                 NaN
##     Cmptn_PE_4            0.000                                 NaN
##   Std.all
##
##     0.556
##     0.734
##     0.661
##     0.877
##
##     0.600
##     0.905
##     0.760
```

```
##
##     0.899
##     0.829
##     0.715
##     0.798
##
##     0.000
##
##     0.000
##
##     0.000
##
##     0.000
##
##     0.000
##
##     0.000
##
##     0.000
##
##     0.000
##
##     0.000
##
##     0.000
##
##     0.000
##
##       NaN
##       NaN
##       NaN
##       NaN
##       NaN
##       NaN
##       NaN
##       NaN
##       NaN
##       NaN
##       NaN
##
## Covariances:
##                    Estimate  Std.Err  z-value  P(>|z|)   Std.lv  Std.all
##   f1 ~~
##     f2          (y)    0.123    0.022    5.520    0.000    0.401    0.401
##     f3          (z)    0.324    0.035    9.162    0.000    0.642    0.642
##   f2 ~~
##     f3         (aa)    0.276    0.036    7.581    0.000    0.454    0.454
##   f1 ~~
##     f1b                0.000                                 NaN      NaN
##   f2 ~~
##     f1b                0.000                                 NaN      NaN
##   f3 ~~
##     f1b                0.000                                 NaN      NaN
##
## Variances:
##                    Estimate  Std.Err  z-value  P(>|z|)   Std.lv  Std.all
##    .Atnmy_PE_1 (j)    0.570    0.043   13.103    0.000    0.570    0.691
##    .Atnmy_PE_2        0.517    0.046   11.208    0.000    0.517    0.461
##    .Atnmy_PE_3        0.791    0.064   12.288    0.000    0.791    0.563
##    .Atnmy_PE_4 (m)    0.250    0.039    6.447    0.000    0.250    0.232
```

```
##    .Rltdn_PE_1 (n)    0.657    0.052   12.708    0.000    0.657    0.640
##    .Rltdn_PE_2        0.214    0.055    3.922    0.000    0.214    0.180
##    .Rltdn_PE_3        0.518    0.054    9.680    0.000    0.518    0.423
##    .Cmptn_PE_1 (r)    0.238    0.029    8.060    0.000    0.238    0.192
##    .Cmptn_PE_2 (s)    0.456    0.042   10.798    0.000    0.456    0.313
##    .Cmptn_PE_3 (t)    0.542    0.043   12.609    0.000    0.542    0.489
##    .Cmptn_PE_4 (u)    0.402    0.035   11.505    0.000    0.402    0.363
##     f1         (v)    0.255    0.045    5.700    0.000    1.000    1.000
##     f2         (w)    0.369    0.058    6.395    0.000    1.000    1.000
##     f3         (x)    1.000                               1.000    1.000
##     Atnmy_PE_1        0.000                                 NaN      NaN
##     Atnmy_PE_2        0.000                                 NaN      NaN
##     Atnmy_PE_3        0.000                                 NaN      NaN
##     Atnmy_PE_4        0.000                                 NaN      NaN
##     Rltdn_PE_1        0.000                                 NaN      NaN
##     Rltdn_PE_2        0.000                                 NaN      NaN
##     Rltdn_PE_3        0.000                                 NaN      NaN
##     Cmptn_PE_1        0.000                                 NaN      NaN
##     Cmptn_PE_2        0.000                                 NaN      NaN
##     Cmptn_PE_3        0.000                                 NaN      NaN
##     Cmptn_PE_4        0.000                                 NaN      NaN
##     f1b               0.000                                 NaN      NaN
##
##
## Group 2 [between]:
##
## Latent Variables:
##                        Estimate  Std.Err  z-value  P(>|z|)   Std.lv
##   f1 =~
##     Atn_PE_1              1.000                               0.505
##     Atn_PE_2   (a)        1.540    0.147   10.507    0.000    0.778
##     Atn_PE_3   (b)        1.551    0.157    9.885    0.000    0.783
##     Atn_PE_4   (c)        1.805    0.162   11.173    0.000    0.911
##   f2 =~
##     Rlt_PE_1              1.000                               0.608
##     Rlt_PE_2   (d)        1.625    0.135   12.025    0.000    0.987
##     Rlt_PE_3   (e)        1.385    0.115   12.079    0.000    0.841
##   f3 =~
##     Cmp_PE_1              1.000                               1.000
##     Cmp_PE_2   (g)        0.999    0.042   23.832    0.000    0.999
##     Cmp_PE_3   (h)        0.753    0.041   18.138    0.000    0.753
##     Cmp_PE_4   (i)        0.840    0.038   22.079    0.000    0.840
##   Autonomy_PE_1b =~
##     Atn_PE_1 (sqc)        1.000                               1.694
##   Autonomy_PE_2b =~
##     Atn_PE_2 (sqc)        1.000                               1.179
##   Autonomy_PE_3b =~
##     Atn_PE_3 (sqc)        1.000                               1.283
##   Autonomy_PE_4b =~
##     Atn_PE_4 (sqc)        1.000                               1.254
##   Relatedness_PE_1b =~
##     Rlt_PE_1 (sqc)        1.000                               0.206
##   Relatedness_PE_2b =~
##     Rlt_PE_2 (sqc)        1.000                               0.313
##   Relatedness_PE_3b =~
##     Rlt_PE_3 (sqc)        1.000                               0.562
##   Competence_PE_1b =~
##     Cmp_PE_1 (sqc)        1.000                               0.332
##   Competence_PE_2b =~
##     Cmp_PE_2 (sqc)        1.000                               0.267
```

```
##   Competence_PE_3b =~
##     Cmp_PE_3 (sqc)        1.000                                   0.532
##   Competence_PE_4b =~
##     Cmp_PE_4 (sqc)        1.000                                   0.314
##   f1b =~
##     Atn_PE_1              1.000                                   0.842
##     Atn_PE_2              0.724    0.172    4.221    0.000    0.875
##     Atn_PE_3              0.726    0.186    3.907    0.000    0.807
##     Atn_PE_4              0.835    0.197    4.246    0.000    0.950
##     Rlt_PE_1              0.096    0.147    0.655    0.513    0.666
##     Rlt_PE_2             -0.008    0.151   -0.051    0.960   -0.035
##     Rlt_PE_3              0.038    0.145    0.265    0.791    0.098
##     Cmp_PE_1              0.070    0.166    0.423    0.672    0.301
##     Cmp_PE_2              0.174    0.172    1.014    0.311    0.928
##     Cmp_PE_3              0.263    0.162    1.625    0.104    0.706
##     Cmp_PE_4              0.019    0.159    0.118    0.906    0.085
##   Std.all
##
##     0.263
##     0.550
##     0.521
##     0.559
##
##     0.588
##     0.953
##     0.831
##
##     0.861
##     0.809
##     0.638
##     0.765
##
##     0.881
##
##     0.835
##
##     0.854
##
##     0.770
##
##     0.200
##
##     0.302
##
##     0.556
##
##     0.286
##
##     0.216
##
##     0.451
##
##     0.286
##
##     0.842
##     0.875
##     0.807
##     0.950
##     0.666
##    -0.035
```

```
##     0.098
##     0.301
##     0.928
##     0.706
##     0.085
##
## Covariances:
##                    Estimate  Std.Err  z-value  P(>|z|)   Std.lv  Std.all
##   f1 ~~
##     f2          (y)    0.123    0.022    5.520    0.000    0.401    0.401
##     f3          (z)    0.324    0.035    9.162    0.000    0.642    0.642
##   f2 ~~
##     f3         (aa)    0.276    0.036    7.581    0.000    0.454    0.454
##   f1 ~~
##     f1b                0.000                               0.000    0.000
##   f2 ~~
##     f1b                0.000                               0.000    0.000
##   f3 ~~
##     f1b                0.000                               0.000    0.000
##
## Variances:
##                    Estimate  Std.Err  z-value  P(>|z|)   Std.lv  Std.all
##    .Atnmy_PE_1 (j)    0.570    0.043   13.103    0.000    0.570    0.154
##    .Atnmy_PE_2        0.000                               0.000    0.000
##    .Atnmy_PE_3        0.000                               0.000    0.000
##    .Atnmy_PE_4 (m)    0.250    0.039    6.447    0.000    0.250    0.094
##    .Rltdn_PE_1 (n)    0.657    0.052   12.708    0.000    0.657    0.615
##    .Rltdn_PE_2        0.000                               0.000    0.000
##    .Rltdn_PE_3        0.000                               0.000    0.000
##    .Cmptn_PE_1 (r)    0.238    0.029    8.060    0.000    0.238    0.176
##    .Cmptn_PE_2 (s)    0.456    0.042   10.798    0.000    0.456    0.299
##    .Cmptn_PE_3 (t)    0.542    0.043   12.609    0.000    0.542    0.390
##    .Cmptn_PE_4 (u)    0.402    0.035   11.505    0.000    0.402    0.334
##     f1          (v)    0.255    0.045    5.700    0.000    1.000    1.000
##     f2          (w)    0.369    0.058    6.395    0.000    1.000    1.000
##     f3          (x)    1.000                               1.000    1.000
##     Atnmy_PE_1         0.837    0.462    1.814    0.070    0.292    0.292
##     Atnmy_PE_2         0.325    0.123    2.645    0.008    0.234    0.234
##     Atnmy_PE_3         0.574    0.175    3.276    0.001    0.349    0.349
##     Atnmy_PE_4         0.154    0.163    0.947    0.344    0.098    0.098
##     Rltdn_PE_1         0.024    0.184    0.129    0.897    0.557    0.557
##     Rltdn_PE_2         0.098    0.106    0.922    0.356    0.999    0.999
##     Rltdn_PE_3         0.313    0.108    2.889    0.004    0.990    0.990
##     Cmptn_PE_1         0.100    0.135    0.744    0.457    0.910    0.910
##     Cmptn_PE_2         0.010    0.158    0.063    0.950    0.139    0.139
##     Cmptn_PE_3         0.142    0.194    0.731    0.465    0.502    0.502
##     Cmptn_PE_4         0.098    0.156    0.627    0.530    0.993    0.993
##     f1b                2.032    0.923    2.202    0.028    1.000    1.000
```

see fit indices of model A in table 2

Path diagramm

```
semPlot::semPaths(models$mod_3x1_var_fit,"std",edge.label.cex=0.5, curvePivot = TRUE)
```

```
## Warning in qgraph::qgraph(Edgelist, labels = nLab, bidirectional = Bidir, :
## Non-finite weights are omitted
```

1

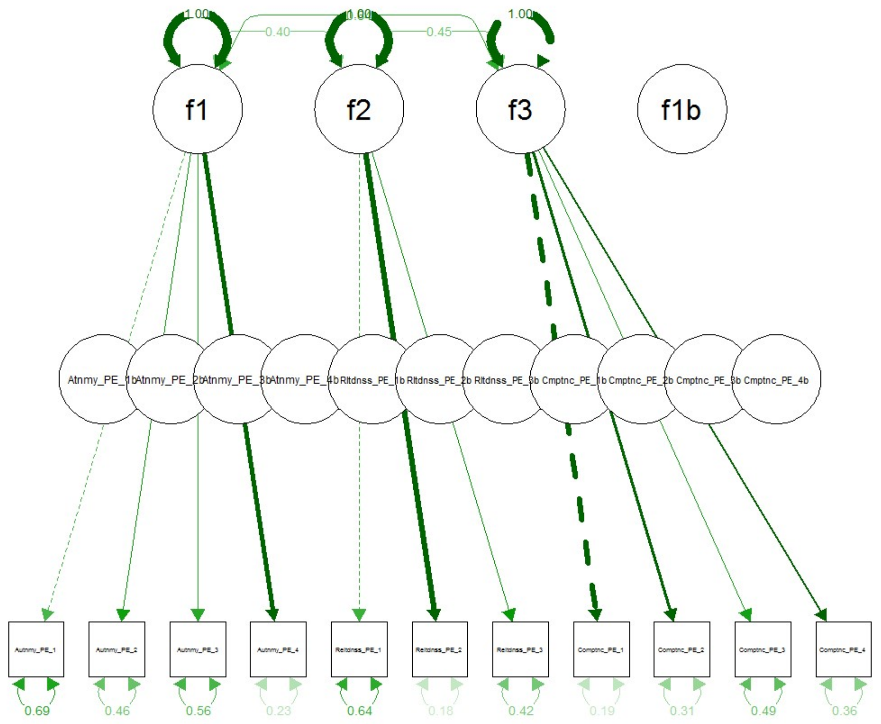

2

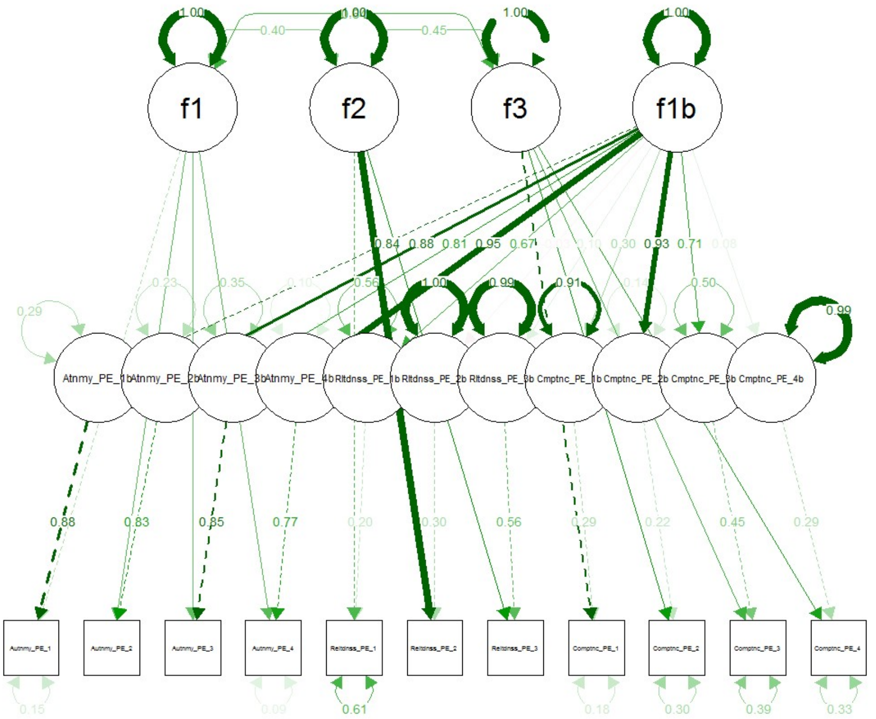

3x1, freely estimated loadings, variances and covariances

3x1-Modell, no equality constraints, freely estimated loadings, variances and covariances **model can’t be identified.**

3x1, freely estimated loadings and latent factor variances and covariances, manifest variances set equal

3x1, factor loadings and covariances freely estimated, variances set equal

```
mod_3x1_free_vareq <- '
f1 =~ Autonomy_PE_1 + Autonomy_PE_2 + Autonomy_PE_3 + Autonomy_PE_4
f2 =~ Relatedness_PE_1 + Relatedness_PE_2 + Relatedness_PE_3
f3 =~ Competence_PE_1 + Competence_PE_2 + Competence_PE_3 + Competence_PE_4
Autonomy_PE_1 ~~ c(j,j)*Autonomy_PE_1
Autonomy_PE_2 ~~ c(k,k)*Autonomy_PE_2
Autonomy_PE_3 ~~ c(l,l)*Autonomy_PE_3
Autonomy_PE_4 ~~ c(m,m)*Autonomy_PE_4
Relatedness_PE_1 ~~ c(n,n)*Relatedness_PE_1
Relatedness_PE_2 ~~ c(o,o)*Relatedness_PE_2
Relatedness_PE_3 ~~ c(p,p)*Relatedness_PE_3
Competence_PE_1 ~~ c(r,r)*Competence_PE_1
Competence_PE_2 ~~ c(s,s)*Competence_PE_2
Competence_PE_3 ~~ c(t,t)*Competence_PE_3
Competence_PE_4 ~~ c(u,u)*Competence_PE_4
f1 ~~ f1
f2 ~~ f2
f3 ~~ f3
f1 ~~ f2
f1 ~~ f3
f2 ~~ f3
Autonomy_PE_1b =~ c(0,x$sqc)*Autonomy_PE_1
Autonomy_PE_1b ~~ c(0,NA)*Autonomy_PE_1b
Autonomy_PE_2b =~ c(0,x$sqc)*Autonomy_PE_2
Autonomy_PE_2b ~~ c(0,NA)*Autonomy_PE_2b
Autonomy_PE_3b =~ c(0,x$sqc)*Autonomy_PE_3
Autonomy_PE_3b ~~ c(0,NA)*Autonomy_PE_3b
Autonomy_PE_4b =~ c(0,x$sqc)*Autonomy_PE_4
Autonomy_PE_4b ~~ c(0,NA)*Autonomy_PE_4b
Relatedness_PE_1b =~ c(0,x$sqc)*Relatedness_PE_1
Relatedness_PE_1b ~~ c(0,NA)*Relatedness_PE_1b
Relatedness_PE_2b =~ c(0,x$sqc)*Relatedness_PE_2
Relatedness_PE_2b ~~ c(0,NA)*Relatedness_PE_2b
Relatedness_PE_3b =~ c(0,x$sqc)*Relatedness_PE_3
Relatedness_PE_3b ~~ c(0,NA)*Relatedness_PE_3b
Competence_PE_1b =~ c(0,x$sqc)*Competence_PE_1
Competence_PE_1b ~~ c(0,NA)*Competence_PE_1b
Competence_PE_2b =~ c(0,x$sqc)*Competence_PE_2
Competence_PE_2b ~~ c(0,NA)*Competence_PE_2b
Competence_PE_3b =~ c(0,x$sqc)*Competence_PE_3
Competence_PE_3b ~~ c(0,NA)*Competence_PE_3b
Competence_PE_4b =~ c(0,x$sqc)*Competence_PE_4
Competence_PE_4b ~~ c(0,NA)*Competence_PE_4b
f1b =~ c(0,1)*Autonomy_PE_1b + c(0,NA)*Autonomy_PE_2b + c(0,NA)*Autonomy_PE_3b + c(0,NA)*Aut
onomy_PE_4b + c(0,NA)*Relatedness_PE_1b + c(0,NA)*Relatedness_PE_2b + c(0,NA)*Relatedness_PE
_3b +  c(0,NA)*Competence_PE_1b + c(0,NA)*Competence_PE_2b + c(0,NA)*Competence_PE_3b + c(0,
NA)*Competence_PE_4b
f1b ~~ c(0,NA)*f1b + c(0,0)*f1 + c(0,0)*f2 + c(0,0)*f3
'
# Fit model
models$mod_3x1_free_vareq_fit <- cfa(mod_3x1_free_vareq, sample.cov = combined.cov, sample.n
obs = combined.n, orthogonal = T)
```

```
## Warning in lav_object_post_check(object): lavaan WARNING: some estimated lv
## variances are negative
```

```
summary(models$mod_3x1_free_vareq_fit, fit.measures = T, standardized = T)
```

```
## lavaan 0.6-3 ended normally after 220 iterations
##
##   Optimization method                           NLMINB
##   Number of free parameters                         72
##   Number of equality constraints                    11
##
##   Number of observations per group
##   within                                           400
##   between                                           33
##
##   Estimator                                         ML
##   Model Fit Test Statistic                     157.964
##   Degrees of freedom                                71
##   P-value (Chi-square)                           0.000
##
## Chi-square for each group:
##
##   within                                       124.159
##   between                                       33.805
##
## Model test baseline model:
##
##   Minimum Function Test Statistic             2130.224
##   Degrees of freedom                               110
##   P-value                                        0.000
##
## User model versus baseline model:
##
##   Comparative Fit Index (CFI)                    0.957
##   Tucker-Lewis Index (TLI)                       0.933
##
## Loglikelihood and Information Criteria:
##
##   Loglikelihood user model (H0)              -5946.349
##   Loglikelihood unrestricted model (H1)      -5867.367
##
##   Number of free parameters                         61
##   Akaike (AIC)                               12014.698
##   Bayesian (BIC)                             12263.013
##   Sample-size adjusted Bayesian (BIC)        12069.432
##
## Root Mean Square Error of Approximation:
##
##   RMSEA                                          0.075
##   90 Percent Confidence Interval          0.059  0.091
##   P-value RMSEA <= 0.05                          0.005
##
## Standardized Root Mean Square Residual:
##
##   SRMR                                           0.055
```

```
## Warning in sqrt(ETA2): NaNs wurden erzeugt
```

```
## Warning in sqrt(ETA2): NaNs wurden erzeugt

## Warning in sqrt(ETA2): NaNs wurden erzeugt
```

```
##
## Parameter Estimates:
##
##   Information                                 Expected
##   Information saturated (h1) model          Structured
##   Standard Errors                             Standard
##
##
## Group 1 [within]:
##
## Latent Variables:
##                        Estimate  Std.Err  z-value  P(>|z|)   Std.lv
##   f1 =~
##     Atnmy_PE_1            1.000                               0.482
##     Atnmy_PE_2            1.545    0.157    9.842    0.000    0.745
##     Atnmy_PE_3            1.514    0.165    9.155    0.000    0.730
##     Atnmy_PE_4            1.815    0.174   10.446    0.000    0.876
##   f2 =~
##     Rltdn_PE_1            1.000                               0.589
##     Rltdn_PE_2            1.642    0.148   11.120    0.000    0.966
##     Rltdn_PE_3            1.466    0.130   11.258    0.000    0.863
##   f3 =~
##     Cmptn_PE_1            1.000                               0.811
##     Cmptn_PE_2            1.102    0.063   17.612    0.000    0.894
##     Cmptn_PE_3            0.829    0.058   14.299    0.000    0.672
##     Cmptn_PE_4            0.922    0.055   16.662    0.000    0.748
##   Autonomy_PE_1b =~
##     Atnmy_PE_1 (x)        1.000                               0.000
##   Autonomy_PE_2b =~
##     Atnmy_PE_2 (x)        1.000                               0.000
##   Autonomy_PE_3b =~
##     Atnmy_PE_3 (x)        1.000                               0.000
##   Autonomy_PE_4b =~
##     Atnmy_PE_4 (x)        1.000                               0.000
##   Relatedness_PE_1b =~
##     Rltdn_PE_1 (x)        1.000                               0.000
##   Relatedness_PE_2b =~
##     Rltdn_PE_2 (x)        1.000                               0.000
##   Relatedness_PE_3b =~
##     Rltdn_PE_3 (x)        1.000                               0.000
##   Competence_PE_1b =~
##     Cmptn_PE_1 (x)        1.000                               0.000
##   Competence_PE_2b =~
##     Cmptn_PE_2 (x)        1.000                               0.000
##   Competence_PE_3b =~
##     Cmptn_PE_3 (x)        1.000                               0.000
##   Competence_PE_4b =~
##     Cmptn_PE_4 (x)        1.000                               0.000
##   f1b =~
##     Atnmy_PE_1            0.000                                 NaN
##     Atnmy_PE_2            0.000                                 NaN
##     Atnmy_PE_3            0.000                                 NaN
##     Atnmy_PE_4            0.000                                 NaN
##     Rltdn_PE_1            0.000                                 NaN
##     Rltdn_PE_2            0.000                                 NaN
##     Rltdn_PE_3            0.000                                 NaN
##     Cmptn_PE_1            0.000                                 NaN
##     Cmptn_PE_2            0.000                                 NaN
##     Cmptn_PE_3            0.000                                 NaN
##     Cmptn_PE_4            0.000                                 NaN
```

```
##   Std.all
##
##     0.538
##     0.720
##     0.633
##     0.871
##
##     0.587
##     0.896
##     0.773
##
##     0.847
##     0.801
##     0.677
##     0.765
##
##     0.000
##
##     0.000
##
##     0.000
##
##     0.000
##
##     0.000
##
##     0.000
##
##     0.000
##
##     0.000
##
##     0.000
##
##     0.000
##
##     0.000
##
##       NaN
##       NaN
##       NaN
##       NaN
##       NaN
##       NaN
##       NaN
##       NaN
##       NaN
##       NaN
##       NaN
##
## Covariances:
##                    Estimate  Std.Err  z-value  P(>|z|)   Std.lv  Std.all
##   f1 ~~
##     f2                0.104    0.021    4.924    0.000    0.366    0.366
##     f3                0.233    0.033    7.057    0.000    0.595    0.595
##   f2 ~~
##     f3                0.199    0.034    5.923    0.000    0.416    0.416
##   f1 ~~
##     f1b               0.000                                 NaN      NaN
##   f2 ~~
```

```
##      f1b                0.000                               NaN      NaN
##    f3 ~~
##      f1b                0.000                               NaN      NaN
##
## Variances:
##                    Estimate  Std.Err  z-value  P(>|z|)   Std.lv  Std.all
##     .Atnmy_PE_1 (j)    0.570    0.044   13.090    0.000    0.570    0.710
##     .Atnmy_PE_2 (k)    0.517    0.047   11.079    0.000    0.517    0.482
##     .Atnmy_PE_3 (l)    0.797    0.065   12.352    0.000    0.797    0.599
##     .Atnmy_PE_4 (m)    0.244    0.040    6.091    0.000    0.244    0.242
##     .Rltdn_PE_1 (n)    0.658    0.052   12.736    0.000    0.658    0.655
##     .Rltdn_PE_2 (o)    0.229    0.057    4.049    0.000    0.229    0.197
##     .Rltdn_PE_3 (p)    0.500    0.056    8.879    0.000    0.500    0.402
##     .Cmptn_PE_1 (r)    0.259    0.029    8.850    0.000    0.259    0.283
##     .Cmptn_PE_2 (s)    0.446    0.043   10.376    0.000    0.446    0.358
##     .Cmptn_PE_3 (t)    0.534    0.043   12.416    0.000    0.534    0.541
##     .Cmptn_PE_4 (u)    0.397    0.035   11.213    0.000    0.397    0.415
##      f1                0.233    0.043    5.383    0.000    1.000    1.000
##      f2                0.346    0.058    5.955    0.000    1.000    1.000
##      f3                0.658    0.066    9.928    0.000    1.000    1.000
##      Atnmy_PE_1        0.000                               NaN      NaN
##      Atnmy_PE_2        0.000                               NaN      NaN
##      Atnmy_PE_3        0.000                               NaN      NaN
##      Atnmy_PE_4        0.000                               NaN      NaN
##      Rltdn_PE_1        0.000                               NaN      NaN
##      Rltdn_PE_2        0.000                               NaN      NaN
##      Rltdn_PE_3        0.000                               NaN      NaN
##      Cmptn_PE_1        0.000                               NaN      NaN
##      Cmptn_PE_2        0.000                               NaN      NaN
##      Cmptn_PE_3        0.000                               NaN      NaN
##      Cmptn_PE_4        0.000                               NaN      NaN
##      f1b               0.000                               NaN      NaN
##
##
## Group 2 [between]:
##
## Latent Variables:
##                        Estimate  Std.Err  z-value  P(>|z|)   Std.lv
##    f1 =~
##      Atn_PE_1             1.000                               1.256
##      Atn_PE_2             0.837    0.176    4.753    0.000    1.051
##      Atn_PE_3             0.793    0.180    4.404    0.000    0.996
##      Atn_PE_4             1.043    0.209    4.992    0.000    1.310
##    f2 =~
##      Rlt_PE_1             1.000                               0.719
##      Rlt_PE_2             0.952    0.232    4.105    0.000    0.685
##      Rlt_PE_3             0.653    0.188    3.473    0.001    0.470
##    f3 =~
##      Cmp_PE_1             1.000                                 NaN
##      Cmp_PE_2             0.442    0.547    0.808    0.419      NaN
##      Cmp_PE_3            -4.930    8.498   -0.580    0.562      NaN
##      Cmp_PE_4             1.312    0.638    2.056    0.040      NaN
##    Autonomy_PE_1b =~
##      Atn_PE_1 (sqc)       1.000                               1.219
##    Autonomy_PE_2b =~
##      Atn_PE_2 (sqc)       1.000                               0.520
##    Autonomy_PE_3b =~
##      Atn_PE_3 (sqc)       1.000                               0.760
##    Autonomy_PE_4b =~
##      Atn_PE_4 (sqc)       1.000                               0.709
```

```
##    Relatedness_PE_1b =~
##      Rlt_PE_1 (sqc)        1.000                                  NaN
##    Relatedness_PE_2b =~
##      Rlt_PE_2 (sqc)        1.000                                0.315
##    Relatedness_PE_3b =~
##      Rlt_PE_3 (sqc)        1.000                                  NaN
##    Competence_PE_1b =~
##      Cmp_PE_1 (sqc)        1.000                                0.844
##    Competence_PE_2b =~
##      Cmp_PE_2 (sqc)        1.000                                0.857
##    Competence_PE_3b =~
##      Cmp_PE_3 (sqc)        1.000                                1.206
##    Competence_PE_4b =~
##      Cmp_PE_4 (sqc)        1.000                                0.813
##    f1b =~
##      Atn_PE_1              1.000                                0.530
##      Atn_PE_2              1.010    0.395    2.556    0.011    1.254
##      Atn_PE_3              1.502    0.673    2.232    0.026    1.275
##      Atn_PE_4              0.995    0.378    2.635    0.008    0.906
##      Rlt_PE_1              0.420    0.374    1.122    0.262      NaN
##      Rlt_PE_2              0.553    0.373    1.482    0.138    1.135
##      Rlt_PE_3              0.313    0.264    1.187    0.235      NaN
##      Cmp_PE_1              1.290    0.770    1.675    0.094    0.987
##      Cmp_PE_2              1.313    0.809    1.623    0.105    0.989
##      Cmp_PE_3              0.732    0.498    1.468    0.142    0.392
##      Cmp_PE_4              1.044    0.645    1.619    0.105    0.828
##    Std.all
##
##      0.659
##      0.764
##      0.648
##      0.835
##
##      0.680
##      0.767
##      0.630
##
##        NaN
##        NaN
##        NaN
##        NaN
##
##      0.639
##
##      0.378
##
##      0.494
##
##      0.452
##
##        NaN
##
##      0.353
##
##        NaN
##
##      0.870
##
##      0.791
##
```

```
##     1.080
##
##     0.811
##
##     0.530
##     1.254
##     1.275
##     0.906
##       NaN
##     1.135
##       NaN
##     0.987
##     0.989
##     0.392
##     0.828
##
## Covariances:
##                    Estimate  Std.Err  z-value  P(>|z|)   Std.lv  Std.all
##   f1 ~~
##     f2                0.008    0.245    0.033    0.974    0.009    0.009
##     f3               -0.116    0.173   -0.668    0.504   -0.527   -0.527
##   f2 ~~
##     f3               -0.085    0.125   -0.676    0.499   -0.674   -0.674
##   f1 ~~
##     f1b               0.000                               0.000    0.000
##   f2 ~~
##     f1b               0.000                               0.000    0.000
##   f3 ~~
##     f1b               0.000                               0.000    0.000
##
## Variances:
##                    Estimate  Std.Err  z-value  P(>|z|)   Std.lv  Std.all
##    .Atnmy_PE_1 (j)    0.570    0.044   13.090    0.000    0.570    0.157
##    .Atnmy_PE_2 (k)    0.517    0.047   11.079    0.000    0.517    0.273
##    .Atnmy_PE_3 (l)    0.797    0.065   12.352    0.000    0.797    0.337
##    .Atnmy_PE_4 (m)    0.244    0.040    6.091    0.000    0.244    0.099
##    .Rltdn_PE_1 (n)    0.658    0.052   12.736    0.000    0.658    0.589
##    .Rltdn_PE_2 (o)    0.229    0.057    4.049    0.000    0.229    0.288
##    .Rltdn_PE_3 (p)    0.500    0.056    8.879    0.000    0.500    0.900
##    .Cmptn_PE_1 (r)    0.259    0.029    8.850    0.000    0.259    0.276
##    .Cmptn_PE_2 (s)    0.446    0.043   10.376    0.000    0.446    0.380
##    .Cmptn_PE_3 (t)    0.534    0.043   12.416    0.000    0.534    0.428
##    .Cmptn_PE_4 (u)    0.397    0.035   11.213    0.000    0.397    0.394
##     f1                1.578    0.746    2.114    0.034    1.000    1.000
##     f2                0.517    0.250    2.069    0.039    1.000    1.000
##     f3               -0.030    0.048   -0.640    0.522      NaN      NaN
##     Atnmy_PE_1        1.069    0.439    2.435    0.015    0.720    0.720
##     Atnmy_PE_2       -0.155    0.132   -1.174    0.240   -0.573   -0.573
##     Atnmy_PE_3       -0.361    0.161   -2.249    0.025   -0.625   -0.625
##     Atnmy_PE_4        0.090    0.153    0.590    0.555    0.180    0.180
##     Rltdn_PE_1       -0.132    0.170   -0.776    0.438      NaN      NaN
##     Rltdn_PE_2       -0.028    0.117   -0.243    0.808   -0.287   -0.287
##     Rltdn_PE_3       -0.206    0.103   -2.010    0.044      NaN      NaN
##     Cmptn_PE_1        0.018    0.135    0.137    0.891    0.026    0.026
##     Cmptn_PE_2        0.016    0.178    0.090    0.928    0.022    0.022
##     Cmptn_PE_3        1.231    2.037    0.604    0.546    0.847    0.847
##     Cmptn_PE_4        0.208    0.182    1.144    0.252    0.314    0.314
##     f1b               0.416    0.497    0.838    0.402    1.000    1.000
```

3x1, freely estimated loadings and latent factor variances and covariances, manifest variances set equal/fixed where necessary

```
mod_3x1_free_vareq_var <- '
f1 =~ Autonomy_PE_1 + Autonomy_PE_2 + Autonomy_PE_3 + Autonomy_PE_4
f2 =~ Relatedness_PE_1 + Relatedness_PE_2 + Relatedness_PE_3
f3 =~ Competence_PE_1 + Competence_PE_2 + Competence_PE_3 + Competence_PE_4
Autonomy_PE_1 ~~ c(j,j)*Autonomy_PE_1
Autonomy_PE_2 ~~ c(NA,0)*Autonomy_PE_2
Autonomy_PE_3 ~~ c(NA,0)*Autonomy_PE_3
Autonomy_PE_4 ~~ c(m,m)*Autonomy_PE_4
Relatedness_PE_1 ~~ c(n,n)*Relatedness_PE_1
Relatedness_PE_2 ~~ c(NA,0)*Relatedness_PE_2
Relatedness_PE_3 ~~ c(NA,0)*Relatedness_PE_3
Competence_PE_1 ~~ c(l,l)*Competence_PE_1
Competence_PE_2 ~~ c(NA,0)*Competence_PE_2
Competence_PE_3 ~~ c(NA,0)*Competence_PE_3
Competence_PE_4 ~~ c(u,u)*Competence_PE_4
f1 ~~ f1
f2 ~~ f2
f3 ~~ f3
f1 ~~ f2
f1 ~~ f3
f2 ~~ f3
Autonomy_PE_1b =~ c(0,x$sqc)*Autonomy_PE_1
Autonomy_PE_1b ~~ c(0,NA)*Autonomy_PE_1b
Autonomy_PE_2b =~ c(0,x$sqc)*Autonomy_PE_2
Autonomy_PE_2b ~~ c(0,NA)*Autonomy_PE_2b
Autonomy_PE_3b =~ c(0,x$sqc)*Autonomy_PE_3
Autonomy_PE_3b ~~ c(0,NA)*Autonomy_PE_3b
Autonomy_PE_4b =~ c(0,x$sqc)*Autonomy_PE_4
Autonomy_PE_4b ~~ c(0,NA)*Autonomy_PE_4b
Relatedness_PE_1b =~ c(0,x$sqc)*Relatedness_PE_1
Relatedness_PE_1b ~~ c(0,NA)*Relatedness_PE_1b
Relatedness_PE_2b =~ c(0,x$sqc)*Relatedness_PE_2
Relatedness_PE_2b ~~ c(0,NA)*Relatedness_PE_2b
Relatedness_PE_3b =~ c(0,x$sqc)*Relatedness_PE_3
Relatedness_PE_3b ~~ c(0,NA)*Relatedness_PE_3b
Competence_PE_1b =~ c(0,x$sqc)*Competence_PE_1
Competence_PE_1b ~~ c(0,NA)*Competence_PE_1b
Competence_PE_2b =~ c(0,x$sqc)*Competence_PE_2
Competence_PE_2b ~~ c(0,NA)*Competence_PE_2b
Competence_PE_3b =~ c(0,x$sqc)*Competence_PE_3
Competence_PE_3b ~~ c(0,NA)*Competence_PE_3b
Competence_PE_4b =~ c(0,x$sqc)*Competence_PE_4
Competence_PE_4b ~~ c(0,NA)*Competence_PE_4b
f1b =~ c(0,1)*Autonomy_PE_1b + c(0,NA)*Autonomy_PE_2b + c(0,NA)*Autonomy_PE_3b + c(0,NA)*Aut
onomy_PE_4b + c(0,NA)*Relatedness_PE_1b + c(0,NA)*Relatedness_PE_2b + c(0,NA)*Relatedness_PE
_3b + c(0,NA)*Competence_PE_1b + c(0,NA)*Competence_PE_2b + c(0,NA)*Competence_PE_3b + c(0,N
A)*Competence_PE_4b
f1b ~~ c(0,NA)*f1b + c(0,0)*f1 + c(0,0)*f2 + c(0,0)*f3
'

# Fit hypothesized model
models$mod_3x1_free_vareq_var_fit <- cfa(mod_3x1_free_vareq_var, sample.cov = combined.cov,
sample.nobs = combined.n, orthogonal = T)
```

```
## Warning in lav_object_post_check(object): lavaan WARNING: some estimated lv
## variances are negative
```

```
summary(models$mod_3x1_free_vareq_var_fit, fit.measures = T, standardized = T)
```

```
## lavaan 0.6-3 ended normally after 221 iterations
##
##   Optimization method                           NLMINB
##   Number of free parameters                         66
##   Number of equality constraints                     5
##
##   Number of observations per group
##   within                                           400
##   between                                           33
##
##   Estimator                                         ML
##   Model Fit Test Statistic                     157.964
##   Degrees of freedom                                71
##   P-value (Chi-square)                           0.000
##
## Chi-square for each group:
##
##   within                                       124.159
##   between                                       33.805
##
## Model test baseline model:
##
##   Minimum Function Test Statistic             2130.224
##   Degrees of freedom                               110
##   P-value                                        0.000
##
## User model versus baseline model:
##
##   Comparative Fit Index (CFI)                    0.957
##   Tucker-Lewis Index (TLI)                       0.933
##
## Loglikelihood and Information Criteria:
##
##   Loglikelihood user model (H0)              -5946.349
##   Loglikelihood unrestricted model (H1)      -5867.367
##
##   Number of free parameters                         61
##   Akaike (AIC)                               12014.698
##   Bayesian (BIC)                             12263.013
##   Sample-size adjusted Bayesian (BIC)        12069.432
##
## Root Mean Square Error of Approximation:
##
##   RMSEA                                          0.075
##   90 Percent Confidence Interval          0.059  0.091
##   P-value RMSEA <= 0.05                          0.005
##
## Standardized Root Mean Square Residual:
##
##   SRMR                                           0.055
```

```
## Warning in sqrt(ETA2): NaNs wurden erzeugt
```

```
## Warning in sqrt(ETA2): NaNs wurden erzeugt

## Warning in sqrt(ETA2): NaNs wurden erzeugt
```

```
##
## Parameter Estimates:
##
##   Information                                 Expected
##   Information saturated (h1) model          Structured
##   Standard Errors                             Standard
##
##
## Group 1 [within]:
##
## Latent Variables:
##                        Estimate  Std.Err  z-value  P(>|z|)   Std.lv
##   f1 =~
##     Atnmy_PE_1            1.000                               0.482
##     Atnmy_PE_2            1.545    0.157    9.842    0.000    0.745
##     Atnmy_PE_3            1.514    0.165    9.155    0.000    0.730
##     Atnmy_PE_4            1.815    0.174   10.446    0.000    0.876
##   f2 =~
##     Rltdn_PE_1            1.000                               0.589
##     Rltdn_PE_2            1.642    0.148   11.120    0.000    0.966
##     Rltdn_PE_3            1.466    0.130   11.258    0.000    0.863
##   f3 =~
##     Cmptn_PE_1            1.000                               0.811
##     Cmptn_PE_2            1.102    0.063   17.612    0.000    0.894
##     Cmptn_PE_3            0.829    0.058   14.299    0.000    0.672
##     Cmptn_PE_4            0.922    0.055   16.662    0.000    0.748
##   Autonomy_PE_1b =~
##     Atnmy_PE_1 (x)        1.000                               0.000
##   Autonomy_PE_2b =~
##     Atnmy_PE_2 (x)        1.000                               0.000
##   Autonomy_PE_3b =~
##     Atnmy_PE_3 (x)        1.000                               0.000
##   Autonomy_PE_4b =~
##     Atnmy_PE_4 (x)        1.000                               0.000
##   Relatedness_PE_1b =~
##     Rltdn_PE_1 (x)        1.000                               0.000
##   Relatedness_PE_2b =~
##     Rltdn_PE_2 (x)        1.000                               0.000
##   Relatedness_PE_3b =~
##     Rltdn_PE_3 (x)        1.000                               0.000
##   Competence_PE_1b =~
##     Cmptn_PE_1 (x)        1.000                               0.000
##   Competence_PE_2b =~
##     Cmptn_PE_2 (x)        1.000                               0.000
##   Competence_PE_3b =~
##     Cmptn_PE_3 (x)        1.000                               0.000
##   Competence_PE_4b =~
##     Cmptn_PE_4 (x)        1.000                               0.000
##   f1b =~
##     Atnmy_PE_1            0.000                                 NaN
##     Atnmy_PE_2            0.000                                 NaN
##     Atnmy_PE_3            0.000                                 NaN
##     Atnmy_PE_4            0.000                                 NaN
##     Rltdn_PE_1            0.000                                 NaN
##     Rltdn_PE_2            0.000                                 NaN
##     Rltdn_PE_3            0.000                                 NaN
##     Cmptn_PE_1            0.000                                 NaN
##     Cmptn_PE_2            0.000                                 NaN
##     Cmptn_PE_3            0.000                                 NaN
##     Cmptn_PE_4            0.000                                 NaN
```

```
##   Std.all
##
##     0.538
##     0.720
##     0.633
##     0.871
##
##     0.587
##     0.896
##     0.773
##
##     0.847
##     0.801
##     0.677
##     0.765
##
##     0.000
##
##     0.000
##
##     0.000
##
##     0.000
##
##     0.000
##
##     0.000
##
##     0.000
##
##     0.000
##
##     0.000
##
##     0.000
##
##     0.000
##
##       NaN
##       NaN
##       NaN
##       NaN
##       NaN
##       NaN
##       NaN
##       NaN
##       NaN
##       NaN
##       NaN
##
## Covariances:
##                    Estimate  Std.Err  z-value  P(>|z|)   Std.lv  Std.all
##   f1 ~~
##     f2                0.104    0.021    4.924    0.000    0.366    0.366
##     f3                0.233    0.033    7.057    0.000    0.595    0.595
##   f2 ~~
##     f3                0.199    0.034    5.923    0.000    0.416    0.416
##   f1 ~~
##     f1b               0.000                                 NaN      NaN
##   f2 ~~
```

```
##     f1b               0.000                                NaN      NaN
##   f3 ~~
##     f1b               0.000                                NaN      NaN
##
## Variances:
##                    Estimate  Std.Err  z-value  P(>|z|)   Std.lv  Std.all
##    .Atnmy_PE_1 (j)    0.570    0.044   13.090    0.000    0.570    0.710
##    .Atnmy_PE_2        0.517    0.047   11.079    0.000    0.517    0.482
##    .Atnmy_PE_3        0.797    0.065   12.352    0.000    0.797    0.599
##    .Atnmy_PE_4 (m)    0.244    0.040    6.091    0.000    0.244    0.242
##    .Rltdn_PE_1 (n)    0.658    0.052   12.736    0.000    0.658    0.655
##    .Rltdn_PE_2        0.229    0.057    4.049    0.000    0.229    0.197
##    .Rltdn_PE_3        0.500    0.056    8.879    0.000    0.500    0.402
##    .Cmptn_PE_1 (l)    0.259    0.029    8.850    0.000    0.259    0.283
##    .Cmptn_PE_2        0.446    0.043   10.376    0.000    0.446    0.358
##    .Cmptn_PE_3        0.534    0.043   12.416    0.000    0.534    0.541
##    .Cmptn_PE_4 (u)    0.397    0.035   11.213    0.000    0.397    0.415
##     f1                0.233    0.043    5.383    0.000    1.000    1.000
##     f2                0.346    0.058    5.955    0.000    1.000    1.000
##     f3                0.658    0.066    9.928    0.000    1.000    1.000
##     Atnmy_PE_1        0.000                                NaN      NaN
##     Atnmy_PE_2        0.000                                NaN      NaN
##     Atnmy_PE_3        0.000                                NaN      NaN
##     Atnmy_PE_4        0.000                                NaN      NaN
##     Rltdn_PE_1        0.000                                NaN      NaN
##     Rltdn_PE_2        0.000                                NaN      NaN
##     Rltdn_PE_3        0.000                                NaN      NaN
##     Cmptn_PE_1        0.000                                NaN      NaN
##     Cmptn_PE_2        0.000                                NaN      NaN
##     Cmptn_PE_3        0.000                                NaN      NaN
##     Cmptn_PE_4        0.000                                NaN      NaN
##     f1b               0.000                                NaN      NaN
##
##
## Group 2 [between]:
##
## Latent Variables:
##                        Estimate  Std.Err  z-value  P(>|z|)   Std.lv
##   f1 =~
##     Atn_PE_1              1.000                               1.256
##     Atn_PE_2              0.837    0.176    4.753    0.000    1.051
##     Atn_PE_3              0.793    0.180    4.404    0.000    0.996
##     Atn_PE_4              1.043    0.209    4.992    0.000    1.310
##   f2 =~
##     Rlt_PE_1              1.000                               0.719
##     Rlt_PE_2              0.952    0.232    4.105    0.000    0.685
##     Rlt_PE_3              0.653    0.188    3.473    0.001    0.470
##   f3 =~
##     Cmp_PE_1              1.000                                 NaN
##     Cmp_PE_2              0.442    0.547    0.808    0.419      NaN
##     Cmp_PE_3             -4.931    8.500   -0.580    0.562      NaN
##     Cmp_PE_4              1.312    0.638    2.056    0.040      NaN
##   Autonomy_PE_1b =~
##     Atn_PE_1 (sqc)        1.000                               1.219
##   Autonomy_PE_2b =~
##     Atn_PE_2 (sqc)        1.000                               0.887
##   Autonomy_PE_3b =~
##     Atn_PE_3 (sqc)        1.000                               1.172
##   Autonomy_PE_4b =~
##     Atn_PE_4 (sqc)        1.000                               0.709
```

```
##   Relatedness_PE_1b =~
##     Rlt_PE_1 (sqc)        1.000                                  NaN
##   Relatedness_PE_2b =~
##     Rlt_PE_2 (sqc)        1.000                                0.573
##   Relatedness_PE_3b =~
##     Rlt_PE_3 (sqc)        1.000                                0.579
##   Competence_PE_1b =~
##     Cmp_PE_1 (sqc)        1.000                                0.844
##   Competence_PE_2b =~
##     Cmp_PE_2 (sqc)        1.000                                1.086
##   Competence_PE_3b =~
##     Cmp_PE_3 (sqc)        1.000                                1.410
##   Competence_PE_4b =~
##     Cmp_PE_4 (sqc)        1.000                                0.813
##   f1b =~
##     Atn_PE_1              1.000                                0.530
##     Atn_PE_2              1.010    0.395    2.556    0.011    0.735
##     Atn_PE_3              1.502    0.673    2.232    0.026    0.827
##     Atn_PE_4              0.995    0.378    2.635    0.008    0.906
##     Rlt_PE_1              0.420    0.374    1.122    0.262      NaN
##     Rlt_PE_2              0.553    0.373    1.482    0.138    0.623
##     Rlt_PE_3              0.313    0.264    1.187    0.235    0.349
##     Cmp_PE_1              1.290    0.770    1.675    0.094    0.987
##     Cmp_PE_2              1.313    0.809    1.623    0.105    0.780
##     Cmp_PE_3              0.732    0.498    1.468    0.142    0.335
##     Cmp_PE_4              1.044    0.645    1.619    0.105    0.828
##   Std.all
##
##     0.659
##     0.764
##     0.648
##     0.835
##
##     0.680
##     0.767
##     0.630
##
##       NaN
##       NaN
##       NaN
##       NaN
##
##     0.639
##
##     0.645
##
##     0.762
##
##     0.452
##
##       NaN
##
##     0.642
##
##     0.776
##
##     0.870
##
##     1.003
##
```

```
##     1.263
##
##     0.811
##
##     0.530
##     0.735
##     0.827
##     0.906
##       NaN
##     0.623
##     0.349
##     0.987
##     0.780
##     0.335
##     0.828
##
## Covariances:
##                    Estimate  Std.Err  z-value  P(>|z|)   Std.lv  Std.all
##   f1 ~~
##     f2                0.008    0.245    0.033    0.974    0.009    0.009
##     f3               -0.116    0.173   -0.668    0.504   -0.527   -0.527
##   f2 ~~
##     f3               -0.085    0.125   -0.676    0.499   -0.674   -0.674
##   f1 ~~
##     f1b               0.000                               0.000    0.000
##   f2 ~~
##     f1b               0.000                               0.000    0.000
##   f3 ~~
##     f1b               0.000                               0.000    0.000
##
## Variances:
##                    Estimate  Std.Err  z-value  P(>|z|)   Std.lv  Std.all
##    .Atnmy_PE_1 (j)    0.570    0.044   13.090    0.000    0.570    0.157
##    .Atnmy_PE_2        0.000                               0.000    0.000
##    .Atnmy_PE_3        0.000                               0.000    0.000
##    .Atnmy_PE_4 (m)    0.244    0.040    6.091    0.000    0.244    0.099
##    .Rltdn_PE_1 (n)    0.658    0.052   12.736    0.000    0.658    0.589
##    .Rltdn_PE_2        0.000                               0.000    0.000
##    .Rltdn_PE_3        0.000                               0.000    0.000
##    .Cmptn_PE_1 (l)    0.259    0.029    8.850    0.000    0.259    0.276
##    .Cmptn_PE_2        0.000                               0.000    0.000
##    .Cmptn_PE_3        0.000                               0.000    0.000
##    .Cmptn_PE_4 (u)    0.397    0.035   11.213    0.000    0.397    0.394
##     f1                1.578    0.746    2.114    0.034    1.000    1.000
##     f2                0.517    0.250    2.069    0.039    1.000    1.000
##     f3               -0.030    0.048   -0.640    0.522      NaN      NaN
##     Atnmy_PE_1        1.069    0.439    2.435    0.015    0.720    0.720
##     Atnmy_PE_2        0.362    0.123    2.932    0.003    0.460    0.460
##     Atnmy_PE_3        0.435    0.147    2.960    0.003    0.317    0.317
##     Atnmy_PE_4        0.090    0.153    0.590    0.555    0.180    0.180
##     Rltdn_PE_1       -0.132    0.170   -0.776    0.438      NaN      NaN
##     Rltdn_PE_2        0.201    0.102    1.964    0.050    0.612    0.612
##     Rltdn_PE_3        0.294    0.086    3.427    0.001    0.878    0.878
##     Cmptn_PE_1        0.018    0.135    0.137    0.891    0.026    0.026
##     Cmptn_PE_2        0.462    0.172    2.683    0.007    0.392    0.392
##     Cmptn_PE_3        1.765    2.037    0.867    0.386    0.888    0.888
##     Cmptn_PE_4        0.208    0.182    1.144    0.252    0.314    0.314
##     f1b               0.416    0.497    0.838    0.402    1.000    1.000
```

Path diagramm

```
semPlot::semPaths(models$mod_3x1_free_vareq_var_fit,"std",edge.label.cex=0.5, curvePivot = T
RUE)
```

```
## Warning in sqrt(ETA2): NaNs wurden erzeugt

## Warning in sqrt(ETA2): NaNs wurden erzeugt

## Warning in sqrt(ETA2): NaNs wurden erzeugt
```

```
## Warning in qgraph::qgraph(Edgelist, labels = nLab, bidirectional = Bidir, :
## Non-finite weights are omitted

## Warning in qgraph::qgraph(Edgelist, labels = nLab, bidirectional = Bidir, :
## Non-finite weights are omitted
```

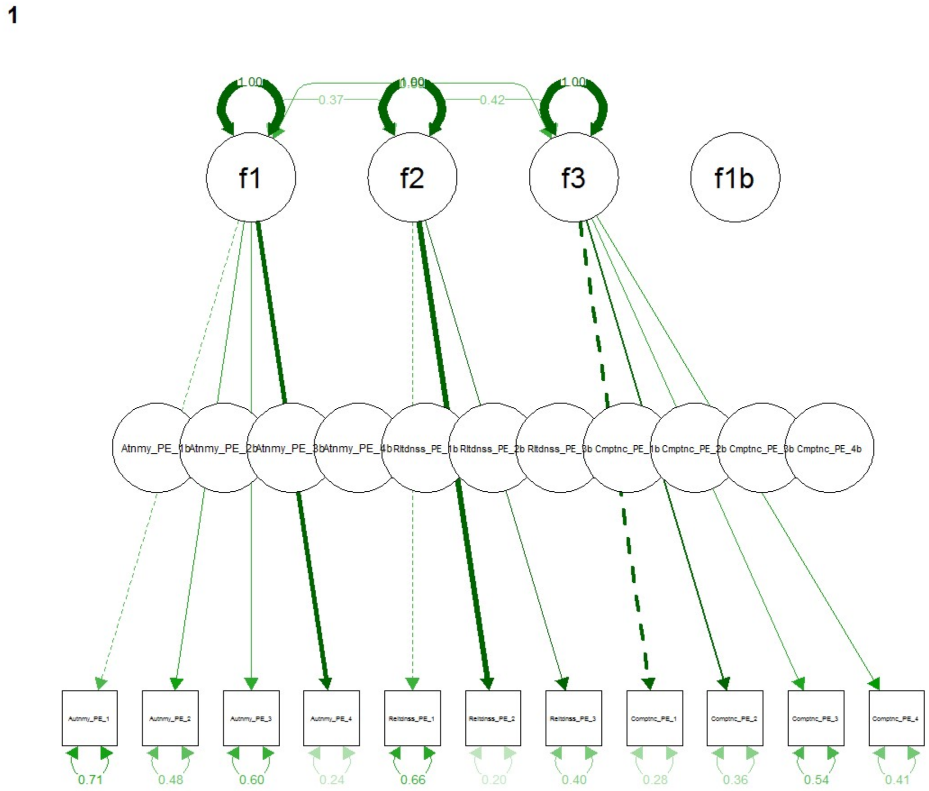

2

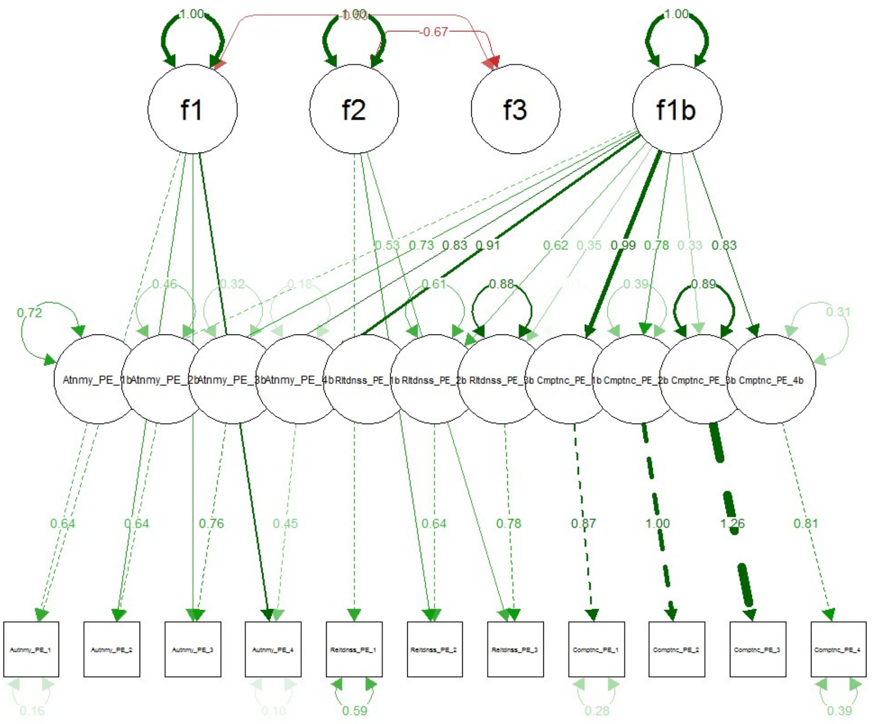

3x1, not nested

estimate 3x1-Model not nested, so 3 factors only at level 1 and one factor at level 2

```
mod_3x1_sep <- '
f1 =~ c(1,0)*Autonomy_PE_1 + c(NA,0)*Autonomy_PE_2 + c(NA,0)*Autonomy_PE_3 + c(NA,0)*Autonom
y_PE_4
f2 =~ c(1,0)*Relatedness_PE_1 + c(NA,0)*Relatedness_PE_2 + c(NA,0)*Relatedness_PE_3
f3 =~ c(1,0)*Competence_PE_1 + c(NA,0)*Competence_PE_2 + c(NA,0)*Competence_PE_3 + c(NA,0)*C
ompetence_PE_4
Autonomy_PE_1 ~~ c(j,j)*Autonomy_PE_1
Autonomy_PE_2 ~~ c(k,k)*Autonomy_PE_2
Autonomy_PE_3 ~~ c(l,l)*Autonomy_PE_3
Autonomy_PE_4 ~~ c(m,m)*Autonomy_PE_4
Relatedness_PE_1 ~~ c(n,n)*Relatedness_PE_1
Relatedness_PE_2 ~~ c(o,o)*Relatedness_PE_2
Relatedness_PE_3 ~~ c(p,p)*Relatedness_PE_3
Competence_PE_1 ~~ c(r,r)*Competence_PE_1
Competence_PE_2 ~~ c(s,s)*Competence_PE_2
Competence_PE_3 ~~ c(t,t)*Competence_PE_3
Competence_PE_4 ~~ c(u,u)*Competence_PE_4
f1 ~~ c(NA,0)*f1
f2 ~~ c(NA,0)*f2
f3 ~~ c(NA,0)*f3
f1 ~~ c(NA,0)*f2
f1 ~~ c(NA,0)*f3
f2 ~~ c(NA,0)*f3
Autonomy_PE_1b =~ c(0,x$sqc)*Autonomy_PE_1
Autonomy_PE_1b ~~ c(0,NA)*Autonomy_PE_1b
Autonomy_PE_2b =~ c(0,x$sqc)*Autonomy_PE_2
Autonomy_PE_2b ~~ c(0,NA)*Autonomy_PE_2b
Autonomy_PE_3b =~ c(0,x$sqc)*Autonomy_PE_3
Autonomy_PE_3b ~~ c(0,NA)*Autonomy_PE_3b
Autonomy_PE_4b =~ c(0,x$sqc)*Autonomy_PE_4
Autonomy_PE_4b ~~ c(0,NA)*Autonomy_PE_4b
Relatedness_PE_1b =~ c(0,x$sqc)*Relatedness_PE_1
Relatedness_PE_1b ~~ c(0,NA)*Relatedness_PE_1b
Relatedness_PE_2b =~ c(0,x$sqc)*Relatedness_PE_2
Relatedness_PE_2b ~~ c(0,NA)*Relatedness_PE_2b
Relatedness_PE_3b =~ c(0,x$sqc)*Relatedness_PE_3
Relatedness_PE_3b ~~ c(0,NA)*Relatedness_PE_3b
Competence_PE_1b =~ c(0,x$sqc)*Competence_PE_1
Competence_PE_1b ~~ c(0,NA)*Competence_PE_1b
Competence_PE_2b =~ c(0,x$sqc)*Competence_PE_2
Competence_PE_2b ~~ c(0,NA)*Competence_PE_2b
Competence_PE_3b =~ c(0,x$sqc)*Competence_PE_3
Competence_PE_3b ~~ c(0,NA)*Competence_PE_3b
Competence_PE_4b =~ c(0,x$sqc)*Competence_PE_4
Competence_PE_4b ~~ c(0,NA)*Competence_PE_4b
f1b =~ c(0,1)*Autonomy_PE_1b + c(0,NA)*Autonomy_PE_2b + c(0,NA)*Autonomy_PE_3b + c(0,NA)*Aut
onomy_PE_4b + c(0,1)*Relatedness_PE_1b + c(0,NA)*Relatedness_PE_2b + c(0,NA)*Relatedness_PE_
3b +  c(0,1)*Competence_PE_1b + c(0,NA)*Competence_PE_2b + c(0,NA)*Competence_PE_3b + c(0,N
A)*Competence_PE_4b
f1b ~~ c(0,NA)*f1b + c(0,0)*f1 + c(0,0)*f2 + c(0,0)*f3
'
# Fit hypothesized model
models$mod_3x1_sep_fit <- cfa(mod_3x1_sep, sample.cov = combined.cov, sample.nobs = combine
d.n, orthogonal = T)
```

```
## Warning in lav_object_post_check(object): lavaan WARNING: some estimated lv
## variances are negative
```

```
summary(models$mod_3x1_sep_fit, fit.measures = T, standardized = T)
```

```
## lavaan 0.6-3 ended normally after 100 iterations
##
##   Optimization method                           NLMINB
##   Number of free parameters                         56
##   Number of equality constraints                    11
##
##   Number of observations per group
##   within                                           400
##   between                                           33
##
##   Estimator                                         ML
##   Model Fit Test Statistic                     253.129
##   Degrees of freedom                                87
##   P-value (Chi-square)                           0.000
##
## Chi-square for each group:
##
##   within                                       124.159
##   between                                      128.970
##
## Model test baseline model:
##
##   Minimum Function Test Statistic             2130.224
##   Degrees of freedom                               110
##   P-value                                        0.000
##
## User model versus baseline model:
##
##   Comparative Fit Index (CFI)                    0.918
##   Tucker-Lewis Index (TLI)                       0.896
##
## Loglikelihood and Information Criteria:
##
##   Loglikelihood user model (H0)              -5993.931
##   Loglikelihood unrestricted model (H1)      -5867.367
##
##   Number of free parameters                         45
##   Akaike (AIC)                               12077.862
##   Bayesian (BIC)                             12261.045
##   Sample-size adjusted Bayesian (BIC)        12118.240
##
## Root Mean Square Error of Approximation:
##
##   RMSEA                                          0.094
##   90 Percent Confidence Interval          0.081  0.107
##   P-value RMSEA <= 0.05                          0.000
##
## Standardized Root Mean Square Residual:
##
##   SRMR                                           0.065
##
## Parameter Estimates:
##
##   Information                                 Expected
##   Information saturated (h1) model          Structured
##   Standard Errors                             Standard
##
##
## Group 1 [within]:
##
```

```
## Latent Variables:
##                          Estimate  Std.Err  z-value  P(>|z|)   Std.lv
##   f1 =~
##     Atnmy_PE_1              1.000                               0.482
##     Atnmy_PE_2              1.545    0.157    9.842    0.000    0.745
##     Atnmy_PE_3              1.514    0.165    9.155    0.000    0.730
##     Atnmy_PE_4              1.815    0.174   10.446    0.000    0.876
##   f2 =~
##     Rltdn_PE_1              1.000                               0.589
##     Rltdn_PE_2              1.642    0.148   11.120    0.000    0.966
##     Rltdn_PE_3              1.466    0.130   11.258    0.000    0.863
##   f3 =~
##     Cmptn_PE_1              1.000                               0.811
##     Cmptn_PE_2              1.102    0.063   17.612    0.000    0.894
##     Cmptn_PE_3              0.829    0.058   14.299    0.000    0.672
##     Cmptn_PE_4              0.922    0.055   16.662    0.000    0.748
##   Autonomy_PE_1b =~
##     Atnmy_PE_1 (x)          1.000                               0.000
##   Autonomy_PE_2b =~
##     Atnmy_PE_2 (x)          1.000                               0.000
##   Autonomy_PE_3b =~
##     Atnmy_PE_3 (x)          1.000                               0.000
##   Autonomy_PE_4b =~
##     Atnmy_PE_4 (x)          1.000                               0.000
##   Relatedness_PE_1b =~
##     Rltdn_PE_1 (x)          1.000                               0.000
##   Relatedness_PE_2b =~
##     Rltdn_PE_2 (x)          1.000                               0.000
##   Relatedness_PE_3b =~
##     Rltdn_PE_3 (x)          1.000                               0.000
##   Competence_PE_1b =~
##     Cmptn_PE_1 (x)          1.000                               0.000
##   Competence_PE_2b =~
##     Cmptn_PE_2 (x)          1.000                               0.000
##   Competence_PE_3b =~
##     Cmptn_PE_3 (x)          1.000                               0.000
##   Competence_PE_4b =~
##     Cmptn_PE_4 (x)          1.000                               0.000
##   f1b =~
##     Atnmy_PE_1              0.000                                 NaN
##     Atnmy_PE_2              0.000                                 NaN
##     Atnmy_PE_3              0.000                                 NaN
##     Atnmy_PE_4              0.000                                 NaN
##     Rltdn_PE_1              0.000                                 NaN
##     Rltdn_PE_2              0.000                                 NaN
##     Rltdn_PE_3              0.000                                 NaN
##     Cmptn_PE_1              0.000                                 NaN
##     Cmptn_PE_2              0.000                                 NaN
##     Cmptn_PE_3              0.000                                 NaN
##     Cmptn_PE_4              0.000                                 NaN
##   Std.all
##
##     0.538
##     0.720
##     0.633
##     0.871
##
##     0.587
##     0.896
##     0.773
```

```
##
##     0.847
##     0.801
##     0.677
##     0.765
##
##     0.000
##
##     0.000
##
##     0.000
##
##     0.000
##
##     0.000
##
##     0.000
##
##     0.000
##
##     0.000
##
##     0.000
##
##     0.000
##
##     0.000
##
##       NaN
##       NaN
##       NaN
##       NaN
##       NaN
##       NaN
##       NaN
##       NaN
##       NaN
##       NaN
##       NaN
##
## Covariances:
##                    Estimate  Std.Err  z-value  P(>|z|)   Std.lv  Std.all
##   f1 ~~
##     f2                0.104    0.021    4.924    0.000    0.366    0.366
##     f3                0.233    0.033    7.057    0.000    0.595    0.595
##   f2 ~~
##     f3                0.199    0.034    5.923    0.000    0.416    0.416
##   f1 ~~
##     f1b               0.000                                 NaN      NaN
##   f2 ~~
##     f1b               0.000                                 NaN      NaN
##   f3 ~~
##     f1b               0.000                                 NaN      NaN
##
## Variances:
##                    Estimate  Std.Err  z-value  P(>|z|)   Std.lv  Std.all
##    .Atnmy_PE_1 (j)    0.570    0.044   13.090    0.000    0.570    0.710
##    .Atnmy_PE_2 (k)    0.517    0.047   11.079    0.000    0.517    0.482
##    .Atnmy_PE_3 (l)    0.797    0.065   12.352    0.000    0.797    0.599
##    .Atnmy_PE_4 (m)    0.244    0.040    6.091    0.000    0.244    0.242
```

```
##    .Rltdn_PE_1 (n)    0.658    0.052   12.736    0.000    0.658    0.655
##    .Rltdn_PE_2 (o)    0.229    0.057    4.049    0.000    0.229    0.197
##    .Rltdn_PE_3 (p)    0.500    0.056    8.879    0.000    0.500    0.402
##    .Cmptn_PE_1 (r)    0.259    0.029    8.850    0.000    0.259    0.283
##    .Cmptn_PE_2 (s)    0.446    0.043   10.376    0.000    0.446    0.358
##    .Cmptn_PE_3 (t)    0.534    0.043   12.416    0.000    0.534    0.541
##    .Cmptn_PE_4 (u)    0.397    0.035   11.213    0.000    0.397    0.415
##     f1                0.233    0.043    5.383    0.000    1.000    1.000
##     f2                0.346    0.058    5.955    0.000    1.000    1.000
##     f3                0.658    0.066    9.928    0.000    1.000    1.000
##     Atnmy_PE_1        0.000                                 NaN      NaN
##     Atnmy_PE_2        0.000                                 NaN      NaN
##     Atnmy_PE_3        0.000                                 NaN      NaN
##     Atnmy_PE_4        0.000                                 NaN      NaN
##     Rltdn_PE_1        0.000                                 NaN      NaN
##     Rltdn_PE_2        0.000                                 NaN      NaN
##     Rltdn_PE_3        0.000                                 NaN      NaN
##     Cmptn_PE_1        0.000                                 NaN      NaN
##     Cmptn_PE_2        0.000                                 NaN      NaN
##     Cmptn_PE_3        0.000                                 NaN      NaN
##     Cmptn_PE_4        0.000                                 NaN      NaN
##     f1b               0.000                                 NaN      NaN
##
##
## Group 2 [between]:
##
## Latent Variables:
##                        Estimate  Std.Err  z-value  P(>|z|)   Std.lv
##   f1 =~
##     Atn_PE_1              0.000                               0.000
##     Atn_PE_2              0.000                               0.000
##     Atn_PE_3              0.000                               0.000
##     Atn_PE_4              0.000                               0.000
##   f2 =~
##     Rlt_PE_1              0.000                               0.000
##     Rlt_PE_2              0.000                               0.000
##     Rlt_PE_3              0.000                               0.000
##   f3 =~
##     Cmp_PE_1              0.000                               0.000
##     Cmp_PE_2              0.000                               0.000
##     Cmp_PE_3              0.000                               0.000
##     Cmp_PE_4              0.000                               0.000
##   Autonomy_PE_1b =~
##     Atn_PE_1 (sqc)        1.000                               1.515
##   Autonomy_PE_2b =~
##     Atn_PE_2 (sqc)        1.000                               1.173
##   Autonomy_PE_3b =~
##     Atn_PE_3 (sqc)        1.000                               1.253
##   Autonomy_PE_4b =~
##     Atn_PE_4 (sqc)        1.000                               1.490
##   Relatedness_PE_1b =~
##     Rlt_PE_1 (sqc)        1.000                               0.813
##   Relatedness_PE_2b =~
##     Rlt_PE_2 (sqc)        1.000                               0.754
##   Relatedness_PE_3b =~
##     Rlt_PE_3 (sqc)        1.000                               0.235
##   Competence_PE_1b =~
##     Cmp_PE_1 (sqc)        1.000                               0.871
##   Competence_PE_2b =~
##     Cmp_PE_2 (sqc)        1.000                               0.853
```

```
##   Competence_PE_3b =~
##     Cmp_PE_3 (sqc)        1.000                                   0.844
##   Competence_PE_4b =~
##     Cmp_PE_4 (sqc)        1.000                                   0.781
##   f1b =~
##     Atn_PE_1              1.000                                   0.341
##     Atn_PE_2              2.301    0.552    4.170    0.000    1.015
##     Atn_PE_3              2.693    0.626    4.302    0.000    1.112
##     Atn_PE_4              2.629    0.630    4.175    0.000    0.913
##     Rlt_PE_1              1.000                                   0.637
##     Rlt_PE_2              0.537    0.318    1.687    0.092    0.369
##     Rlt_PE_3              0.393    0.264    1.488    0.137    0.865
##     Cmp_PE_1              1.000                                   0.594
##     Cmp_PE_2              1.137    0.401    2.835    0.005    0.690
##     Cmp_PE_3              1.401    0.423    3.311    0.001    0.858
##     Cmp_PE_4              0.739    0.361    2.048    0.041    0.490
##   Std.all
##
##     0.000
##     0.000
##     0.000
##     0.000
##
##     0.000
##     0.000
##     0.000
##
##     0.000
##     0.000
##     0.000
##     0.000
##
##     0.895
##
##     0.853
##
##     0.814
##
##     0.949
##
##     0.708
##
##     0.844
##
##     0.316
##
##     0.863
##
##     0.787
##
##     0.756
##
##     0.778
##
##     0.341
##     1.015
##     1.112
##     0.913
##     0.637
##     0.369
```

```
##     0.865
##     0.594
##     0.690
##     0.858
##     0.490
##
## Covariances:
##                    Estimate  Std.Err  z-value  P(>|z|)   Std.lv  Std.all
##   f1 ~~
##     f2                0.000                               NaN      NaN
##     f3                0.000                               NaN      NaN
##   f2 ~~
##     f3                0.000                               NaN      NaN
##   f1 ~~
##     f1b               0.000                               NaN      NaN
##   f2 ~~
##     f1b               0.000                               NaN      NaN
##   f3 ~~
##     f1b               0.000                               NaN      NaN
##
## Variances:
##                    Estimate  Std.Err  z-value  P(>|z|)   Std.lv  Std.all
##    .Atnmy_PE_1 (j)    0.570    0.044   13.090    0.000    0.570    0.199
##    .Atnmy_PE_2 (k)    0.517    0.047   11.079    0.000    0.517    0.273
##    .Atnmy_PE_3 (l)    0.797    0.065   12.352    0.000    0.797    0.337
##    .Atnmy_PE_4 (m)    0.244    0.040    6.091    0.000    0.244    0.099
##    .Rltdn_PE_1 (n)    0.658    0.052   12.736    0.000    0.658    0.499
##    .Rltdn_PE_2 (o)    0.229    0.057    4.049    0.000    0.229    0.288
##    .Rltdn_PE_3 (p)    0.500    0.056    8.879    0.000    0.500    0.900
##    .Cmptn_PE_1 (r)    0.259    0.029    8.850    0.000    0.259    0.255
##    .Cmptn_PE_2 (s)    0.446    0.043   10.376    0.000    0.446    0.380
##    .Cmptn_PE_3 (t)    0.534    0.043   12.416    0.000    0.534    0.428
##    .Cmptn_PE_4 (u)    0.397    0.035   11.213    0.000    0.397    0.394
##     f1                0.000                               NaN      NaN
##     f2                0.000                               NaN      NaN
##     f3                0.000                               NaN      NaN
##     Atnmy_PE_1        2.029    0.646    3.140    0.002    0.883    0.883
##     Atnmy_PE_2       -0.042    0.162   -0.257    0.798   -0.030   -0.030
##     Atnmy_PE_3       -0.370    0.180   -2.059    0.039   -0.236   -0.236
##     Atnmy_PE_4        0.369    0.205    1.799    0.072    0.166    0.166
##     Rltdn_PE_1        0.393    0.269    1.462    0.144    0.595    0.595
##     Rltdn_PE_2        0.491    0.188    2.617    0.009    0.864    0.864
##     Rltdn_PE_3        0.014    0.139    0.100    0.920    0.252    0.252
##     Cmptn_PE_1        0.490    0.192    2.555    0.011    0.647    0.647
##     Cmptn_PE_2        0.382    0.215    1.774    0.076    0.524    0.524
##     Cmptn_PE_3        0.188    0.193    0.972    0.331    0.264    0.264
##     Cmptn_PE_4        0.463    0.217    2.129    0.033    0.760    0.760
##     f1b               0.268    0.132    2.025    0.043    1.000    1.000
```

3x1, not nested, var set to 0

Model B

```
mod_3x1_sep_var <- '
f1 =~ c(1,0)*Autonomy_PE_1 + c(NA,0)*Autonomy_PE_2 + c(NA,0)*Autonomy_PE_3 + c(NA,0)*Autonom
y_PE_4
f2 =~ c(1,0)*Relatedness_PE_1 + c(NA,0)*Relatedness_PE_2 + c(NA,0)*Relatedness_PE_3
f3 =~ c(1,0)*Competence_PE_1 + c(NA,0)*Competence_PE_2 + c(NA,0)*Competence_PE_3 + c(NA,0)*C
ompetence_PE_4
Autonomy_PE_1 ~~ c(j,j)*Autonomy_PE_1
Autonomy_PE_2 ~~ c(NA,0)*Autonomy_PE_2
Autonomy_PE_3 ~~ c(NA,0)*Autonomy_PE_3
Autonomy_PE_4 ~~ c(m,m)*Autonomy_PE_4
Relatedness_PE_1 ~~ c(n,n)*Relatedness_PE_1
Relatedness_PE_2 ~~ c(o,o)*Relatedness_PE_2
Relatedness_PE_3 ~~ c(p,p)*Relatedness_PE_3
Competence_PE_1 ~~ c(r,r)*Competence_PE_1
Competence_PE_2 ~~ c(s,s)*Competence_PE_2
Competence_PE_3 ~~ c(t,t)*Competence_PE_3
Competence_PE_4 ~~ c(u,u)*Competence_PE_4
f1 ~~ c(NA,0)*f1
f2 ~~ c(NA,0)*f2
f3 ~~ c(NA,0)*f3
f1 ~~ c(NA,0)*f2
f1 ~~ c(NA,0)*f3
f2 ~~ c(NA,0)*f3
Autonomy_PE_1b =~ c(0,x$sqc)*Autonomy_PE_1
Autonomy_PE_1b ~~ c(0,NA)*Autonomy_PE_1b
Autonomy_PE_2b =~ c(0,x$sqc)*Autonomy_PE_2
Autonomy_PE_2b ~~ c(0,NA)*Autonomy_PE_2b
Autonomy_PE_3b =~ c(0,x$sqc)*Autonomy_PE_3
Autonomy_PE_3b ~~ c(0,NA)*Autonomy_PE_3b
Autonomy_PE_4b =~ c(0,x$sqc)*Autonomy_PE_4
Autonomy_PE_4b ~~ c(0,NA)*Autonomy_PE_4b
Relatedness_PE_1b =~ c(0,x$sqc)*Relatedness_PE_1
Relatedness_PE_1b ~~ c(0,NA)*Relatedness_PE_1b
Relatedness_PE_2b =~ c(0,x$sqc)*Relatedness_PE_2
Relatedness_PE_2b ~~ c(0,NA)*Relatedness_PE_2b
Relatedness_PE_3b =~ c(0,x$sqc)*Relatedness_PE_3
Relatedness_PE_3b ~~ c(0,NA)*Relatedness_PE_3b
Competence_PE_1b =~ c(0,x$sqc)*Competence_PE_1
Competence_PE_1b ~~ c(0,NA)*Competence_PE_1b
Competence_PE_2b =~ c(0,x$sqc)*Competence_PE_2
Competence_PE_2b ~~ c(0,NA)*Competence_PE_2b
Competence_PE_3b =~ c(0,x$sqc)*Competence_PE_3
Competence_PE_3b ~~ c(0,NA)*Competence_PE_3b
Competence_PE_4b =~ c(0,x$sqc)*Competence_PE_4
Competence_PE_4b ~~ c(0,NA)*Competence_PE_4b
f1b =~ c(0,1)*Autonomy_PE_1b + c(0,NA)*Autonomy_PE_2b + c(0,NA)*Autonomy_PE_3b + c(0,NA)*Aut
onomy_PE_4b + c(0,1)*Relatedness_PE_1b + c(0,NA)*Relatedness_PE_2b + c(0,NA)*Relatedness_PE_
3b + c(0,1)*Competence_PE_1b + c(0,NA)*Competence_PE_2b + c(0,NA)*Competence_PE_3b + c(0,NA)
*Competence_PE_4b
f1b ~~ c(0,NA)*f1b + c(0,0)*f1 + c(0,0)*f2 + c(0,0)*f3
'
# Fit hypothesized model
models$mod_3x1_sep_var_fit <- cfa(mod_3x1_sep_var, sample.cov = combined.cov, sample.nobs =
combined.n, orthogonal = T)
summary(models$mod_3x1_sep_var_fit, fit.measures = T, standardized = T)
```

```
## lavaan 0.6-3 ended normally after 97 iterations
##
##   Optimization method                           NLMINB
##   Number of free parameters                         54
##   Number of equality constraints                     9
##
##   Number of observations per group
##   within                                           400
##   between                                           33
##
##   Estimator                                         ML
##   Model Fit Test Statistic                     253.129
##   Degrees of freedom                                87
##   P-value (Chi-square)                           0.000
##
## Chi-square for each group:
##
##   within                                       124.159
##   between                                      128.970
##
## Model test baseline model:
##
##   Minimum Function Test Statistic             2130.224
##   Degrees of freedom                               110
##   P-value                                        0.000
##
## User model versus baseline model:
##
##   Comparative Fit Index (CFI)                    0.918
##   Tucker-Lewis Index (TLI)                       0.896
##
## Loglikelihood and Information Criteria:
##
##   Loglikelihood user model (H0)              -5993.931
##   Loglikelihood unrestricted model (H1)      -5867.367
##
##   Number of free parameters                         45
##   Akaike (AIC)                               12077.862
##   Bayesian (BIC)                             12261.045
##   Sample-size adjusted Bayesian (BIC)        12118.240
##
## Root Mean Square Error of Approximation:
##
##   RMSEA                                          0.094
##   90 Percent Confidence Interval          0.081  0.107
##   P-value RMSEA <= 0.05                          0.000
##
## Standardized Root Mean Square Residual:
##
##   SRMR                                           0.065
##
## Parameter Estimates:
##
##   Information                                 Expected
##   Information saturated (h1) model          Structured
##   Standard Errors                             Standard
##
##
## Group 1 [within]:
##
```

```
## Latent Variables:
##                       Estimate  Std.Err  z-value  P(>|z|)   Std.lv
##   f1 =~
##     Atnmy_PE_1           1.000                               0.482
##     Atnmy_PE_2           1.545    0.157    9.842    0.000    0.745
##     Atnmy_PE_3           1.514    0.165    9.155    0.000    0.730
##     Atnmy_PE_4           1.815    0.174   10.446    0.000    0.876
##   f2 =~
##     Rltdn_PE_1           1.000                               0.589
##     Rltdn_PE_2           1.642    0.148   11.120    0.000    0.966
##     Rltdn_PE_3           1.466    0.130   11.258    0.000    0.863
##   f3 =~
##     Cmptn_PE_1           1.000                               0.811
##     Cmptn_PE_2           1.102    0.063   17.612    0.000    0.894
##     Cmptn_PE_3           0.829    0.058   14.299    0.000    0.672
##     Cmptn_PE_4           0.922    0.055   16.662    0.000    0.748
##   Autonomy_PE_1b =~
##     Atnmy_PE_1 (x)       1.000                               0.000
##   Autonomy_PE_2b =~
##     Atnmy_PE_2 (x)       1.000                               0.000
##   Autonomy_PE_3b =~
##     Atnmy_PE_3 (x)       1.000                               0.000
##   Autonomy_PE_4b =~
##     Atnmy_PE_4 (x)       1.000                               0.000
##   Relatedness_PE_1b =~
##     Rltdn_PE_1 (x)       1.000                               0.000
##   Relatedness_PE_2b =~
##     Rltdn_PE_2 (x)       1.000                               0.000
##   Relatedness_PE_3b =~
##     Rltdn_PE_3 (x)       1.000                               0.000
##   Competence_PE_1b =~
##     Cmptn_PE_1 (x)       1.000                               0.000
##   Competence_PE_2b =~
##     Cmptn_PE_2 (x)       1.000                               0.000
##   Competence_PE_3b =~
##     Cmptn_PE_3 (x)       1.000                               0.000
##   Competence_PE_4b =~
##     Cmptn_PE_4 (x)       1.000                               0.000
##   f1b =~
##     Atnmy_PE_1           0.000                                 NaN
##     Atnmy_PE_2           0.000                                 NaN
##     Atnmy_PE_3           0.000                                 NaN
##     Atnmy_PE_4           0.000                                 NaN
##     Rltdn_PE_1           0.000                                 NaN
##     Rltdn_PE_2           0.000                                 NaN
##     Rltdn_PE_3           0.000                                 NaN
##     Cmptn_PE_1           0.000                                 NaN
##     Cmptn_PE_2           0.000                                 NaN
##     Cmptn_PE_3           0.000                                 NaN
##     Cmptn_PE_4           0.000                                 NaN
##   Std.all
##   
##     0.538
##     0.720
##     0.633
##     0.871
##   
##     0.587
##     0.896
##     0.773
```

```
##
##     0.847
##     0.801
##     0.677
##     0.765
##
##     0.000
##
##     0.000
##
##     0.000
##
##     0.000
##
##     0.000
##
##     0.000
##
##     0.000
##
##     0.000
##
##     0.000
##
##     0.000
##
##     0.000
##
##       NaN
##       NaN
##       NaN
##       NaN
##       NaN
##       NaN
##       NaN
##       NaN
##       NaN
##       NaN
##       NaN
##
## Covariances:
##                    Estimate  Std.Err  z-value  P(>|z|)   Std.lv  Std.all
##   f1 ~~
##     f2                0.104    0.021    4.924    0.000    0.366    0.366
##     f3                0.233    0.033    7.057    0.000    0.595    0.595
##   f2 ~~
##     f3                0.199    0.034    5.923    0.000    0.416    0.416
##   f1 ~~
##     f1b               0.000                                  NaN      NaN
##   f2 ~~
##     f1b               0.000                                  NaN      NaN
##   f3 ~~
##     f1b               0.000                                  NaN      NaN
##
## Variances:
##                    Estimate  Std.Err  z-value  P(>|z|)   Std.lv  Std.all
##    .Atnmy_PE_1 (j)     0.570    0.044   13.090    0.000    0.570    0.710
##    .Atnmy_PE_2         0.517    0.047   11.079    0.000    0.517    0.482
##    .Atnmy_PE_3         0.797    0.065   12.352    0.000    0.797    0.599
##    .Atnmy_PE_4 (m)     0.244    0.040    6.091    0.000    0.244    0.242
```

```
##    .Rltdn_PE_1 (n)    0.658    0.052   12.736    0.000    0.658    0.655
##    .Rltdn_PE_2 (o)    0.229    0.057    4.049    0.000    0.229    0.197
##    .Rltdn_PE_3 (p)    0.500    0.056    8.879    0.000    0.500    0.402
##    .Cmptn_PE_1 (r)    0.259    0.029    8.850    0.000    0.259    0.283
##    .Cmptn_PE_2 (s)    0.446    0.043   10.376    0.000    0.446    0.358
##    .Cmptn_PE_3 (t)    0.534    0.043   12.416    0.000    0.534    0.541
##    .Cmptn_PE_4 (u)    0.397    0.035   11.213    0.000    0.397    0.415
##     f1                0.233    0.043    5.383    0.000    1.000    1.000
##     f2                0.346    0.058    5.955    0.000    1.000    1.000
##     f3                0.658    0.066    9.928    0.000    1.000    1.000
##     Atnmy_PE_1        0.000                                NaN      NaN
##     Atnmy_PE_2        0.000                                NaN      NaN
##     Atnmy_PE_3        0.000                                NaN      NaN
##     Atnmy_PE_4        0.000                                NaN      NaN
##     Rltdn_PE_1        0.000                                NaN      NaN
##     Rltdn_PE_2        0.000                                NaN      NaN
##     Rltdn_PE_3        0.000                                NaN      NaN
##     Cmptn_PE_1        0.000                                NaN      NaN
##     Cmptn_PE_2        0.000                                NaN      NaN
##     Cmptn_PE_3        0.000                                NaN      NaN
##     Cmptn_PE_4        0.000                                NaN      NaN
##     f1b               0.000                                NaN      NaN
##
##
## Group 2 [between]:
##
## Latent Variables:
##                        Estimate  Std.Err  z-value  P(>|z|)   Std.lv
##   f1 =~
##     Atn_PE_1              0.000                               0.000
##     Atn_PE_2              0.000                               0.000
##     Atn_PE_3              0.000                               0.000
##     Atn_PE_4              0.000                               0.000
##   f2 =~
##     Rlt_PE_1              0.000                               0.000
##     Rlt_PE_2              0.000                               0.000
##     Rlt_PE_3              0.000                               0.000
##   f3 =~
##     Cmp_PE_1              0.000                               0.000
##     Cmp_PE_2              0.000                               0.000
##     Cmp_PE_3              0.000                               0.000
##     Cmp_PE_4              0.000                               0.000
##   Autonomy_PE_1b =~
##     Atn_PE_1 (sqc)        1.000                               1.515
##   Autonomy_PE_2b =~
##     Atn_PE_2 (sqc)        1.000                               1.375
##   Autonomy_PE_3b =~
##     Atn_PE_3 (sqc)        1.000                               1.539
##   Autonomy_PE_4b =~
##     Atn_PE_4 (sqc)        1.000                               1.490
##   Relatedness_PE_1b =~
##     Rlt_PE_1 (sqc)        1.000                               0.813
##   Relatedness_PE_2b =~
##     Rlt_PE_2 (sqc)        1.000                               0.754
##   Relatedness_PE_3b =~
##     Rlt_PE_3 (sqc)        1.000                               0.235
##   Competence_PE_1b =~
##     Cmp_PE_1 (sqc)        1.000                               0.871
##   Competence_PE_2b =~
##     Cmp_PE_2 (sqc)        1.000                               0.853
```

```
##   Competence_PE_3b =~
##     Cmp_PE_3 (sqc)        1.000                                   0.844
##   Competence_PE_4b =~
##     Cmp_PE_4 (sqc)        1.000                                   0.781
##   f1b =~
##     Atn_PE_1              1.000                                   0.341
##     Atn_PE_2              2.301    0.552    4.170    0.000    0.865
##     Atn_PE_3              2.693    0.626    4.302    0.000    0.905
##     Atn_PE_4              2.629    0.630    4.175    0.000    0.913
##     Rlt_PE_1              1.000                                   0.637
##     Rlt_PE_2              0.537    0.318    1.687    0.092    0.369
##     Rlt_PE_3              0.393    0.264    1.488    0.137    0.865
##     Cmp_PE_1              1.000                                   0.594
##     Cmp_PE_2              1.137    0.401    2.835    0.005    0.690
##     Cmp_PE_3              1.401    0.423    3.311    0.001    0.858
##     Cmp_PE_4              0.739    0.361    2.048    0.041    0.490
##   Std.all
##
##     0.000
##     0.000
##     0.000
##     0.000
##
##     0.000
##     0.000
##     0.000
##
##     0.000
##     0.000
##     0.000
##     0.000
##
##     0.895
##
##     1.000
##
##     1.000
##
##     0.949
##
##     0.708
##
##     0.844
##
##     0.316
##
##     0.863
##
##     0.787
##
##     0.756
##
##     0.778
##
##     0.341
##     0.865
##     0.905
##     0.913
##     0.637
##     0.369
```

```
##     0.865
##     0.594
##     0.690
##     0.858
##     0.490
##
## Covariances:
##                    Estimate  Std.Err  z-value  P(>|z|)   Std.lv  Std.all
##   f1 ~~
##     f2                0.000                                 NaN      NaN
##     f3                0.000                                 NaN      NaN
##   f2 ~~
##     f3                0.000                                 NaN      NaN
##   f1 ~~
##     f1b               0.000                                 NaN      NaN
##   f2 ~~
##     f1b               0.000                                 NaN      NaN
##   f3 ~~
##     f1b               0.000                                 NaN      NaN
##
## Variances:
##                    Estimate  Std.Err  z-value  P(>|z|)   Std.lv  Std.all
##    .Atnmy_PE_1 (j)    0.570    0.044   13.090    0.000    0.570    0.199
##    .Atnmy_PE_2        0.000                               0.000    0.000
##    .Atnmy_PE_3        0.000                               0.000    0.000
##    .Atnmy_PE_4 (m)    0.244    0.040    6.091    0.000    0.244    0.099
##    .Rltdn_PE_1 (n)    0.658    0.052   12.736    0.000    0.658    0.499
##    .Rltdn_PE_2 (o)    0.229    0.057    4.049    0.000    0.229    0.288
##    .Rltdn_PE_3 (p)    0.500    0.056    8.879    0.000    0.500    0.900
##    .Cmptn_PE_1 (r)    0.259    0.029    8.850    0.000    0.259    0.255
##    .Cmptn_PE_2 (s)    0.446    0.043   10.376    0.000    0.446    0.380
##    .Cmptn_PE_3 (t)    0.534    0.043   12.416    0.000    0.534    0.428
##    .Cmptn_PE_4 (u)    0.397    0.035   11.213    0.000    0.397    0.394
##     f1                0.000                                 NaN      NaN
##     f2                0.000                                 NaN      NaN
##     f3                0.000                                 NaN      NaN
##     Atnmy_PE_1        2.029    0.646    3.140    0.002    0.883    0.883
##     Atnmy_PE_2        0.475    0.155    3.061    0.002    0.251    0.251
##     Atnmy_PE_3        0.427    0.168    2.543    0.011    0.180    0.180
##     Atnmy_PE_4        0.369    0.205    1.799    0.072    0.166    0.166
##     Rltdn_PE_1        0.393    0.269    1.462    0.144    0.595    0.595
##     Rltdn_PE_2        0.491    0.188    2.617    0.009    0.864    0.864
##     Rltdn_PE_3        0.014    0.139    0.100    0.920    0.252    0.252
##     Cmptn_PE_1        0.490    0.192    2.555    0.011    0.647    0.647
##     Cmptn_PE_2        0.382    0.215    1.774    0.076    0.524    0.524
##     Cmptn_PE_3        0.188    0.193    0.972    0.331    0.264    0.264
##     Cmptn_PE_4        0.463    0.217    2.130    0.033    0.760    0.760
##     f1b               0.268    0.132    2.025    0.043    1.000    1.000
```

see Fit indices of Model B in table 2

Path diagramm

```
semPlot::semPaths(models$mod_3x1_sep_var_fit,"std",edge.label.cex=0.5, curvePivot = TRUE)
```

```
## Warning in qgraph::qgraph(Edgelist, labels = nLab, bidirectional = Bidir, :
## Non-finite weights are omitted

## Warning in qgraph::qgraph(Edgelist, labels = nLab, bidirectional = Bidir, :
## Non-finite weights are omitted
```

1

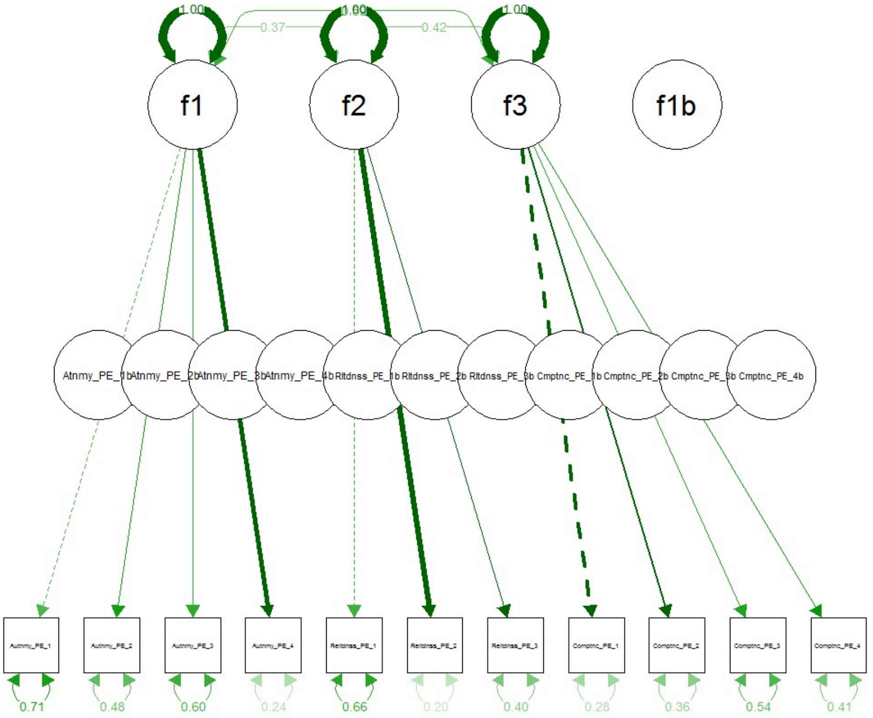

2

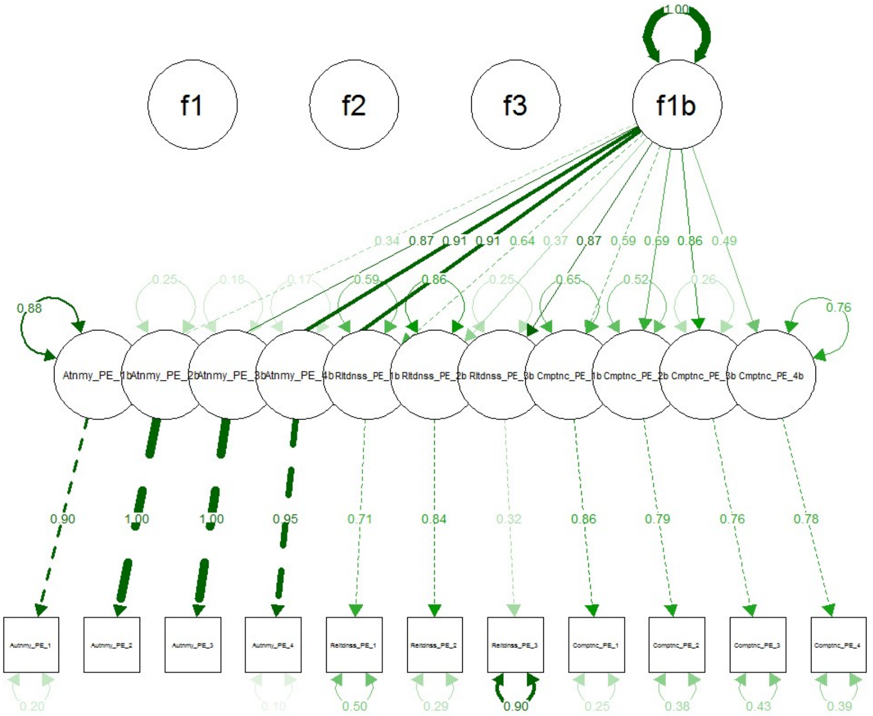

3x3-Modell

3 latent overall factors at level 2

```
mod_3x3 <- '
f1 =~ Autonomy_PE_1 + c(a,a)*Autonomy_PE_2 + c(b,b)*Autonomy_PE_3 + c(c,c)*Autonomy_PE_4
f2 =~ Relatedness_PE_1 + c(d,d)*Relatedness_PE_2 + c(e,e)*Relatedness_PE_3
f3 =~ Competence_PE_1 + c(g,g)*Competence_PE_2 + c(h,h)*Competence_PE_3 + c(i,i)*Competence_
PE_4
Autonomy_PE_1 ~~ c(j,j)*Autonomy_PE_1
Autonomy_PE_2 ~~ c(k,k)*Autonomy_PE_2
Autonomy_PE_3 ~~ c(l,l)*Autonomy_PE_3
Autonomy_PE_4 ~~ c(m,m)*Autonomy_PE_4
Relatedness_PE_1 ~~ c(n,n)*Relatedness_PE_1
Relatedness_PE_2 ~~ c(o,o)*Relatedness_PE_2
Relatedness_PE_3 ~~ c(p,p)*Relatedness_PE_3
Competence_PE_1 ~~ c(r,r)*Competence_PE_1
Competence_PE_2 ~~ c(s,s)*Competence_PE_2
Competence_PE_3 ~~ c(t,t)*Competence_PE_3
Competence_PE_4 ~~ c(u,u)*Competence_PE_4
f1 ~~ c(v,v)*f1
f2 ~~ c(w,w)*f2
f3 ~~ c(x,x)*f3
f1 ~~ c(y,y)*f2
f1 ~~ c(z,z)*f3
f2 ~~ c(aa,aa)*f3
Autonomy_PE_1b =~ c(0,x$sqc)*Autonomy_PE_1
Autonomy_PE_1b ~~ c(0,NA)*Autonomy_PE_1b
Autonomy_PE_2b =~ c(0,x$sqc)*Autonomy_PE_2
Autonomy_PE_2b ~~ c(0,NA)*Autonomy_PE_2b
Autonomy_PE_3b =~ c(0,x$sqc)*Autonomy_PE_3
Autonomy_PE_3b ~~ c(0,NA)*Autonomy_PE_3b
Autonomy_PE_4b =~ c(0,x$sqc)*Autonomy_PE_4
Autonomy_PE_4b ~~ c(0,NA)*Autonomy_PE_4b
Relatedness_PE_1b =~ c(0,x$sqc)*Relatedness_PE_1
Relatedness_PE_1b ~~ c(0,NA)*Relatedness_PE_1b
Relatedness_PE_2b =~ c(0,x$sqc)*Relatedness_PE_2
Relatedness_PE_2b ~~ c(0,NA)*Relatedness_PE_2b
Relatedness_PE_3b =~ c(0,x$sqc)*Relatedness_PE_3
Relatedness_PE_3b ~~ c(0,NA)*Relatedness_PE_3b
Competence_PE_1b =~ c(0,x$sqc)*Competence_PE_1
Competence_PE_1b ~~ c(0,NA)*Competence_PE_1b
Competence_PE_2b =~ c(0,x$sqc)*Competence_PE_2
Competence_PE_2b ~~ c(0,NA)*Competence_PE_2b
Competence_PE_3b =~ c(0,x$sqc)*Competence_PE_3
Competence_PE_3b ~~ c(0,NA)*Competence_PE_3b
Competence_PE_4b =~ c(0,x$sqc)*Competence_PE_4
Competence_PE_4b ~~ c(0,NA)*Competence_PE_4b
f1b =~ c(0,1)*Autonomy_PE_1b + c(0,NA)*Autonomy_PE_2b + c(0,NA)*Autonomy_PE_3b + c(0,NA)*Aut
onomy_PE_4b
f2b =~ c(0,1)*Relatedness_PE_1b + c(0,NA)*Relatedness_PE_2b + c(0,NA)*Relatedness_PE_3b
f3b =~ c(0,1)*Competence_PE_1b + c(0,NA)*Competence_PE_2b + c(0,NA)*Competence_PE_3b + c(0,N
A)*Competence_PE_4b
f1b ~~ c(0,NA)*f1b + c(0,0)*f1 + c(0,0)*f2 + c(0,0)*f3 + c(0,NA)*f2b + c(0,NA)*f3b
f2b ~~ c(0,NA)*f2b + c(0,0)*f1 + c(0,0)*f2 + c(0,0)*f3 + c(0,NA)*f3b
f3b ~~ c(0,NA)*f3b + c(0,0)*f1 + c(0,0)*f2 + c(0,0)*f3
'

# Fit hypothesized model
models$mod_3x3_fit <- cfa(mod_3x3, sample.cov = combined.cov, sample.nobs = combined.n, orth
ogonal = T)
```

```
## Warning in lav_object_post_check(object): lavaan WARNING: some estimated lv
## variances are negative
```

```
# summary
summary(models$mod_3x3_fit, fit.measures = T, standardized = T)
```

```
## lavaan 0.6-3 ended normally after 323 iterations
##
##   Optimization method                           NLMINB
##   Number of free parameters                         73
##   Number of equality constraints                    24
##
##   Number of observations per group
##   within                                           400
##   between                                           33
##
##   Estimator                                         ML
##   Model Fit Test Statistic                     189.720
##   Degrees of freedom                                83
##   P-value (Chi-square)                           0.000
##
## Chi-square for each group:
##
##   within                                       143.065
##   between                                       46.655
##
## Model test baseline model:
##
##   Minimum Function Test Statistic             2130.224
##   Degrees of freedom                               110
##   P-value                                        0.000
##
## User model versus baseline model:
##
##   Comparative Fit Index (CFI)                    0.947
##   Tucker-Lewis Index (TLI)                       0.930
##
## Loglikelihood and Information Criteria:
##
##   Loglikelihood user model (H0)              -5962.227
##   Loglikelihood unrestricted model (H1)      -5867.367
##
##   Number of free parameters                         49
##   Akaike (AIC)                               12022.453
##   Bayesian (BIC)                             12221.920
##   Sample-size adjusted Bayesian (BIC)        12066.421
##
## Root Mean Square Error of Approximation:
##
##   RMSEA                                          0.077
##   90 Percent Confidence Interval          0.063  0.092
##   P-value RMSEA <= 0.05                          0.001
##
## Standardized Root Mean Square Residual:
##
##   SRMR                                           0.118
```

```
## Warning in sqrt(ETA2): NaNs wurden erzeugt
```

```
## Warning in sqrt(ETA2): NaNs wurden erzeugt

## Warning in sqrt(ETA2): NaNs wurden erzeugt
```

```
##
## Parameter Estimates:
##
##   Information                                 Expected
##   Information saturated (h1) model          Structured
##   Standard Errors                             Standard
##
##
## Group 1 [within]:
##
## Latent Variables:
##                        Estimate  Std.Err  z-value  P(>|z|)   Std.lv
##   f1 =~
##     Atnmy_PE_1            1.000                               0.508
##     Atnmy_PE_2 (a)        1.533    0.145   10.582    0.000    0.778
##     Atnmy_PE_3 (b)        1.543    0.155    9.950    0.000    0.783
##     Atnmy_PE_4 (c)        1.791    0.159   11.250    0.000    0.909
##   f2 =~
##     Rltdn_PE_1            1.000                               0.606
##     Rltdn_PE_2 (d)        1.624    0.137   11.841    0.000    0.984
##     Rltdn_PE_3 (e)        1.389    0.117   11.918    0.000    0.842
##   f3 =~
##     Cmptn_PE_1            1.000                               1.000
##     Cmptn_PE_2 (g)        1.006    0.042   23.989    0.000    1.006
##     Cmptn_PE_3 (h)        0.757    0.041   18.271    0.000    0.757
##     Cmptn_PE_4 (i)        0.836    0.038   21.996    0.000    0.836
##   Autonomy_PE_1b =~
##     Atnmy_PE_1 (x)        1.000                               0.000
##   Autonomy_PE_2b =~
##     Atnmy_PE_2 (x)        1.000                               0.000
##   Autonomy_PE_3b =~
##     Atnmy_PE_3 (x)        1.000                               0.000
##   Autonomy_PE_4b =~
##     Atnmy_PE_4 (x)        1.000                               0.000
##   Relatedness_PE_1b =~
##     Rltdn_PE_1 (x)        1.000                               0.000
##   Relatedness_PE_2b =~
##     Rltdn_PE_2 (x)        1.000                               0.000
##   Relatedness_PE_3b =~
##     Rltdn_PE_3 (x)        1.000                               0.000
##   Competence_PE_1b =~
##     Cmptn_PE_1 (x)        1.000                               0.000
##   Competence_PE_2b =~
##     Cmptn_PE_2 (x)        1.000                               0.000
##   Competence_PE_3b =~
##     Cmptn_PE_3 (x)        1.000                               0.000
##   Competence_PE_4b =~
##     Cmptn_PE_4 (x)        1.000                               0.000
##   f1b =~
##     Atnmy_PE_1            0.000                                 NaN
##     Atnmy_PE_2            0.000                                 NaN
##     Atnmy_PE_3            0.000                                 NaN
##     Atnmy_PE_4            0.000                                 NaN
##   f2b =~
##     Rltdn_PE_1            0.000                                 NaN
##     Rltdn_PE_2            0.000                                 NaN
##     Rltdn_PE_3            0.000                                 NaN
##   f3b =~
##     Cmptn_PE_1            0.000                                 NaN
##     Cmptn_PE_2            0.000                                 NaN
```

```
##     Cmptn_PE_3            0.000                                      NaN
##     Cmptn_PE_4            0.000                                      NaN
##   Std.all
##
##     0.558
##     0.735
##     0.661
##     0.876
##
##     0.599
##     0.905
##     0.760
##
##     0.899
##     0.831
##     0.717
##     0.796
##
##     0.000
##
##     0.000
##
##     0.000
##
##     0.000
##
##     0.000
##
##     0.000
##
##     0.000
##
##     0.000
##
##     0.000
##
##     0.000
##
##     0.000
##
##       NaN
##       NaN
##       NaN
##       NaN
##
##       NaN
##       NaN
##       NaN
##
##       NaN
##       NaN
##       NaN
##       NaN
##
## Covariances:
##                    Estimate  Std.Err  z-value  P(>|z|)   Std.lv  Std.all
##   f1 ~~
##     f2         (y)    0.122    0.022    5.502    0.000    0.395    0.395
##     f3         (z)    0.326    0.035    9.260    0.000    0.643    0.643
##   f2 ~~
```

```
##     f3        (aa)     0.269    0.036    7.481    0.000    0.443    0.443
##   f1 ~~
##     f1b                0.000                               NaN      NaN
##   f2 ~~
##     f1b                0.000                               NaN      NaN
##   f3 ~~
##     f1b                0.000                               NaN      NaN
##   f1b ~~
##     f2b                0.000                               NaN      NaN
##     f3b                0.000                               NaN      NaN
##   f1 ~~
##     f2b                0.000                               NaN      NaN
##   f2 ~~
##     f2b                0.000                               NaN      NaN
##   f3 ~~
##     f2b                0.000                               NaN      NaN
##   f2b ~~
##     f3b                0.000                               NaN      NaN
##   f1 ~~
##     f3b                0.000                               NaN      NaN
##   f2 ~~
##     f3b                0.000                               NaN      NaN
##   f3 ~~
##     f3b                0.000                               NaN      NaN
##
## Variances:
##                    Estimate  Std.Err  z-value  P(>|z|)   Std.lv  Std.all
##    .Atnmy_PE_1 (j)     0.569    0.043   13.088    0.000    0.569    0.688
##    .Atnmy_PE_2 (k)     0.516    0.046   11.204    0.000    0.516    0.460
##    .Atnmy_PE_3 (l)     0.791    0.064   12.287    0.000    0.791    0.563
##    .Atnmy_PE_4 (m)     0.252    0.039    6.499    0.000    0.252    0.233
##    .Rltdn_PE_1 (n)     0.657    0.052   12.701    0.000    0.657    0.641
##    .Rltdn_PE_2 (o)     0.214    0.055    3.911    0.000    0.214    0.181
##    .Rltdn_PE_3 (p)     0.517    0.054    9.617    0.000    0.517    0.422
##    .Cmptn_PE_1 (r)     0.238    0.029    8.076    0.000    0.238    0.192
##    .Cmptn_PE_2 (s)     0.454    0.042   10.744    0.000    0.454    0.310
##    .Cmptn_PE_3 (t)     0.542    0.043   12.591    0.000    0.542    0.486
##    .Cmptn_PE_4 (u)     0.403    0.035   11.545    0.000    0.403    0.366
##     f1         (v)     0.258    0.045    5.744    0.000    1.000    1.000
##     f2         (w)     0.367    0.059    6.221    0.000    1.000    1.000
##     f3         (x)     1.000                               1.000    1.000
##     Atnmy_PE_1         0.000                               NaN      NaN
##     Atnmy_PE_2         0.000                               NaN      NaN
##     Atnmy_PE_3         0.000                               NaN      NaN
##     Atnmy_PE_4         0.000                               NaN      NaN
##     Rltdn_PE_1         0.000                               NaN      NaN
##     Rltdn_PE_2         0.000                               NaN      NaN
##     Rltdn_PE_3         0.000                               NaN      NaN
##     Cmptn_PE_1         0.000                               NaN      NaN
##     Cmptn_PE_2         0.000                               NaN      NaN
##     Cmptn_PE_3         0.000                               NaN      NaN
##     Cmptn_PE_4         0.000                               NaN      NaN
##     f1b                0.000                               NaN      NaN
##     f2b                0.000                               NaN      NaN
##     f3b                0.000                               NaN      NaN
##
##
## Group 2 [between]:
##
## Latent Variables:
```

```
##                          Estimate  Std.Err  z-value  P(>|z|)   Std.lv
##    f1 =~
##      Atn_PE_1               1.000                               0.508
##      Atn_PE_2    (a)        1.533    0.145   10.582    0.000    0.778
##      Atn_PE_3    (b)        1.543    0.155    9.950    0.000    0.783
##      Atn_PE_4    (c)        1.791    0.159   11.250    0.000    0.909
##    f2 =~
##      Rlt_PE_1               1.000                               0.606
##      Rlt_PE_2    (d)        1.624    0.137   11.841    0.000    0.984
##      Rlt_PE_3    (e)        1.389    0.117   11.918    0.000    0.842
##    f3 =~
##      Cmp_PE_1               1.000                               1.000
##      Cmp_PE_2    (g)        1.006    0.042   23.989    0.000    1.006
##      Cmp_PE_3    (h)        0.757    0.041   18.271    0.000    0.757
##      Cmp_PE_4    (i)        0.836    0.038   21.996    0.000    0.836
##    Autonomy_PE_1b =~
##      Atn_PE_1 (sqc)         1.000                               1.652
##    Autonomy_PE_2b =~
##      Atn_PE_2 (sqc)         1.000                               0.889
##    Autonomy_PE_3b =~
##      Atn_PE_3 (sqc)         1.000                               0.880
##    Autonomy_PE_4b =~
##      Atn_PE_4 (sqc)         1.000                               1.217
##    Relatedness_PE_1b =~
##      Rlt_PE_1 (sqc)         1.000                               0.083
##    Relatedness_PE_2b =~
##      Rlt_PE_2 (sqc)         1.000                                 NaN
##    Relatedness_PE_3b =~
##      Rlt_PE_3 (sqc)         1.000                                 NaN
##    Competence_PE_1b =~
##      Cmp_PE_1 (sqc)         1.000                               0.283
##    Competence_PE_2b =~
##      Cmp_PE_2 (sqc)         1.000                                 NaN
##    Competence_PE_3b =~
##      Cmp_PE_3 (sqc)         1.000                               0.495
##    Competence_PE_4b =~
##      Cmp_PE_4 (sqc)         1.000                               0.387
##    f1b =~
##      Atn_PE_1               1.000                               0.808
##      Atn_PE_2               0.741    0.179    4.131    0.000    1.113
##      Atn_PE_3               0.746    0.195    3.826    0.000    1.132
##      Atn_PE_4               0.866    0.207    4.187    0.000    0.950
##    f2b =~
##      Rlt_PE_1               1.000                                 NaN
##      Rlt_PE_2               0.754    0.233    3.241    0.001      NaN
##      Rlt_PE_3               0.693    0.227    3.058    0.002      NaN
##    f3b =~
##      Cmp_PE_1               1.000                                 NaN
##      Cmp_PE_2               2.606    3.097    0.842    0.400      NaN
##      Cmp_PE_3              33.347    0.444   75.092    0.000      NaN
##      Cmp_PE_4              -2.916    5.371   -0.543    0.587      NaN
##    Std.all
##
##      0.269
##      0.563
##      0.531
##      0.568
##
##      0.597
##      0.955
```

```
##     0.852
##
##     0.871
##     0.840
##     0.649
##     0.747
##
##     0.876
##
##     0.643
##
##     0.596
##
##     0.761
##
##     0.082
##
##       NaN
##
##       NaN
##
##     0.247
##
##       NaN
##
##     0.425
##
##     0.346
##
##     0.808
##     1.113
##     1.132
##     0.950
##
##       NaN
##       NaN
##       NaN
##
##       NaN
##       NaN
##       NaN
##       NaN
##
## Covariances:
##                    Estimate  Std.Err  z-value  P(>|z|)   Std.lv  Std.all
##   f1 ~~
##     f2         (y)    0.122    0.022    5.502    0.000    0.395    0.395
##     f3         (z)    0.326    0.035    9.260    0.000    0.643    0.643
##   f2 ~~
##     f3        (aa)    0.269    0.036    7.481    0.000    0.443    0.443
##   f1 ~~
##     f1b               0.000                               0.000    0.000
##   f2 ~~
##     f1b               0.000                               0.000    0.000
##   f3 ~~
##     f1b               0.000                               0.000    0.000
##   f1b ~~
##     f2b               0.044    0.258    0.172    0.864    0.139    0.139
##     f3b               0.016    0.008    1.944    0.052    0.357    0.357
##   f1 ~~
```

```
##     f2b               0.000                               0.000    0.000
##   f2 ~~
##     f2b               0.000                               0.000    0.000
##   f3 ~~
##     f2b               0.000                               0.000    0.000
##   f2b ~~
##     f3b               0.014    0.005    2.705    0.007    1.742    1.742
##   f1 ~~
##     f3b               0.000                               0.000    0.000
##   f2 ~~
##     f3b               0.000                               0.000    0.000
##   f3 ~~
##     f3b               0.000                               0.000    0.000
##
## Variances:
##                    Estimate  Std.Err  z-value  P(>|z|)   Std.lv  Std.all
##    .Atnmy_PE_1 (j)    0.569    0.043   13.088    0.000    0.569    0.160
##    .Atnmy_PE_2 (k)    0.516    0.046   11.204    0.000    0.516    0.270
##    .Atnmy_PE_3 (l)    0.791    0.064   12.287    0.000    0.791    0.363
##    .Atnmy_PE_4 (m)    0.252    0.039    6.499    0.000    0.252    0.098
##    .Rltdn_PE_1 (n)    0.657    0.052   12.701    0.000    0.657    0.637
##    .Rltdn_PE_2 (o)    0.214    0.055    3.911    0.000    0.214    0.202
##    .Rltdn_PE_3 (p)    0.517    0.054    9.617    0.000    0.517    0.529
##    .Cmptn_PE_1 (r)    0.238    0.029    8.076    0.000    0.238    0.181
##    .Cmptn_PE_2 (s)    0.454    0.042   10.744    0.000    0.454    0.317
##    .Cmptn_PE_3 (t)    0.542    0.043   12.591    0.000    0.542    0.398
##    .Cmptn_PE_4 (u)    0.403    0.035   11.545    0.000    0.403    0.322
##     f1         (v)    0.258    0.045    5.744    0.000    1.000    1.000
##     f2         (w)    0.367    0.059    6.221    0.000    1.000    1.000
##     f3         (x)    1.000                               1.000    1.000
##     Atnmy_PE_1        0.948    0.461    2.054    0.040    0.347    0.347
##     Atnmy_PE_2       -0.189    0.131   -1.445    0.149   -0.239   -0.239
##     Atnmy_PE_3       -0.217    0.186   -1.168    0.243   -0.280   -0.280
##     Atnmy_PE_4        0.144    0.162    0.887    0.375    0.097    0.097
##     Rltdn_PE_1        0.064    0.215    0.296    0.767    9.281    9.281
##     Rltdn_PE_2       -0.088    0.109   -0.810    0.418      NaN      NaN
##     Rltdn_PE_3       -0.222    0.114   -1.941    0.052      NaN      NaN
##     Cmptn_PE_1        0.081    0.133    0.610    0.542    1.015    1.015
##     Cmptn_PE_2       -0.026    0.160   -0.160    0.873      NaN      NaN
##     Cmptn_PE_3        1.557    2.547    0.611    0.541    6.347    6.347
##     Cmptn_PE_4        0.160    0.176    0.911    0.363    1.067    1.067
##     f1b               1.782    0.840    2.121    0.034    1.000    1.000
##     f2b              -0.057    0.243   -0.234    0.815      NaN      NaN
##     f3b              -0.001    0.002   -0.521    0.602      NaN      NaN
```

Path diagramm

```
semPlot::semPaths(models$mod_3x3_fit,"std",edge.label.cex=0.5, curvePivot = TRUE)
```

```
## Warning in sqrt(ETA2): NaNs wurden erzeugt

## Warning in sqrt(ETA2): NaNs wurden erzeugt

## Warning in sqrt(ETA2): NaNs wurden erzeugt
```

```
## Warning in qgraph::qgraph(Edgelist, labels = nLab, bidirectional = Bidir, :
## Non-finite weights are omitted

## Warning in qgraph::qgraph(Edgelist, labels = nLab, bidirectional = Bidir, :
## Non-finite weights are omitted
```

1

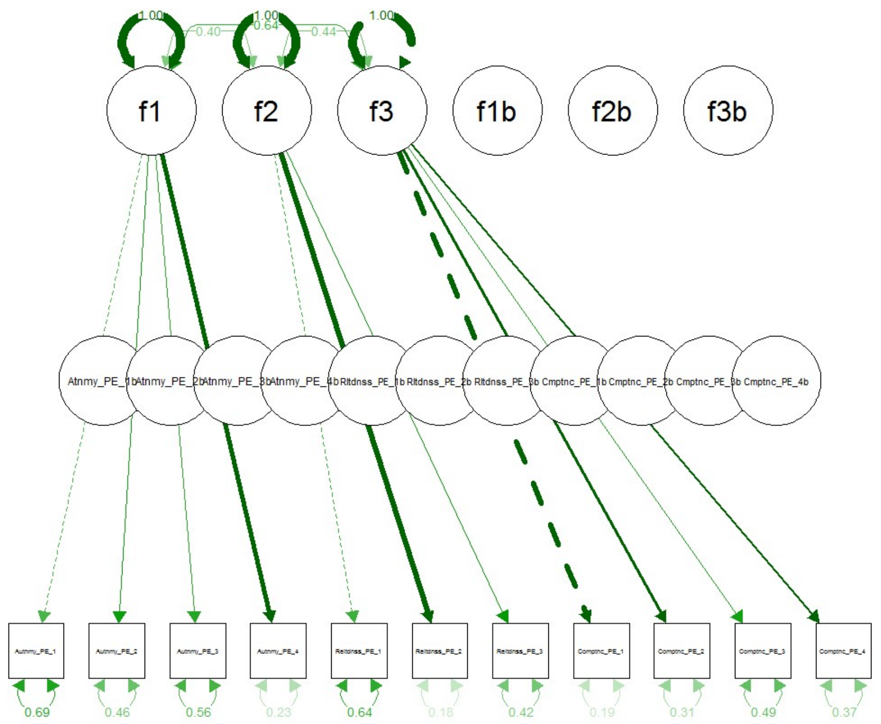

2

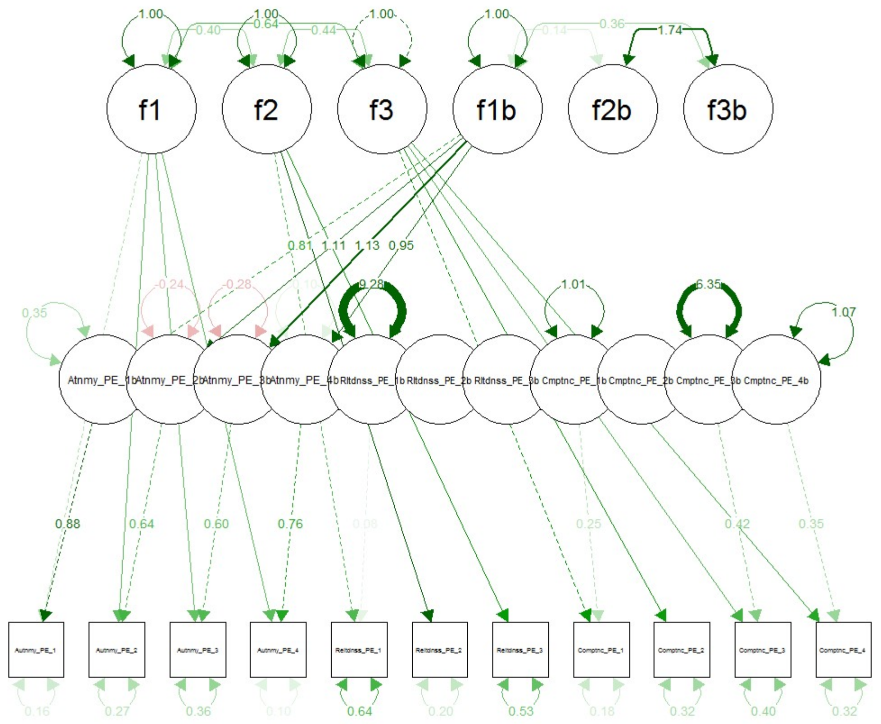

3x3, Variances set to 0 where necessary at A2, A3, R2, R3

Huang & Cornell, 2016: The presence of small negative residual variance estimates at the school level required residual variances to be fixed to zero for five items (see Table 1). Fixing residual variances at the group level to zero is often done in MCFA and is recommended to allow the model to converge…

```
mod_3x3_var <- '
f1 =~ Autonomy_PE_1 + c(a,a)*Autonomy_PE_2 + c(b,b)*Autonomy_PE_3 + c(c,c)*Autonomy_PE_4
f2 =~ Relatedness_PE_1 + c(d,d)*Relatedness_PE_2 + c(e,e)*Relatedness_PE_3
f3 =~ Competence_PE_1 + c(g,g)*Competence_PE_2 + c(h,h)*Competence_PE_3 + c(i,i)*Competence_
PE_4
Autonomy_PE_1 ~~ c(j,j)*Autonomy_PE_1
Autonomy_PE_2 ~~ c(NA,0)*Autonomy_PE_2
Autonomy_PE_3 ~~ c(NA,0)*Autonomy_PE_3
Autonomy_PE_4 ~~ c(m,m)*Autonomy_PE_4
Relatedness_PE_1 ~~ c(n,n)*Relatedness_PE_1
Relatedness_PE_2 ~~ c(NA,0)*Relatedness_PE_2
Relatedness_PE_3 ~~ c(NA,0)*Relatedness_PE_3
Competence_PE_1 ~~ c(r,r)*Competence_PE_1
Competence_PE_2 ~~ c(NA,0)*Competence_PE_2
Competence_PE_3 ~~ c(t,t)*Competence_PE_3
Competence_PE_4 ~~ c(u,u)*Competence_PE_4
f1 ~~ c(v,v)*f1
f2 ~~ c(w,w)*f2
f3 ~~ c(x,x)*f3
f1 ~~ c(y,y)*f2
f1 ~~ c(z,z)*f3
f2 ~~ c(aa,aa)*f3
Autonomy_PE_1b =~ c(0,x$sqc)*Autonomy_PE_1
Autonomy_PE_1b ~~ c(0,NA)*Autonomy_PE_1b
Autonomy_PE_2b =~ c(0,x$sqc)*Autonomy_PE_2
Autonomy_PE_2b ~~ c(0,NA)*Autonomy_PE_2b
Autonomy_PE_3b =~ c(0,x$sqc)*Autonomy_PE_3
Autonomy_PE_3b ~~ c(0,NA)*Autonomy_PE_3b
Autonomy_PE_4b =~ c(0,x$sqc)*Autonomy_PE_4
Autonomy_PE_4b ~~ c(0,NA)*Autonomy_PE_4b
Relatedness_PE_1b =~ c(0,x$sqc)*Relatedness_PE_1
Relatedness_PE_1b ~~ c(0,NA)*Relatedness_PE_1b
Relatedness_PE_2b =~ c(0,x$sqc)*Relatedness_PE_2
Relatedness_PE_2b ~~ c(0,NA)*Relatedness_PE_2b
Relatedness_PE_3b =~ c(0,x$sqc)*Relatedness_PE_3
Relatedness_PE_3b ~~ c(0,NA)*Relatedness_PE_3b
Competence_PE_1b =~ c(0,x$sqc)*Competence_PE_1
Competence_PE_1b ~~ c(0,NA)*Competence_PE_1b
Competence_PE_2b =~ c(0,x$sqc)*Competence_PE_2
Competence_PE_2b ~~ c(0,NA)*Competence_PE_2b
Competence_PE_3b =~ c(0,x$sqc)*Competence_PE_3
Competence_PE_3b ~~ c(0,NA)*Competence_PE_3b
Competence_PE_4b =~ c(0,x$sqc)*Competence_PE_4
Competence_PE_4b ~~ c(0,NA)*Competence_PE_4b
f1b =~ c(0,1)*Autonomy_PE_1b + c(0,NA)*Autonomy_PE_2b + c(0,NA)*Autonomy_PE_3b + c(0,NA)*Aut
onomy_PE_4b
f2b =~ c(0,1)*Relatedness_PE_1b + c(0,NA)*Relatedness_PE_2b + c(0,NA)*Relatedness_PE_3b
f3b =~ c(0,1)*Competence_PE_1b + c(0,NA)*Competence_PE_2b + c(0,NA)*Competence_PE_3b + c(0,N
A)*Competence_PE_4b
f1b ~~ c(0,NA)*f1b + c(0,0)*f1 + c(0,0)*f2 + c(0,0)*f3 + c(0,NA)*f2b + c(0,NA)*f3b
f2b ~~ c(0,NA)*f2b + c(0,0)*f1 + c(0,0)*f2 + c(0,0)*f3 + c(0,NA)*f3b
f3b ~~ c(0,NA)*f3b + c(0,0)*f1 + c(0,0)*f2 + c(0,0)*f3
'
# fit hypothesized model
models$mod_3x3_var_fit <- cfa(mod_3x3_var, sample.cov = combined.cov, sample.nobs = combine
d.n, orthogonal = T)
```

```
## Warning in lav_object_post_check(object): lavaan WARNING: some estimated lv
## variances are negative
```

```
summary(models$mod_3x3_var_fit, fit.measures = T, standardized = T)
```

```
## lavaan 0.6-3 ended normally after 312 iterations
##
##   Optimization method                           NLMINB
##   Number of free parameters                         68
##   Number of equality constraints                    19
##
##   Number of observations per group
##   within                                           400
##   between                                           33
##
##   Estimator                                         ML
##   Model Fit Test Statistic                     189.720
##   Degrees of freedom                                83
##   P-value (Chi-square)                           0.000
##
## Chi-square for each group:
##
##   within                                       143.065
##   between                                       46.655
##
## Model test baseline model:
##
##   Minimum Function Test Statistic             2130.224
##   Degrees of freedom                               110
##   P-value                                        0.000
##
## User model versus baseline model:
##
##   Comparative Fit Index (CFI)                    0.947
##   Tucker-Lewis Index (TLI)                       0.930
##
## Loglikelihood and Information Criteria:
##
##   Loglikelihood user model (H0)              -5962.227
##   Loglikelihood unrestricted model (H1)      -5867.367
##
##   Number of free parameters                         49
##   Akaike (AIC)                               12022.453
##   Bayesian (BIC)                             12221.920
##   Sample-size adjusted Bayesian (BIC)        12066.421
##
## Root Mean Square Error of Approximation:
##
##   RMSEA                                          0.077
##   90 Percent Confidence Interval          0.063  0.092
##   P-value RMSEA <= 0.05                          0.001
##
## Standardized Root Mean Square Residual:
##
##   SRMR                                           0.118
```

```
## Warning in sqrt(ETA2): NaNs wurden erzeugt
```

```
## Warning in sqrt(ETA2): NaNs wurden erzeugt

## Warning in sqrt(ETA2): NaNs wurden erzeugt
```

```
##
## Parameter Estimates:
##
##   Information                                 Expected
##   Information saturated (h1) model          Structured
##   Standard Errors                             Standard
##
##
## Group 1 [within]:
##
## Latent Variables:
##                         Estimate  Std.Err  z-value  P(>|z|)   Std.lv
##   f1 =~
##     Atnmy_PE_1             1.000                                0.508
##     Atnmy_PE_2 (a)         1.533    0.145   10.582    0.000    0.778
##     Atnmy_PE_3 (b)         1.543    0.155    9.950    0.000    0.783
##     Atnmy_PE_4 (c)         1.791    0.159   11.250    0.000    0.909
##   f2 =~
##     Rltdn_PE_1             1.000                                0.606
##     Rltdn_PE_2 (d)         1.624    0.137   11.841    0.000    0.984
##     Rltdn_PE_3 (e)         1.389    0.117   11.918    0.000    0.842
##   f3 =~
##     Cmptn_PE_1             1.000                                1.000
##     Cmptn_PE_2 (g)         1.006    0.042   23.989    0.000    1.006
##     Cmptn_PE_3 (h)         0.757    0.041   18.271    0.000    0.757
##     Cmptn_PE_4 (i)         0.836    0.038   21.996    0.000    0.836
##   Autonomy_PE_1b =~
##     Atnmy_PE_1 (x)         1.000                                0.000
##   Autonomy_PE_2b =~
##     Atnmy_PE_2 (x)         1.000                                0.000
##   Autonomy_PE_3b =~
##     Atnmy_PE_3 (x)         1.000                                0.000
##   Autonomy_PE_4b =~
##     Atnmy_PE_4 (x)         1.000                                0.000
##   Relatedness_PE_1b =~
##     Rltdn_PE_1 (x)         1.000                                0.000
##   Relatedness_PE_2b =~
##     Rltdn_PE_2 (x)         1.000                                0.000
##   Relatedness_PE_3b =~
##     Rltdn_PE_3 (x)         1.000                                0.000
##   Competence_PE_1b =~
##     Cmptn_PE_1 (x)         1.000                                0.000
##   Competence_PE_2b =~
##     Cmptn_PE_2 (x)         1.000                                0.000
##   Competence_PE_3b =~
##     Cmptn_PE_3 (x)         1.000                                0.000
##   Competence_PE_4b =~
##     Cmptn_PE_4 (x)         1.000                                0.000
##   f1b =~
##     Atnmy_PE_1             0.000                                  NaN
##     Atnmy_PE_2             0.000                                  NaN
##     Atnmy_PE_3             0.000                                  NaN
##     Atnmy_PE_4             0.000                                  NaN
##   f2b =~
##     Rltdn_PE_1             0.000                                  NaN
##     Rltdn_PE_2             0.000                                  NaN
##     Rltdn_PE_3             0.000                                  NaN
##   f3b =~
##     Cmptn_PE_1             0.000                                  NaN
##     Cmptn_PE_2             0.000                                  NaN
```

```
##     Cmptn_PE_3            0.000                                  NaN
##     Cmptn_PE_4            0.000                                  NaN
##   Std.all
##
##     0.558
##     0.735
##     0.661
##     0.876
##
##     0.599
##     0.905
##     0.760
##
##     0.899
##     0.831
##     0.717
##     0.796
##
##     0.000
##
##     0.000
##
##     0.000
##
##     0.000
##
##     0.000
##
##     0.000
##
##     0.000
##
##     0.000
##
##     0.000
##
##     0.000
##
##     0.000
##
##       NaN
##       NaN
##       NaN
##       NaN
##
##       NaN
##       NaN
##       NaN
##
##       NaN
##       NaN
##       NaN
##       NaN
##
## Covariances:
##                    Estimate  Std.Err  z-value  P(>|z|)   Std.lv  Std.all
##   f1 ~~
##     f2         (y)    0.122    0.022    5.502    0.000    0.395    0.395
##     f3         (z)    0.326    0.035    9.260    0.000    0.643    0.643
##   f2 ~~
```

```
##     f3        (aa)    0.269    0.036    7.481    0.000    0.443    0.443
##   f1 ~~
##     f1b               0.000                               NaN      NaN
##   f2 ~~
##     f1b               0.000                               NaN      NaN
##   f3 ~~
##     f1b               0.000                               NaN      NaN
##   f1b ~~
##     f2b               0.000                               NaN      NaN
##     f3b               0.000                               NaN      NaN
##   f1 ~~
##     f2b               0.000                               NaN      NaN
##   f2 ~~
##     f2b               0.000                               NaN      NaN
##   f3 ~~
##     f2b               0.000                               NaN      NaN
##   f2b ~~
##     f3b               0.000                               NaN      NaN
##   f1 ~~
##     f3b               0.000                               NaN      NaN
##   f2 ~~
##     f3b               0.000                               NaN      NaN
##   f3 ~~
##     f3b               0.000                               NaN      NaN
##
## Variances:
##                    Estimate  Std.Err  z-value  P(>|z|)   Std.lv  Std.all
##    .Atnmy_PE_1 (j)    0.569    0.043   13.088    0.000    0.569    0.688
##    .Atnmy_PE_2        0.516    0.046   11.204    0.000    0.516    0.460
##    .Atnmy_PE_3        0.791    0.064   12.287    0.000    0.791    0.563
##    .Atnmy_PE_4 (m)    0.252    0.039    6.499    0.000    0.252    0.233
##    .Rltdn_PE_1 (n)    0.657    0.052   12.701    0.000    0.657    0.641
##    .Rltdn_PE_2        0.214    0.055    3.911    0.000    0.214    0.181
##    .Rltdn_PE_3        0.517    0.054    9.617    0.000    0.517    0.422
##    .Cmptn_PE_1 (r)    0.238    0.029    8.076    0.000    0.238    0.192
##    .Cmptn_PE_2        0.454    0.042   10.744    0.000    0.454    0.310
##    .Cmptn_PE_3 (t)    0.542    0.043   12.591    0.000    0.542    0.486
##    .Cmptn_PE_4 (u)    0.403    0.035   11.545    0.000    0.403    0.366
##     f1         (v)    0.258    0.045    5.744    0.000    1.000    1.000
##     f2         (w)    0.367    0.059    6.221    0.000    1.000    1.000
##     f3         (x)    1.000                               1.000    1.000
##     Atnmy_PE_1        0.000                               NaN      NaN
##     Atnmy_PE_2        0.000                               NaN      NaN
##     Atnmy_PE_3        0.000                               NaN      NaN
##     Atnmy_PE_4        0.000                               NaN      NaN
##     Rltdn_PE_1        0.000                               NaN      NaN
##     Rltdn_PE_2        0.000                               NaN      NaN
##     Rltdn_PE_3        0.000                               NaN      NaN
##     Cmptn_PE_1        0.000                               NaN      NaN
##     Cmptn_PE_2        0.000                               NaN      NaN
##     Cmptn_PE_3        0.000                               NaN      NaN
##     Cmptn_PE_4        0.000                               NaN      NaN
##     f1b               0.000                               NaN      NaN
##     f2b               0.000                               NaN      NaN
##     f3b               0.000                               NaN      NaN
##
##
## Group 2 [between]:
##
## Latent Variables:
```

```
##                          Estimate  Std.Err  z-value  P(>|z|)   Std.lv
##    f1 =~
##      Atn_PE_1               1.000                               0.508
##      Atn_PE_2    (a)        1.533    0.145   10.582    0.000    0.778
##      Atn_PE_3    (b)        1.543    0.155    9.950    0.000    0.783
##      Atn_PE_4    (c)        1.791    0.159   11.250    0.000    0.909
##    f2 =~
##      Rlt_PE_1               1.000                               0.606
##      Rlt_PE_2    (d)        1.624    0.137   11.841    0.000    0.984
##      Rlt_PE_3    (e)        1.389    0.117   11.918    0.000    0.842
##    f3 =~
##      Cmp_PE_1               1.000                               1.000
##      Cmp_PE_2    (g)        1.006    0.042   23.989    0.000    1.006
##      Cmp_PE_3    (h)        0.757    0.041   18.271    0.000    0.757
##      Cmp_PE_4    (i)        0.836    0.038   21.996    0.000    0.836
##    Autonomy_PE_1b =~
##      Atn_PE_1 (sqc)         1.000                               1.652
##    Autonomy_PE_2b =~
##      Atn_PE_2 (sqc)         1.000                               1.143
##    Autonomy_PE_3b =~
##      Atn_PE_3 (sqc)         1.000                               1.251
##    Autonomy_PE_4b =~
##      Atn_PE_4 (sqc)         1.000                               1.217
##    Relatedness_PE_1b =~
##      Rlt_PE_1 (sqc)         1.000                               0.083
##    Relatedness_PE_2b =~
##      Rlt_PE_2 (sqc)         1.000                               0.306
##    Relatedness_PE_3b =~
##      Rlt_PE_3 (sqc)         1.000                               0.518
##    Competence_PE_1b =~
##      Cmp_PE_1 (sqc)         1.000                               0.283
##    Competence_PE_2b =~
##      Cmp_PE_2 (sqc)         1.000                               0.649
##    Competence_PE_3b =~
##      Cmp_PE_3 (sqc)         1.000                               0.495
##    Competence_PE_4b =~
##      Cmp_PE_4 (sqc)         1.000                               0.387
##    f1b =~
##      Atn_PE_1               1.000                               0.808
##      Atn_PE_2               0.741    0.179    4.131    0.000    0.866
##      Atn_PE_3               0.746    0.195    3.826    0.000    0.796
##      Atn_PE_4               0.866    0.207    4.187    0.000    0.950
##    f2b =~
##      Rlt_PE_1               1.000                                 NaN
##      Rlt_PE_2               0.754    0.233    3.241    0.001      NaN
##      Rlt_PE_3               0.693    0.227    3.058    0.002      NaN
##    f3b =~
##      Cmp_PE_1               1.000                                 NaN
##      Cmp_PE_2               2.606    3.096    0.842    0.400      NaN
##      Cmp_PE_3              33.345    0.444   75.085    0.000      NaN
##      Cmp_PE_4              -2.916    5.371   -0.543    0.587      NaN
##    Std.all
##
##      0.269
##      0.563
##      0.531
##      0.568
##
##      0.597
##      0.955
```

```
##     0.852
##
##     0.871
##     0.840
##     0.649
##     0.747
##
##     0.876
##
##     0.827
##
##     0.848
##
##     0.761
##
##     0.082
##
##     0.297
##
##     0.524
##
##     0.247
##
##     0.542
##
##     0.425
##
##     0.346
##
##     0.808
##     0.866
##     0.796
##     0.950
##
##       NaN
##       NaN
##       NaN
##
##       NaN
##       NaN
##       NaN
##       NaN
##
## Covariances:
##                    Estimate  Std.Err  z-value  P(>|z|)   Std.lv  Std.all
##   f1 ~~
##     f2         (y)    0.122    0.022    5.502    0.000    0.395    0.395
##     f3         (z)    0.326    0.035    9.260    0.000    0.643    0.643
##   f2 ~~
##     f3        (aa)    0.269    0.036    7.481    0.000    0.443    0.443
##   f1 ~~
##     f1b               0.000                               0.000    0.000
##   f2 ~~
##     f1b               0.000                               0.000    0.000
##   f3 ~~
##     f1b               0.000                               0.000    0.000
##   f1b ~~
##     f2b               0.044    0.258    0.172    0.864    0.139    0.139
##     f3b               0.016    0.008    1.944    0.052    0.357    0.357
##   f1 ~~
```

```
##     f2b               0.000                               0.000    0.000
##   f2 ~~
##     f2b               0.000                               0.000    0.000
##   f3 ~~
##     f2b               0.000                               0.000    0.000
##   f2b ~~
##     f3b               0.014    0.005    2.705    0.007    1.742    1.742
##   f1 ~~
##     f3b               0.000                               0.000    0.000
##   f2 ~~
##     f3b               0.000                               0.000    0.000
##   f3 ~~
##     f3b               0.000                               0.000    0.000
##
## Variances:
##                    Estimate  Std.Err  z-value  P(>|z|)   Std.lv  Std.all
##    .Atnmy_PE_1 (j)    0.569    0.043   13.088    0.000    0.569    0.160
##    .Atnmy_PE_2        0.000                               0.000    0.000
##    .Atnmy_PE_3        0.000                               0.000    0.000
##    .Atnmy_PE_4 (m)    0.252    0.039    6.499    0.000    0.252    0.098
##    .Rltdn_PE_1 (n)    0.657    0.052   12.701    0.000    0.657    0.637
##    .Rltdn_PE_2        0.000                               0.000    0.000
##    .Rltdn_PE_3        0.000                               0.000    0.000
##    .Cmptn_PE_1 (r)    0.238    0.029    8.076    0.000    0.238    0.181
##    .Cmptn_PE_2        0.000                               0.000    0.000
##    .Cmptn_PE_3 (t)    0.542    0.043   12.591    0.000    0.542    0.398
##    .Cmptn_PE_4 (u)    0.403    0.035   11.545    0.000    0.403    0.322
##     f1         (v)    0.258    0.045    5.744    0.000    1.000    1.000
##     f2         (w)    0.367    0.059    6.221    0.000    1.000    1.000
##     f3         (x)    1.000                               1.000    1.000
##     Atnmy_PE_1        0.948    0.461    2.054    0.040    0.347    0.347
##     Atnmy_PE_2        0.328    0.123    2.665    0.008    0.251    0.251
##     Atnmy_PE_3        0.574    0.175    3.278    0.001    0.366    0.366
##     Atnmy_PE_4        0.144    0.162    0.887    0.375    0.097    0.097
##     Rltdn_PE_1        0.064    0.215    0.296    0.767    9.278    9.278
##     Rltdn_PE_2        0.126    0.109    1.160    0.246    1.345    1.345
##     Rltdn_PE_3        0.295    0.108    2.744    0.006    1.102    1.102
##     Cmptn_PE_1        0.081    0.133    0.610    0.542    1.015    1.015
##     Cmptn_PE_2        0.429    0.155    2.758    0.006    1.019    1.019
##     Cmptn_PE_3        1.557    2.547    0.611    0.541    6.347    6.347
##     Cmptn_PE_4        0.160    0.176    0.911    0.363    1.067    1.067
##     f1b               1.782    0.840    2.121    0.034    1.000    1.000
##     f2b              -0.057    0.243   -0.234    0.815      NaN      NaN
##     f3b              -0.001    0.002   -0.521    0.602      NaN      NaN
```

Path diagramm

```
semPlot::semPaths(models$mod_3x3_var_fit,"std",edge.label.cex=0.5, curvePivot = TRUE)
```

```
## Warning in sqrt(ETA2): NaNs wurden erzeugt

## Warning in sqrt(ETA2): NaNs wurden erzeugt

## Warning in sqrt(ETA2): NaNs wurden erzeugt
```

```
## Warning in qgraph::qgraph(Edgelist, labels = nLab, bidirectional = Bidir, :
## Non-finite weights are omitted

## Warning in qgraph::qgraph(Edgelist, labels = nLab, bidirectional = Bidir, :
## Non-finite weights are omitted
```

1

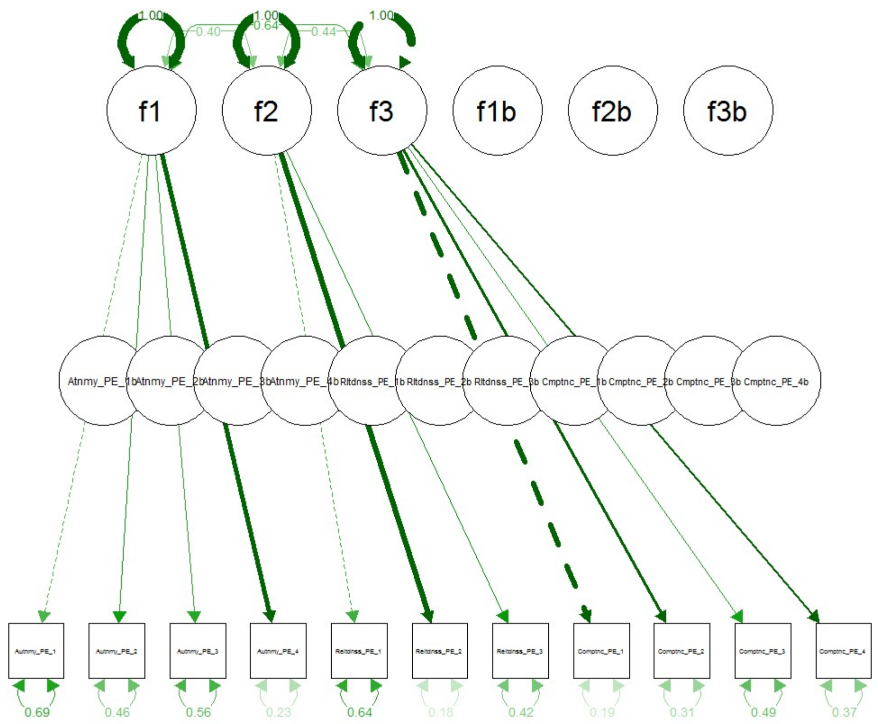

2

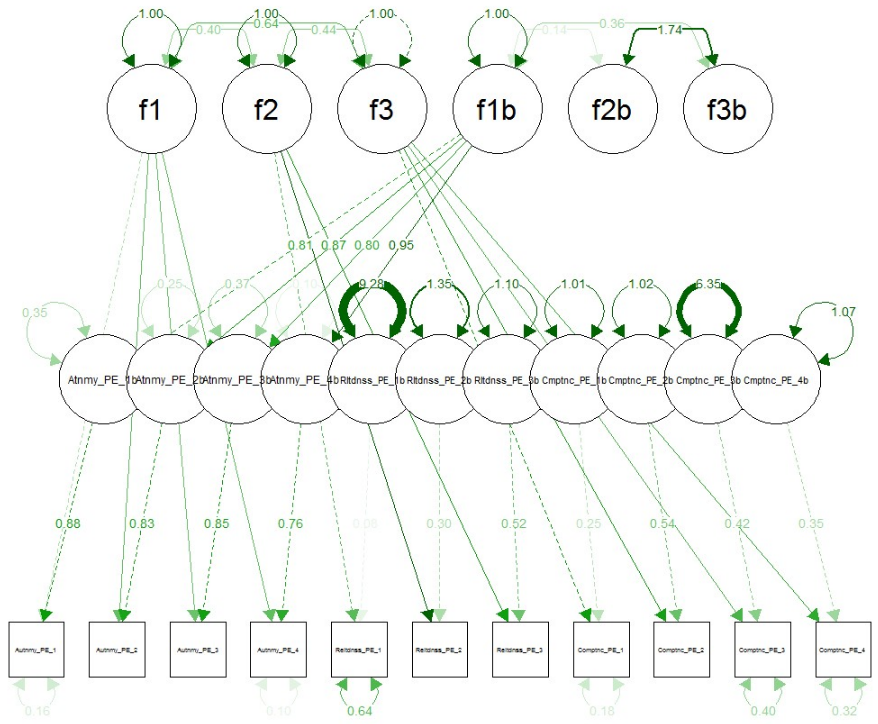

3x3, freely estimated loadings, variances and covariances

model probably not identifiable

3x3, freely estimated loadings and latent factor variances and covariances, manifest variances set equal

```r
mod_3x3_free_vareq <- '
f1 =~ Autonomy_PE_1 + Autonomy_PE_2 + Autonomy_PE_3 + Autonomy_PE_4
f2 =~ Relatedness_PE_1 + Relatedness_PE_2 + Relatedness_PE_3
f3 =~ Competence_PE_1 + Competence_PE_2 + Competence_PE_3 + Competence_PE_4
Autonomy_PE_1 ~~ c(a,a)*Autonomy_PE_1
Autonomy_PE_2 ~~ c(b,b)*Autonomy_PE_2
Autonomy_PE_3 ~~ c(c,c)*Autonomy_PE_3
Autonomy_PE_4 ~~ c(d,d)*Autonomy_PE_4
Relatedness_PE_1 ~~ c(e,e)*Relatedness_PE_1
Relatedness_PE_2 ~~ c(f,f)*Relatedness_PE_2
Relatedness_PE_3 ~~ c(g,g)*Relatedness_PE_3
Competence_PE_1 ~~ c(i,i)*Competence_PE_1
Competence_PE_2 ~~ c(j,j)*Competence_PE_2
Competence_PE_3 ~~ c(k,k)*Competence_PE_3
Competence_PE_4 ~~ c(l,l)*Competence_PE_4
f1 ~~ f1
f2 ~~ f2
f3 ~~ f3
f1 ~~ f2
f1 ~~ f3
f2 ~~ f3
Autonomy_PE_1b =~ c(0,x$sqc)*Autonomy_PE_1
Autonomy_PE_1b ~~ c(0,NA)*Autonomy_PE_1b
Autonomy_PE_2b =~ c(0,x$sqc)*Autonomy_PE_2
Autonomy_PE_2b ~~ c(0,NA)*Autonomy_PE_2b
Autonomy_PE_3b =~ c(0,x$sqc)*Autonomy_PE_3
Autonomy_PE_3b ~~ c(0,NA)*Autonomy_PE_3b
Autonomy_PE_4b =~ c(0,x$sqc)*Autonomy_PE_4
Autonomy_PE_4b ~~ c(0,NA)*Autonomy_PE_4b
Relatedness_PE_1b =~ c(0,x$sqc)*Relatedness_PE_1
Relatedness_PE_1b ~~ c(0,NA)*Relatedness_PE_1b
Relatedness_PE_2b =~ c(0,x$sqc)*Relatedness_PE_2
Relatedness_PE_2b ~~ c(0,NA)*Relatedness_PE_2b
Relatedness_PE_3b =~ c(0,x$sqc)*Relatedness_PE_3
Relatedness_PE_3b ~~ c(0,NA)*Relatedness_PE_3b
Competence_PE_1b =~ c(0,x$sqc)*Competence_PE_1
Competence_PE_1b ~~ c(0,NA)*Competence_PE_1b
Competence_PE_2b =~ c(0,x$sqc)*Competence_PE_2
Competence_PE_2b ~~ c(0,NA)*Competence_PE_2b
Competence_PE_3b =~ c(0,x$sqc)*Competence_PE_3
Competence_PE_3b ~~ c(0,NA)*Competence_PE_3b
Competence_PE_4b =~ c(0,x$sqc)*Competence_PE_4
Competence_PE_4b ~~ c(0,NA)*Competence_PE_4b
f1b =~ c(0,1)*Autonomy_PE_1b + c(0,NA)*Autonomy_PE_2b + c(0,NA)*Autonomy_PE_3b + c(0,NA)*Aut
onomy_PE_4b
f2b =~ c(0,1)*Relatedness_PE_1b + c(0,NA)*Relatedness_PE_2b + c(0,NA)*Relatedness_PE_3b
f3b =~ c(0,1)*Competence_PE_1b + c(0,NA)*Competence_PE_2b + c(0,NA)*Competence_PE_3b + c(0,N
A)*Competence_PE_4b
f1b ~~ c(0,NA)*f1b + c(0,0)*f1 + c(0,0)*f2 + c(0,0)*f3 + c(0,NA)*f2b + c(0,NA)*f3b
f2b ~~ c(0,NA)*f2b + c(0,0)*f1 + c(0,0)*f2 + c(0,0)*f3 + c(0,NA)*f3b
f3b ~~ c(0,NA)*f3b + c(0,0)*f1 + c(0,0)*f2 + c(0,0)*f3
'
# Fit hypothesized model

models$mod_3x3_free_vareq_fit <- cfa(mod_3x3_free_vareq, sample.cov = combined.cov, sample.n
obs = combined.n, orthogonal = T)
```

```
## Warning in lav_object_post_check(object): lavaan WARNING: some estimated lv
## variances are negative
```

```
summary(models$mod_3x3_free_vareq_fit, fit.measures = T, standardized = T)
```

```
## lavaan 0.6-3 ended normally after 477 iterations
##
##   Optimization method                           NLMINB
##   Number of free parameters                         75
##   Number of equality constraints                    11
##
##   Number of observations per group
##   within                                           400
##   between                                           33
##
##   Estimator                                         ML
##   Model Fit Test Statistic                     146.746
##   Degrees of freedom                                68
##   P-value (Chi-square)                           0.000
##
## Chi-square for each group:
##
##   within                                       124.159
##   between                                       22.587
##
## Model test baseline model:
##
##   Minimum Function Test Statistic             2130.224
##   Degrees of freedom                               110
##   P-value                                        0.000
##
## User model versus baseline model:
##
##   Comparative Fit Index (CFI)                    0.961
##   Tucker-Lewis Index (TLI)                       0.937
##
## Loglikelihood and Information Criteria:
##
##   Loglikelihood user model (H0)              -5940.740
##   Loglikelihood unrestricted model (H1)      -5867.367
##
##   Number of free parameters                         64
##   Akaike (AIC)                               12009.480
##   Bayesian (BIC)                             12270.007
##   Sample-size adjusted Bayesian (BIC)        12066.907
##
## Root Mean Square Error of Approximation:
##
##   RMSEA                                          0.073
##   90 Percent Confidence Interval          0.057  0.089
##   P-value RMSEA <= 0.05                          0.011
##
## Standardized Root Mean Square Residual:
##
##   SRMR                                           0.056
```

```
## Warning in sqrt(ETA2): NaNs wurden erzeugt
```

```
## Warning in sqrt(ETA2): NaNs wurden erzeugt

## Warning in sqrt(ETA2): NaNs wurden erzeugt
```

```
##
## Parameter Estimates:
##
##   Information                                 Expected
##   Information saturated (h1) model          Structured
##   Standard Errors                             Standard
##
##
## Group 1 [within]:
##
## Latent Variables:
##                        Estimate  Std.Err  z-value  P(>|z|)   Std.lv
##   f1 =~
##     Atnmy_PE_1            1.000                               0.482
##     Atnmy_PE_2            1.545    0.157    9.842    0.000    0.745
##     Atnmy_PE_3            1.514    0.165    9.155    0.000    0.730
##     Atnmy_PE_4            1.815    0.174   10.446    0.000    0.876
##   f2 =~
##     Rltdn_PE_1            1.000                               0.589
##     Rltdn_PE_2            1.642    0.148   11.120    0.000    0.966
##     Rltdn_PE_3            1.466    0.130   11.258    0.000    0.863
##   f3 =~
##     Cmptn_PE_1            1.000                               0.811
##     Cmptn_PE_2            1.102    0.063   17.612    0.000    0.894
##     Cmptn_PE_3            0.829    0.058   14.299    0.000    0.672
##     Cmptn_PE_4            0.922    0.055   16.662    0.000    0.748
##   Autonomy_PE_1b =~
##     Atnmy_PE_1 (x)        1.000                               0.000
##   Autonomy_PE_2b =~
##     Atnmy_PE_2 (x)        1.000                               0.000
##   Autonomy_PE_3b =~
##     Atnmy_PE_3 (x)        1.000                               0.000
##   Autonomy_PE_4b =~
##     Atnmy_PE_4 (x)        1.000                               0.000
##   Relatedness_PE_1b =~
##     Rltdn_PE_1 (x)        1.000                               0.000
##   Relatedness_PE_2b =~
##     Rltdn_PE_2 (x)        1.000                               0.000
##   Relatedness_PE_3b =~
##     Rltdn_PE_3 (x)        1.000                               0.000
##   Competence_PE_1b =~
##     Cmptn_PE_1 (x)        1.000                               0.000
##   Competence_PE_2b =~
##     Cmptn_PE_2 (x)        1.000                               0.000
##   Competence_PE_3b =~
##     Cmptn_PE_3 (x)        1.000                               0.000
##   Competence_PE_4b =~
##     Cmptn_PE_4 (x)        1.000                               0.000
##   f1b =~
##     Atnmy_PE_1            0.000                                 NaN
##     Atnmy_PE_2            0.000                                 NaN
##     Atnmy_PE_3            0.000                                 NaN
##     Atnmy_PE_4            0.000                                 NaN
##   f2b =~
##     Rltdn_PE_1            0.000                                 NaN
##     Rltdn_PE_2            0.000                                 NaN
##     Rltdn_PE_3            0.000                                 NaN
##   f3b =~
##     Cmptn_PE_1            0.000                                 NaN
##     Cmptn_PE_2            0.000                                 NaN
```

```
##     Cmptn_PE_3            0.000                                NaN
##     Cmptn_PE_4            0.000                                NaN
##   Std.all
##
##     0.538
##     0.720
##     0.633
##     0.871
##
##     0.587
##     0.896
##     0.773
##
##     0.847
##     0.801
##     0.677
##     0.765
##
##     0.000
##
##     0.000
##
##     0.000
##
##     0.000
##
##     0.000
##
##     0.000
##
##     0.000
##
##     0.000
##
##     0.000
##
##     0.000
##
##     0.000
##
##       NaN
##       NaN
##       NaN
##       NaN
##
##       NaN
##       NaN
##       NaN
##
##       NaN
##       NaN
##       NaN
##       NaN
##
## Covariances:
##                    Estimate  Std.Err  z-value  P(>|z|)   Std.lv  Std.all
##   f1 ~~
##     f2                0.104    0.021    4.924    0.000    0.366    0.366
##     f3                0.233    0.033    7.057    0.000    0.595    0.595
##   f2 ~~
```

```
##     f3                0.199    0.034    5.923    0.000    0.416    0.416
##   f1 ~~
##     f1b               0.000                               NaN      NaN
##   f2 ~~
##     f1b               0.000                               NaN      NaN
##   f3 ~~
##     f1b               0.000                               NaN      NaN
##   f1b ~~
##     f2b               0.000                               NaN      NaN
##     f3b               0.000                               NaN      NaN
##   f1 ~~
##     f2b               0.000                               NaN      NaN
##   f2 ~~
##     f2b               0.000                               NaN      NaN
##   f3 ~~
##     f2b               0.000                               NaN      NaN
##   f2b ~~
##     f3b               0.000                               NaN      NaN
##   f1 ~~
##     f3b               0.000                               NaN      NaN
##   f2 ~~
##     f3b               0.000                               NaN      NaN
##   f3 ~~
##     f3b               0.000                               NaN      NaN
##
## Variances:
##                    Estimate  Std.Err  z-value  P(>|z|)   Std.lv  Std.all
##    .Atnmy_PE_1 (a)    0.570    0.044   13.090    0.000    0.570    0.710
##    .Atnmy_PE_2 (b)    0.517    0.047   11.079    0.000    0.517    0.482
##    .Atnmy_PE_3 (c)    0.797    0.065   12.352    0.000    0.797    0.599
##    .Atnmy_PE_4 (d)    0.244    0.040    6.091    0.000    0.244    0.242
##    .Rltdn_PE_1 (e)    0.658    0.052   12.736    0.000    0.658    0.655
##    .Rltdn_PE_2 (f)    0.229    0.057    4.049    0.000    0.229    0.197
##    .Rltdn_PE_3 (g)    0.500    0.056    8.879    0.000    0.500    0.402
##    .Cmptn_PE_1 (i)    0.259    0.029    8.850    0.000    0.259    0.283
##    .Cmptn_PE_2 (j)    0.446    0.043   10.376    0.000    0.446    0.358
##    .Cmptn_PE_3 (k)    0.534    0.043   12.416    0.000    0.534    0.541
##    .Cmptn_PE_4 (l)    0.397    0.035   11.213    0.000    0.397    0.415
##     f1                0.233    0.043    5.383    0.000    1.000    1.000
##     f2                0.346    0.058    5.955    0.000    1.000    1.000
##     f3                0.658    0.066    9.928    0.000    1.000    1.000
##     Atnmy_PE_1        0.000                               NaN      NaN
##     Atnmy_PE_2        0.000                               NaN      NaN
##     Atnmy_PE_3        0.000                               NaN      NaN
##     Atnmy_PE_4        0.000                               NaN      NaN
##     Rltdn_PE_1        0.000                               NaN      NaN
##     Rltdn_PE_2        0.000                               NaN      NaN
##     Rltdn_PE_3        0.000                               NaN      NaN
##     Cmptn_PE_1        0.000                               NaN      NaN
##     Cmptn_PE_2        0.000                               NaN      NaN
##     Cmptn_PE_3        0.000                               NaN      NaN
##     Cmptn_PE_4        0.000                               NaN      NaN
##     f1b               0.000                               NaN      NaN
##     f2b               0.000                               NaN      NaN
##     f3b               0.000                               NaN      NaN
##
##
## Group 2 [between]:
##
## Latent Variables:
```

```
##                          Estimate  Std.Err  z-value  P(>|z|)   Std.lv
##   f1 =~
##     Atn_PE_1                1.000                                  NaN
##     Atn_PE_2               -0.632    2.071   -0.305    0.760      NaN
##     Atn_PE_3               -1.746    3.830   -0.456    0.648      NaN
##     Atn_PE_4               -0.230    1.691   -0.136    0.892      NaN
##   f2 =~
##     Rlt_PE_1                1.000                                0.393
##     Rlt_PE_2                1.899    1.161    1.635    0.102    0.746
##     Rlt_PE_3                1.020    0.629    1.623    0.105    0.401
##   f3 =~
##     Cmp_PE_1                1.000                                0.832
##     Cmp_PE_2                1.119    0.266    4.208    0.000    0.931
##     Cmp_PE_3                1.032    0.485    2.128    0.033    0.859
##     Cmp_PE_4                0.791    0.249    3.179    0.001    0.659
##   Autonomy_PE_1b =~
##     Atn_PE_1 (sqc)          1.000                                1.777
##   Autonomy_PE_2b =~
##     Atn_PE_2 (sqc)          1.000                                1.178
##   Autonomy_PE_3b =~
##     Atn_PE_3 (sqc)          1.000                                1.320
##   Autonomy_PE_4b =~
##     Atn_PE_4 (sqc)          1.000                                1.489
##   Relatedness_PE_1b =~
##     Rlt_PE_1 (sqc)          1.000                                0.571
##   Relatedness_PE_2b =~
##     Rlt_PE_2 (sqc)          1.000                                0.203
##   Relatedness_PE_3b =~
##     Rlt_PE_3 (sqc)          1.000                                  NaN
##   Competence_PE_1b =~
##     Cmp_PE_1 (sqc)          1.000                                  NaN
##   Competence_PE_2b =~
##     Cmp_PE_2 (sqc)          1.000                                  NaN
##   Competence_PE_3b =~
##     Cmp_PE_3 (sqc)          1.000                                  NaN
##   Competence_PE_4b =~
##     Cmp_PE_4 (sqc)          1.000                                0.413
##   f1b =~
##     Atn_PE_1                1.000                                0.779
##     Atn_PE_2                0.908    0.207    4.396    0.000    1.068
##     Atn_PE_3                0.964    0.266    3.630    0.000    1.011
##     Atn_PE_4                1.042    0.222    4.684    0.000    0.969
##   f2b =~
##     Rlt_PE_1                1.000                                1.144
##     Rlt_PE_2                0.737    0.241    3.059    0.002    2.366
##     Rlt_PE_3                0.470    0.185    2.541    0.011      NaN
##   f3b =~
##     Cmp_PE_1                1.000                                  NaN
##     Cmp_PE_2                1.340    0.446    3.003    0.003      NaN
##     Cmp_PE_3                3.239    2.375    1.364    0.173      NaN
##     Cmp_PE_4                0.376    0.549    0.685    0.493      NaN
##   Std.all
##
##       NaN
##       NaN
##       NaN
##       NaN
##
##     0.368
##     0.820
```

```
##     0.533
##
##     0.866
##     0.868
##     0.786
##     0.658
##
##     0.929
##
##     0.860
##
##     0.866
##
##     0.950
##
##     0.535
##
##     0.224
##
##       NaN
##
##       NaN
##
##       NaN
##
##       NaN
##
##     0.413
##
##     0.779
##     1.068
##     1.011
##     0.969
##
##     1.144
##     2.366
##       NaN
##
##       NaN
##       NaN
##       NaN
##       NaN
##
## Covariances:
##                    Estimate  Std.Err  z-value  P(>|z|)   Std.lv  Std.all
##   f1 ~~
##     f2               -0.103    0.181   -0.571    0.568   -0.994   -0.994
##     f3               -0.148    0.241   -0.613    0.540   -0.669   -0.669
##   f2 ~~
##     f3                0.015    0.111    0.139    0.889    0.047    0.047
##   f1 ~~
##     f1b               0.000                               0.000    0.000
##   f2 ~~
##     f1b               0.000                               0.000    0.000
##   f3 ~~
##     f1b               0.000                               0.000    0.000
##   f1b ~~
##     f2b               0.207    0.339    0.612    0.541    0.229    0.229
##     f3b               0.222    0.229    0.970    0.332    0.570    0.570
##   f1 ~~
```

```
##     f2b               0.000                               0.000    0.000
##   f2 ~~
##     f2b               0.000                               0.000    0.000
##   f3 ~~
##     f2b               0.000                               0.000    0.000
##   f2b ~~
##     f3b               0.207    0.192    1.078    0.281    1.128    1.128
##   f1 ~~
##     f3b               0.000                               0.000    0.000
##   f2 ~~
##     f3b               0.000                               0.000    0.000
##   f3 ~~
##     f3b               0.000                               0.000    0.000
##
## Variances:
##                    Estimate  Std.Err  z-value  P(>|z|)   Std.lv  Std.all
##    .Atnmy_PE_1 (a)    0.570    0.044   13.090    0.000    0.570    0.156
##    .Atnmy_PE_2 (b)    0.517    0.047   11.079    0.000    0.517    0.275
##    .Atnmy_PE_3 (c)    0.797    0.065   12.352    0.000    0.797    0.343
##    .Atnmy_PE_4 (d)    0.244    0.040    6.091    0.000    0.244    0.099
##    .Rltdn_PE_1 (e)    0.658    0.052   12.736    0.000    0.658    0.578
##    .Rltdn_PE_2 (f)    0.229    0.057    4.049    0.000    0.229    0.277
##    .Rltdn_PE_3 (g)    0.500    0.056    8.879    0.000    0.500    0.884
##    .Cmptn_PE_1 (i)    0.259    0.029    8.850    0.000    0.259    0.281
##    .Cmptn_PE_2 (j)    0.446    0.043   10.376    0.000    0.446    0.388
##    .Cmptn_PE_3 (k)    0.534    0.043   12.416    0.000    0.534    0.447
##    .Cmptn_PE_4 (l)    0.397    0.035   11.213    0.000    0.397    0.396
##     f1               -0.070    0.212   -0.332    0.740      NaN      NaN
##     f2                0.154    0.245    0.630    0.529    1.000    1.000
##     f3                0.693    0.327    2.121    0.034    1.000    1.000
##     Atnmy_PE_1        1.241    0.594    2.089    0.037    0.393    0.393
##     Atnmy_PE_2       -0.194    0.140   -1.383    0.167   -0.140   -0.140
##     Atnmy_PE_3       -0.040    0.359   -0.111    0.911   -0.023   -0.023
##     Atnmy_PE_4        0.136    0.173    0.786    0.432    0.061    0.061
##     Rltdn_PE_1       -0.100    0.212   -0.472    0.637   -0.308   -0.308
##     Rltdn_PE_2       -0.190    0.159   -1.198    0.231   -4.599   -4.599
##     Rltdn_PE_3       -0.189    0.106   -1.789    0.074      NaN      NaN
##     Cmptn_PE_1        0.051    0.138    0.373    0.709      NaN      NaN
##     Cmptn_PE_2       -0.021    0.177   -0.121    0.904      NaN      NaN
##     Cmptn_PE_3        0.753    0.765    0.984    0.325      NaN      NaN
##     Cmptn_PE_4        0.182    0.181    1.008    0.313    1.066    1.066
##     f1b               1.918    0.873    2.196    0.028    1.000    1.000
##     f2b               0.426    0.397    1.074    0.283    1.000    1.000
##     f3b              -0.079    0.181   -0.438    0.661      NaN      NaN
```

Path diagramm

```
semPlot::semPaths(models$mod_3x3_free_vareq_fit,"std",edge.label.cex=0.5, curvePivot = TRUE)
```

```
## Warning in sqrt(ETA2): NaNs wurden erzeugt

## Warning in sqrt(ETA2): NaNs wurden erzeugt

## Warning in sqrt(ETA2): NaNs wurden erzeugt
```

```
## Warning in qgraph::qgraph(Edgelist, labels = nLab, bidirectional = Bidir, :
## Non-finite weights are omitted

## Warning in qgraph::qgraph(Edgelist, labels = nLab, bidirectional = Bidir, :
## Non-finite weights are omitted
```

1

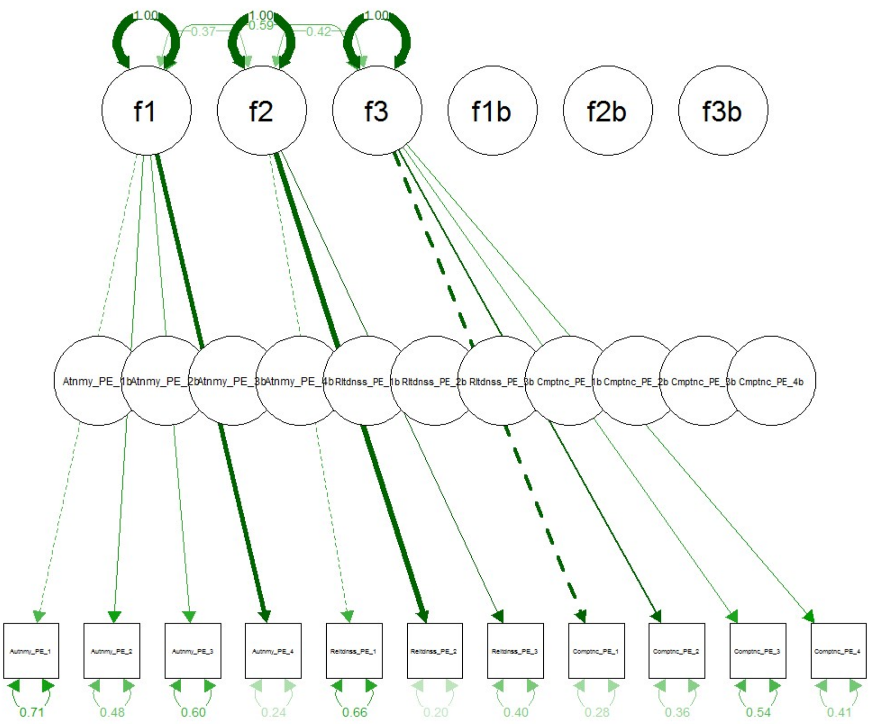

2

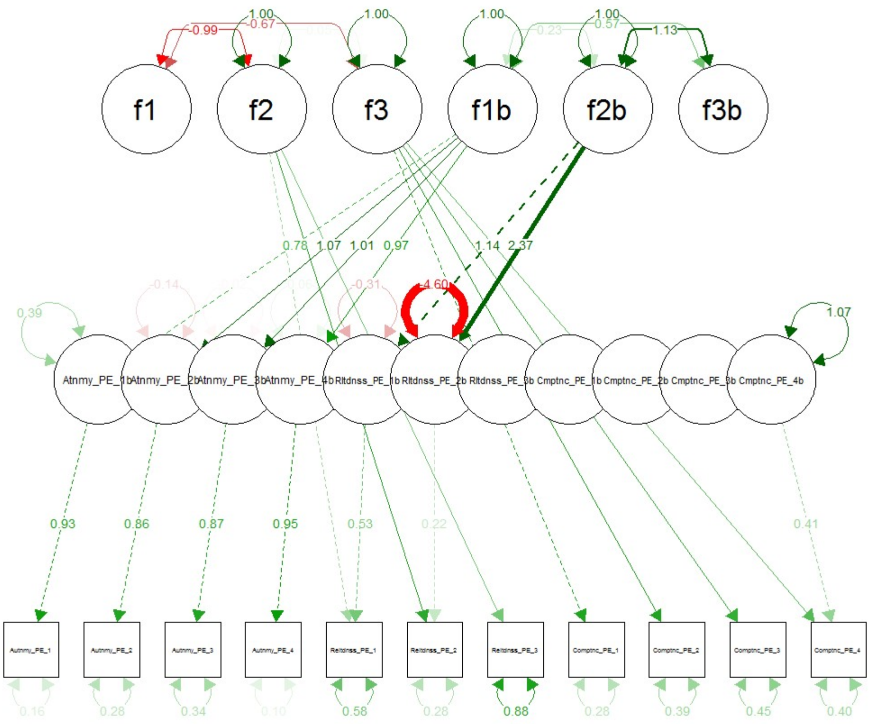

3x3, freely estimated loadings and latent factor variances and

covariances, manifest variances set equal/fixed where necessary

```
mod_3x3_free_vareq_var <- '
f1 =~ Autonomy_PE_1 + Autonomy_PE_2 + Autonomy_PE_3 + Autonomy_PE_4
f2 =~ Relatedness_PE_1 + Relatedness_PE_2 + Relatedness_PE_3
f3 =~ Competence_PE_1 + Competence_PE_2 + Competence_PE_3 + Competence_PE_4
Autonomy_PE_1 ~~ c(a,a)*Autonomy_PE_1
Autonomy_PE_2 ~~ c(NA,0)*Autonomy_PE_2
Autonomy_PE_3 ~~ c(NA,0)*Autonomy_PE_3
Autonomy_PE_4 ~~ c(d,d)*Autonomy_PE_4
Relatedness_PE_1 ~~ c(NA,0)*Relatedness_PE_1
Relatedness_PE_2 ~~ c(NA,0)*Relatedness_PE_2
Relatedness_PE_3 ~~ c(NA,0)*Relatedness_PE_3
Competence_PE_1 ~~ c(e,e)*Competence_PE_1
Competence_PE_2 ~~ c(NA,0)*Competence_PE_2
Competence_PE_3 ~~ c(m,m)*Competence_PE_3
Competence_PE_4 ~~ c(NA,0)*Competence_PE_4
f1 ~~ f1
f2 ~~ f2
f3 ~~ f3
f1 ~~ f2
f1 ~~ f3
f2 ~~ f3
Autonomy_PE_1b =~ c(0,x$sqc)*Autonomy_PE_1
Autonomy_PE_1b ~~ c(0,NA)*Autonomy_PE_1b
Autonomy_PE_2b =~ c(0,x$sqc)*Autonomy_PE_2
Autonomy_PE_2b ~~ c(0,NA)*Autonomy_PE_2b
Autonomy_PE_3b =~ c(0,x$sqc)*Autonomy_PE_3
Autonomy_PE_3b ~~ c(0,NA)*Autonomy_PE_3b
Autonomy_PE_4b =~ c(0,x$sqc)*Autonomy_PE_4
Autonomy_PE_4b ~~ c(0,NA)*Autonomy_PE_4b
Relatedness_PE_1b =~ c(0,x$sqc)*Relatedness_PE_1
Relatedness_PE_1b ~~ c(0,NA)*Relatedness_PE_1b
Relatedness_PE_2b =~ c(0,x$sqc)*Relatedness_PE_2
Relatedness_PE_2b ~~ c(0,NA)*Relatedness_PE_2b
Relatedness_PE_3b =~ c(0,x$sqc)*Relatedness_PE_3
Relatedness_PE_3b ~~ c(0,NA)*Relatedness_PE_3b
Competence_PE_1b =~ c(0,x$sqc)*Competence_PE_1
Competence_PE_1b ~~ c(0,NA)*Competence_PE_1b
Competence_PE_2b =~ c(0,x$sqc)*Competence_PE_2
Competence_PE_2b ~~ c(0,NA)*Competence_PE_2b
Competence_PE_3b =~ c(0,x$sqc)*Competence_PE_3
Competence_PE_3b ~~ c(0,NA)*Competence_PE_3b
Competence_PE_4b =~ c(0,x$sqc)*Competence_PE_4
Competence_PE_4b ~~ c(0,NA)*Competence_PE_4b
f1b =~ c(0,1)*Autonomy_PE_1b + c(0,NA)*Autonomy_PE_2b + c(0,NA)*Autonomy_PE_3b + c(0,NA)*Aut
onomy_PE_4b
f2b =~ c(0,1)*Relatedness_PE_1b + c(0,NA)*Relatedness_PE_2b + c(0,NA)*Relatedness_PE_3b
f3b =~ c(0,1)*Competence_PE_1b + c(0,NA)*Competence_PE_2b + c(0,NA)*Competence_PE_3b + c(0,N
A)*Competence_PE_4b
f1b ~~ c(0,NA)*f1b + c(0,0)*f1 + c(0,0)*f2 + c(0,0)*f3 + c(0,NA)*f2b + c(0,NA)*f3b
f2b ~~ c(0,NA)*f2b + c(0,0)*f1 + c(0,0)*f2 + c(0,0)*f3 + c(0,NA)*f3b
f3b ~~ c(0,NA)*f3b + c(0,0)*f1 + c(0,0)*f2 + c(0,0)*f3
'
# fit measures
models$mod_3x3_free_vareq_var_fit <- cfa(mod_3x3_free_vareq_var, sample.cov = combined.cov,
sample.nobs = combined.n, orthogonal = T)
```

```
## Warning in lav_object_post_check(object): lavaan WARNING: some estimated lv
## variances are negative
```

```
summary(models$mod_3x3_free_vareq_var_fit, fit.measures = T, standardized = T)
```

```
## lavaan 0.6-3 ended normally after 483 iterations
##
##   Optimization method                           NLMINB
##   Number of free parameters                         68
##   Number of equality constraints                     4
##
##   Number of observations per group
##   within                                           400
##   between                                           33
##
##   Estimator                                         ML
##   Model Fit Test Statistic                     146.746
##   Degrees of freedom                                68
##   P-value (Chi-square)                           0.000
##
## Chi-square for each group:
##
##   within                                       124.159
##   between                                       22.587
##
## Model test baseline model:
##
##   Minimum Function Test Statistic             2130.224
##   Degrees of freedom                               110
##   P-value                                        0.000
##
## User model versus baseline model:
##
##   Comparative Fit Index (CFI)                    0.961
##   Tucker-Lewis Index (TLI)                       0.937
##
## Loglikelihood and Information Criteria:
##
##   Loglikelihood user model (H0)              -5940.740
##   Loglikelihood unrestricted model (H1)      -5867.367
##
##   Number of free parameters                         64
##   Akaike (AIC)                               12009.480
##   Bayesian (BIC)                             12270.007
##   Sample-size adjusted Bayesian (BIC)        12066.907
##
## Root Mean Square Error of Approximation:
##
##   RMSEA                                          0.073
##   90 Percent Confidence Interval          0.057  0.089
##   P-value RMSEA <= 0.05                          0.011
##
## Standardized Root Mean Square Residual:
##
##   SRMR                                           0.056
```

```
## Warning in sqrt(ETA2): NaNs wurden erzeugt
```

```
## Warning in sqrt(ETA2): NaNs wurden erzeugt

## Warning in sqrt(ETA2): NaNs wurden erzeugt
```

```
##
## Parameter Estimates:
##
##   Information                                 Expected
##   Information saturated (h1) model          Structured
##   Standard Errors                             Standard
##
##
## Group 1 [within]:
##
## Latent Variables:
##                        Estimate  Std.Err  z-value  P(>|z|)   Std.lv
##   f1 =~
##     Atnmy_PE_1            1.000                                0.482
##     Atnmy_PE_2            1.545    0.157    9.842    0.000    0.745
##     Atnmy_PE_3            1.514    0.165    9.155    0.000    0.730
##     Atnmy_PE_4            1.815    0.174   10.446    0.000    0.876
##   f2 =~
##     Rltdn_PE_1            1.000                                0.589
##     Rltdn_PE_2            1.642    0.148   11.120    0.000    0.966
##     Rltdn_PE_3            1.466    0.130   11.258    0.000    0.863
##   f3 =~
##     Cmptn_PE_1            1.000                                0.811
##     Cmptn_PE_2            1.102    0.063   17.612    0.000    0.894
##     Cmptn_PE_3            0.829    0.058   14.299    0.000    0.672
##     Cmptn_PE_4            0.922    0.055   16.662    0.000    0.748
##   Autonomy_PE_1b =~
##     Atnmy_PE_1 (x)        1.000                                0.000
##   Autonomy_PE_2b =~
##     Atnmy_PE_2 (x)        1.000                                0.000
##   Autonomy_PE_3b =~
##     Atnmy_PE_3 (x)        1.000                                0.000
##   Autonomy_PE_4b =~
##     Atnmy_PE_4 (x)        1.000                                0.000
##   Relatedness_PE_1b =~
##     Rltdn_PE_1 (x)        1.000                                0.000
##   Relatedness_PE_2b =~
##     Rltdn_PE_2 (x)        1.000                                0.000
##   Relatedness_PE_3b =~
##     Rltdn_PE_3 (x)        1.000                                0.000
##   Competence_PE_1b =~
##     Cmptn_PE_1 (x)        1.000                                0.000
##   Competence_PE_2b =~
##     Cmptn_PE_2 (x)        1.000                                0.000
##   Competence_PE_3b =~
##     Cmptn_PE_3 (x)        1.000                                0.000
##   Competence_PE_4b =~
##     Cmptn_PE_4 (x)        1.000                                0.000
##   f1b =~
##     Atnmy_PE_1            0.000                                  NaN
##     Atnmy_PE_2            0.000                                  NaN
##     Atnmy_PE_3            0.000                                  NaN
##     Atnmy_PE_4            0.000                                  NaN
##   f2b =~
##     Rltdn_PE_1            0.000                                  NaN
##     Rltdn_PE_2            0.000                                  NaN
##     Rltdn_PE_3            0.000                                  NaN
##   f3b =~
##     Cmptn_PE_1            0.000                                  NaN
##     Cmptn_PE_2            0.000                                  NaN
```

```
##     Cmptn_PE_3            0.000                                      NaN
##     Cmptn_PE_4            0.000                                      NaN
##   Std.all
##
##     0.538
##     0.720
##     0.633
##     0.871
##
##     0.587
##     0.896
##     0.773
##
##     0.847
##     0.801
##     0.677
##     0.765
##
##     0.000
##
##     0.000
##
##     0.000
##
##     0.000
##
##     0.000
##
##     0.000
##
##     0.000
##
##     0.000
##
##     0.000
##
##     0.000
##
##     0.000
##
##       NaN
##       NaN
##       NaN
##       NaN
##
##       NaN
##       NaN
##       NaN
##
##       NaN
##       NaN
##       NaN
##       NaN
##
## Covariances:
##                    Estimate  Std.Err  z-value  P(>|z|)   Std.lv  Std.all
##   f1 ~~
##     f2                0.104    0.021    4.924    0.000    0.366    0.366
##     f3                0.233    0.033    7.057    0.000    0.595    0.595
##   f2 ~~
```

```
##     f3               0.199    0.034    5.923    0.000    0.416    0.416
##   f1 ~~
##     f1b              0.000                               NaN      NaN
##   f2 ~~
##     f1b              0.000                               NaN      NaN
##   f3 ~~
##     f1b              0.000                               NaN      NaN
##   f1b ~~
##     f2b              0.000                               NaN      NaN
##     f3b              0.000                               NaN      NaN
##   f1 ~~
##     f2b              0.000                               NaN      NaN
##   f2 ~~
##     f2b              0.000                               NaN      NaN
##   f3 ~~
##     f2b              0.000                               NaN      NaN
##   f2b ~~
##     f3b              0.000                               NaN      NaN
##   f1 ~~
##     f3b              0.000                               NaN      NaN
##   f2 ~~
##     f3b              0.000                               NaN      NaN
##   f3 ~~
##     f3b              0.000                               NaN      NaN
##
## Variances:
##                    Estimate  Std.Err  z-value  P(>|z|)   Std.lv  Std.all
##    .Atnmy_PE_1 (a)    0.570    0.044   13.090    0.000    0.570    0.710
##    .Atnmy_PE_2        0.517    0.047   11.079    0.000    0.517    0.482
##    .Atnmy_PE_3        0.797    0.065   12.352    0.000    0.797    0.599
##    .Atnmy_PE_4 (d)    0.244    0.040    6.091    0.000    0.244    0.242
##    .Rltdn_PE_1        0.658    0.052   12.736    0.000    0.658    0.655
##    .Rltdn_PE_2        0.229    0.057    4.049    0.000    0.229    0.197
##    .Rltdn_PE_3        0.500    0.056    8.879    0.000    0.500    0.402
##    .Cmptn_PE_1 (e)    0.259    0.029    8.850    0.000    0.259    0.283
##    .Cmptn_PE_2        0.446    0.043   10.376    0.000    0.446    0.358
##    .Cmptn_PE_3 (m)    0.534    0.043   12.416    0.000    0.534    0.541
##    .Cmptn_PE_4        0.397    0.035   11.213    0.000    0.397    0.415
##     f1               0.233    0.043    5.383    0.000    1.000    1.000
##     f2               0.346    0.058    5.955    0.000    1.000    1.000
##     f3               0.658    0.066    9.928    0.000    1.000    1.000
##     Atnmy_PE_1       0.000                               NaN      NaN
##     Atnmy_PE_2       0.000                               NaN      NaN
##     Atnmy_PE_3       0.000                               NaN      NaN
##     Atnmy_PE_4       0.000                               NaN      NaN
##     Rltdn_PE_1       0.000                               NaN      NaN
##     Rltdn_PE_2       0.000                               NaN      NaN
##     Rltdn_PE_3       0.000                               NaN      NaN
##     Cmptn_PE_1       0.000                               NaN      NaN
##     Cmptn_PE_2       0.000                               NaN      NaN
##     Cmptn_PE_3       0.000                               NaN      NaN
##     Cmptn_PE_4       0.000                               NaN      NaN
##     f1b              0.000                               NaN      NaN
##     f2b              0.000                               NaN      NaN
##     f3b              0.000                               NaN      NaN
##
##
## Group 2 [between]:
##
## Latent Variables:
```

```
##                          Estimate  Std.Err  z-value  P(>|z|)   Std.lv
##    f1 =~
##      Atn_PE_1               1.000                                 NaN
##      Atn_PE_2              -0.632    2.071   -0.305    0.760      NaN
##      Atn_PE_3              -1.746    3.830   -0.456    0.648      NaN
##      Atn_PE_4              -0.230    1.691   -0.136    0.892      NaN
##    f2 =~
##      Rlt_PE_1               1.000                               0.393
##      Rlt_PE_2               1.899    1.161    1.635    0.102    0.746
##      Rlt_PE_3               1.020    0.629    1.623    0.105    0.401
##    f3 =~
##      Cmp_PE_1               1.000                               0.832
##      Cmp_PE_2               1.119    0.266    4.208    0.000    0.931
##      Cmp_PE_3               1.032    0.485    2.128    0.033    0.859
##      Cmp_PE_4               0.791    0.249    3.179    0.001    0.659
##    Autonomy_PE_1b =~
##      Atn_PE_1 (sqc)         1.000                               1.777
##    Autonomy_PE_2b =~
##      Atn_PE_2 (sqc)         1.000                               1.380
##    Autonomy_PE_3b =~
##      Atn_PE_3 (sqc)         1.000                               1.593
##    Autonomy_PE_4b =~
##      Atn_PE_4 (sqc)         1.000                               1.489
##    Relatedness_PE_1b =~
##      Rlt_PE_1 (sqc)         1.000                               0.992
##    Relatedness_PE_2b =~
##      Rlt_PE_2 (sqc)         1.000                               0.520
##    Relatedness_PE_3b =~
##      Rlt_PE_3 (sqc)         1.000                               0.636
##    Competence_PE_1b =~
##      Cmp_PE_1 (sqc)         1.000                                 NaN
##    Competence_PE_2b =~
##      Cmp_PE_2 (sqc)         1.000                               0.532
##    Competence_PE_3b =~
##      Cmp_PE_3 (sqc)         1.000                                 NaN
##    Competence_PE_4b =~
##      Cmp_PE_4 (sqc)         1.000                               0.753
##    f1b =~
##      Atn_PE_1               1.000                               0.779
##      Atn_PE_2               0.908    0.207    4.396    0.000    0.911
##      Atn_PE_3               0.964    0.266    3.630    0.000    0.838
##      Atn_PE_4               1.042    0.222    4.684    0.000    0.969
##    f2b =~
##      Rlt_PE_1               1.000                               0.658
##      Rlt_PE_2               0.737    0.241    3.059    0.002    0.925
##      Rlt_PE_3               0.470    0.185    2.541    0.011    0.481
##    f3b =~
##      Cmp_PE_1               1.000                                 NaN
##      Cmp_PE_2               1.340    0.446    3.004    0.003      NaN
##      Cmp_PE_3               3.239    2.375    1.364    0.173      NaN
##      Cmp_PE_4               0.376    0.549    0.685    0.493      NaN
##    Std.all
##
##        NaN
##        NaN
##        NaN
##        NaN
##
##      0.368
##      0.820
```

```
##     0.533
##
##     0.866
##     0.868
##     0.786
##     0.658
##
##     0.929
##
##     1.007
##
##     1.045
##
##     0.950
##
##     0.930
##
##     0.572
##
##     0.846
##
##       NaN
##
##     0.496
##
##       NaN
##
##     0.753
##
##     0.779
##     0.911
##     0.838
##     0.969
##
##     0.658
##     0.925
##     0.481
##
##       NaN
##       NaN
##       NaN
##       NaN
##
## Covariances:
##                    Estimate  Std.Err  z-value  P(>|z|)   Std.lv  Std.all
##   f1 ~~
##     f2               -0.103    0.181   -0.571    0.568   -0.994   -0.994
##     f3               -0.148    0.241   -0.613    0.540   -0.669   -0.669
##   f2 ~~
##     f3                0.015    0.111    0.139    0.890    0.047    0.047
##   f1 ~~
##     f1b               0.000                                0.000    0.000
##   f2 ~~
##     f1b               0.000                                0.000    0.000
##   f3 ~~
##     f1b               0.000                                0.000    0.000
##   f1b ~~
##     f2b               0.207    0.339    0.612    0.541    0.229    0.229
##     f3b               0.222    0.229    0.970    0.332    0.570    0.570
##   f1 ~~
```

```
##     f2b              0.000                               0.000    0.000
##   f2 ~~
##     f2b              0.000                               0.000    0.000
##   f3 ~~
##     f2b              0.000                               0.000    0.000
##   f2b ~~
##     f3b              0.207    0.192    1.078    0.281    1.128    1.128
##   f1 ~~
##     f3b              0.000                               0.000    0.000
##   f2 ~~
##     f3b              0.000                               0.000    0.000
##   f3 ~~
##     f3b              0.000                               0.000    0.000
##
## Variances:
##                    Estimate  Std.Err  z-value  P(>|z|)   Std.lv  Std.all
##    .Atnmy_PE_1 (a)    0.570    0.044   13.090    0.000    0.570    0.156
##    .Atnmy_PE_2        0.000                               0.000    0.000
##    .Atnmy_PE_3        0.000                               0.000    0.000
##    .Atnmy_PE_4 (d)    0.244    0.040    6.091    0.000    0.244    0.099
##    .Rltdn_PE_1        0.000                               0.000    0.000
##    .Rltdn_PE_2        0.000                               0.000    0.000
##    .Rltdn_PE_3        0.000                               0.000    0.000
##    .Cmptn_PE_1 (e)    0.259    0.029    8.850    0.000    0.259    0.281
##    .Cmptn_PE_2        0.000                               0.000    0.000
##    .Cmptn_PE_3 (m)    0.534    0.043   12.416    0.000    0.534    0.447
##    .Cmptn_PE_4        0.000                               0.000    0.000
##     f1               -0.070    0.212   -0.332    0.740      NaN      NaN
##     f2                0.154    0.245    0.630    0.529    1.000    1.000
##     f3                0.693    0.327    2.121    0.034    1.000    1.000
##     Atnmy_PE_1        1.241    0.594    2.089    0.037    0.393    0.393
##     Atnmy_PE_2        0.322    0.132    2.434    0.015    0.169    0.169
##     Atnmy_PE_3        0.757    0.354    2.141    0.032    0.298    0.298
##     Atnmy_PE_4        0.136    0.173    0.786    0.432    0.061    0.061
##     Rltdn_PE_1        0.558    0.206    2.708    0.007    0.567    0.567
##     Rltdn_PE_2        0.039    0.148    0.265    0.791    0.145    0.145
##     Rltdn_PE_3        0.311    0.089    3.484    0.000    0.768    0.768
##     Cmptn_PE_1        0.051    0.138    0.373    0.709      NaN      NaN
##     Cmptn_PE_2        0.425    0.172    2.472    0.013    1.503    1.503
##     Cmptn_PE_3        0.753    0.765    0.984    0.325      NaN      NaN
##     Cmptn_PE_4        0.579    0.177    3.270    0.001    1.020    1.020
##     f1b               1.918    0.873    2.196    0.028    1.000    1.000
##     f2b               0.426    0.397    1.074    0.283    1.000    1.000
##     f3b              -0.079    0.181   -0.438    0.661      NaN      NaN
```

3x3, not nested, separated

Idea of a separate measurement and structural model on both levels for the 3x3 models

```
mod_3x3_sep <- '
f1 =~ c(1,0)*Autonomy_PE_1 + c(NA,0)*Autonomy_PE_2 + c(NA,0)*Autonomy_PE_3 + c(NA,0)*Autonom
y_PE_4
f2 =~ c(1,0)*Relatedness_PE_1 + c(NA,0)*Relatedness_PE_2 + c(NA,0)*Relatedness_PE_3
f3 =~ c(1,0)*Competence_PE_1 + c(NA,0)*Competence_PE_2 + c(NA,0)*Competence_PE_3 + c(NA,0)*C
ompetence_PE_4
Autonomy_PE_1 ~~ c(j,j)*Autonomy_PE_1
Autonomy_PE_2 ~~ c(k,k)*Autonomy_PE_2
Autonomy_PE_3 ~~ c(l,l)*Autonomy_PE_3
Autonomy_PE_4 ~~ c(m,m)*Autonomy_PE_4
Relatedness_PE_1 ~~ c(n,n)*Relatedness_PE_1
Relatedness_PE_2 ~~ c(o,o)*Relatedness_PE_2
Relatedness_PE_3 ~~ c(p,p)*Relatedness_PE_3
Competence_PE_1 ~~ c(r,r)*Competence_PE_1
Competence_PE_2 ~~ c(s,s)*Competence_PE_2
Competence_PE_3 ~~ c(t,t)*Competence_PE_3
Competence_PE_4 ~~ c(u,u)*Competence_PE_4
f1 ~~ c(NA,0)*f1
f2 ~~ c(NA,0)*f2
f3 ~~ c(NA,0)*f3
f1 ~~ c(NA,0)*f2
f1 ~~ c(NA,0)*f3
f2 ~~ c(NA,0)*f3
Autonomy_PE_1b =~ c(0,x$sqc)*Autonomy_PE_1
Autonomy_PE_1b ~~ c(0,NA)*Autonomy_PE_1b
Autonomy_PE_2b =~ c(0,x$sqc)*Autonomy_PE_2
Autonomy_PE_2b ~~ c(0,NA)*Autonomy_PE_2b
Autonomy_PE_3b =~ c(0,x$sqc)*Autonomy_PE_3
Autonomy_PE_3b ~~ c(0,NA)*Autonomy_PE_3b
Autonomy_PE_4b =~ c(0,x$sqc)*Autonomy_PE_4
Autonomy_PE_4b ~~ c(0,NA)*Autonomy_PE_4b
Relatedness_PE_1b =~ c(0,x$sqc)*Relatedness_PE_1
Relatedness_PE_1b ~~ c(0,NA)*Relatedness_PE_1b
Relatedness_PE_2b =~ c(0,x$sqc)*Relatedness_PE_2
Relatedness_PE_2b ~~ c(0,NA)*Relatedness_PE_2b
Relatedness_PE_3b =~ c(0,x$sqc)*Relatedness_PE_3
Relatedness_PE_3b ~~ c(0,NA)*Relatedness_PE_3b
Competence_PE_1b =~ c(0,x$sqc)*Competence_PE_1
Competence_PE_1b ~~ c(0,NA)*Competence_PE_1b
Competence_PE_2b =~ c(0,x$sqc)*Competence_PE_2
Competence_PE_2b ~~ c(0,NA)*Competence_PE_2b
Competence_PE_3b =~ c(0,x$sqc)*Competence_PE_3
Competence_PE_3b ~~ c(0,NA)*Competence_PE_3b
Competence_PE_4b =~ c(0,x$sqc)*Competence_PE_4
Competence_PE_4b ~~ c(0,NA)*Competence_PE_4b
f1b =~ c(0,1)*Autonomy_PE_1b + c(0,NA)*Autonomy_PE_2b + c(0,NA)*Autonomy_PE_3b + c(0,NA)*Aut
onomy_PE_4b
f2b =~ c(0,1)*Relatedness_PE_1b + c(0,NA)*Relatedness_PE_2b + c(0,NA)*Relatedness_PE_3b
f3b =~ c(0,1)*Competence_PE_1b + c(0,NA)*Competence_PE_2b + c(0,NA)*Competence_PE_3b + c(0,N
A)*Competence_PE_4b
f1b ~~ c(0,NA)*f1b + c(0,0)*f1 + c(0,0)*f2 + c(0,0)*f3 + c(0,NA)*f2b + c(0,NA)*f3b
f2b ~~ c(0,NA)*f2b + c(0,0)*f1 + c(0,0)*f2 + c(0,0)*f3 + c(0,NA)*f3b
f3b ~~ c(0,NA)*f3b + c(0,0)*f1 + c(0,0)*f2 + c(0,0)*f3
'
# Fit hypothesized model
models$mod_3x3_sep_fit <- cfa(mod_3x3_sep, sample.cov = combined.cov, sample.nobs = combine
d.n, orthogonal = T)
```

```
## Warning in lav_object_post_check(object): lavaan WARNING: some estimated lv
## variances are negative
```

```
summary(models$mod_3x3_sep_fit, fit.measures = T, standardized = T)
```

```
## lavaan 0.6-3 ended normally after 91 iterations
##
##   Optimization method                           NLMINB
##   Number of free parameters                         61
##   Number of equality constraints                    11
##
##   Number of observations per group
##   within                                           400
##   between                                           33
##
##   Estimator                                         ML
##   Model Fit Test Statistic                     186.377
##   Degrees of freedom                                82
##   P-value (Chi-square)                           0.000
##
## Chi-square for each group:
##
##   within                                       124.159
##   between                                       62.218
##
## Model test baseline model:
##
##   Minimum Function Test Statistic             2130.224
##   Degrees of freedom                               110
##   P-value                                        0.000
##
## User model versus baseline model:
##
##   Comparative Fit Index (CFI)                    0.948
##   Tucker-Lewis Index (TLI)                       0.931
##
## Loglikelihood and Information Criteria:
##
##   Loglikelihood user model (H0)              -5960.555
##   Loglikelihood unrestricted model (H1)      -5867.367
##
##   Number of free parameters                         50
##   Akaike (AIC)                               12021.111
##   Bayesian (BIC)                             12224.647
##   Sample-size adjusted Bayesian (BIC)        12065.975
##
## Root Mean Square Error of Approximation:
##
##   RMSEA                                          0.077
##   90 Percent Confidence Interval          0.062  0.091
##   P-value RMSEA <= 0.05                          0.002
##
## Standardized Root Mean Square Residual:
##
##   SRMR                                           0.058
##
## Parameter Estimates:
##
##   Information                                 Expected
##   Information saturated (h1) model          Structured
##   Standard Errors                             Standard
##
##
## Group 1 [within]:
##
```

```
## Latent Variables:
##                        Estimate  Std.Err  z-value  P(>|z|)   Std.lv
##   f1 =~
##     Atnmy_PE_1            1.000                               0.482
##     Atnmy_PE_2            1.545    0.157    9.842    0.000    0.745
##     Atnmy_PE_3            1.514    0.165    9.155    0.000    0.730
##     Atnmy_PE_4            1.815    0.174   10.446    0.000    0.876
##   f2 =~
##     Rltdn_PE_1            1.000                               0.589
##     Rltdn_PE_2            1.642    0.148   11.120    0.000    0.966
##     Rltdn_PE_3            1.466    0.130   11.258    0.000    0.863
##   f3 =~
##     Cmptn_PE_1            1.000                               0.811
##     Cmptn_PE_2            1.102    0.063   17.612    0.000    0.894
##     Cmptn_PE_3            0.829    0.058   14.299    0.000    0.672
##     Cmptn_PE_4            0.922    0.055   16.662    0.000    0.748
##   Autonomy_PE_1b =~
##     Atnmy_PE_1 (x)        1.000                               0.000
##   Autonomy_PE_2b =~
##     Atnmy_PE_2 (x)        1.000                               0.000
##   Autonomy_PE_3b =~
##     Atnmy_PE_3 (x)        1.000                               0.000
##   Autonomy_PE_4b =~
##     Atnmy_PE_4 (x)        1.000                               0.000
##   Relatedness_PE_1b =~
##     Rltdn_PE_1 (x)        1.000                               0.000
##   Relatedness_PE_2b =~
##     Rltdn_PE_2 (x)        1.000                               0.000
##   Relatedness_PE_3b =~
##     Rltdn_PE_3 (x)        1.000                               0.000
##   Competence_PE_1b =~
##     Cmptn_PE_1 (x)        1.000                               0.000
##   Competence_PE_2b =~
##     Cmptn_PE_2 (x)        1.000                               0.000
##   Competence_PE_3b =~
##     Cmptn_PE_3 (x)        1.000                               0.000
##   Competence_PE_4b =~
##     Cmptn_PE_4 (x)        1.000                               0.000
##   f1b =~
##     Atnmy_PE_1            0.000                                 NaN
##     Atnmy_PE_2            0.000                                 NaN
##     Atnmy_PE_3            0.000                                 NaN
##     Atnmy_PE_4            0.000                                 NaN
##   f2b =~
##     Rltdn_PE_1            0.000                                 NaN
##     Rltdn_PE_2            0.000                                 NaN
##     Rltdn_PE_3            0.000                                 NaN
##   f3b =~
##     Cmptn_PE_1            0.000                                 NaN
##     Cmptn_PE_2            0.000                                 NaN
##     Cmptn_PE_3            0.000                                 NaN
##     Cmptn_PE_4            0.000                                 NaN
##   Std.all
##
##     0.538
##     0.720
##     0.633
##     0.871
##
##     0.587
```

```
##     0.896
##     0.773
##
##     0.847
##     0.801
##     0.677
##     0.765
##
##     0.000
##
##     0.000
##
##     0.000
##
##     0.000
##
##     0.000
##
##     0.000
##
##     0.000
##
##     0.000
##
##     0.000
##
##     0.000
##
##     0.000
##
##       NaN
##       NaN
##       NaN
##       NaN
##
##       NaN
##       NaN
##       NaN
##
##       NaN
##       NaN
##       NaN
##       NaN
##
## Covariances:
##                    Estimate  Std.Err  z-value  P(>|z|)   Std.lv  Std.all
##   f1 ~~
##     f2                0.104    0.021    4.924    0.000    0.366    0.366
##     f3                0.233    0.033    7.057    0.000    0.595    0.595
##   f2 ~~
##     f3                0.199    0.034    5.923    0.000    0.416    0.416
##   f1 ~~
##     f1b               0.000                                 NaN      NaN
##   f2 ~~
##     f1b               0.000                                 NaN      NaN
##   f3 ~~
##     f1b               0.000                                 NaN      NaN
##   f1b ~~
##     f2b               0.000                                 NaN      NaN
##     f3b               0.000                                 NaN      NaN
```

```
##   f1 ~~
##     f2b              0.000                                       NaN      NaN
##   f2 ~~
##     f2b              0.000                                       NaN      NaN
##   f3 ~~
##     f2b              0.000                                       NaN      NaN
##   f2b ~~
##     f3b              0.000                                       NaN      NaN
##   f1 ~~
##     f3b              0.000                                       NaN      NaN
##   f2 ~~
##     f3b              0.000                                       NaN      NaN
##   f3 ~~
##     f3b              0.000                                       NaN      NaN
##
## Variances:
##                    Estimate  Std.Err  z-value  P(>|z|)   Std.lv  Std.all
##    .Atnmy_PE_1 (j)    0.570    0.044   13.090    0.000    0.570    0.710
##    .Atnmy_PE_2 (k)    0.517    0.047   11.079    0.000    0.517    0.482
##    .Atnmy_PE_3 (l)    0.797    0.065   12.352    0.000    0.797    0.599
##    .Atnmy_PE_4 (m)    0.244    0.040    6.091    0.000    0.244    0.242
##    .Rltdn_PE_1 (n)    0.658    0.052   12.736    0.000    0.658    0.655
##    .Rltdn_PE_2 (o)    0.229    0.057    4.049    0.000    0.229    0.197
##    .Rltdn_PE_3 (p)    0.500    0.056    8.879    0.000    0.500    0.402
##    .Cmptn_PE_1 (r)    0.259    0.029    8.850    0.000    0.259    0.283
##    .Cmptn_PE_2 (s)    0.446    0.043   10.376    0.000    0.446    0.358
##    .Cmptn_PE_3 (t)    0.534    0.043   12.416    0.000    0.534    0.541
##    .Cmptn_PE_4 (u)    0.397    0.035   11.213    0.000    0.397    0.415
##     f1                0.233    0.043    5.383    0.000    1.000    1.000
##     f2                0.346    0.058    5.955    0.000    1.000    1.000
##     f3                0.658    0.066    9.928    0.000    1.000    1.000
##     Atnmy_PE_1        0.000                                 NaN      NaN
##     Atnmy_PE_2        0.000                                 NaN      NaN
##     Atnmy_PE_3        0.000                                 NaN      NaN
##     Atnmy_PE_4        0.000                                 NaN      NaN
##     Rltdn_PE_1        0.000                                 NaN      NaN
##     Rltdn_PE_2        0.000                                 NaN      NaN
##     Rltdn_PE_3        0.000                                 NaN      NaN
##     Cmptn_PE_1        0.000                                 NaN      NaN
##     Cmptn_PE_2        0.000                                 NaN      NaN
##     Cmptn_PE_3        0.000                                 NaN      NaN
##     Cmptn_PE_4        0.000                                 NaN      NaN
##     f1b               0.000                                 NaN      NaN
##     f2b               0.000                                 NaN      NaN
##     f3b               0.000                                 NaN      NaN
##
##
## Group 2 [between]:
##
## Latent Variables:
##                        Estimate  Std.Err  z-value  P(>|z|)   Std.lv
##   f1 =~
##     Atn_PE_1              0.000                               0.000
##     Atn_PE_2              0.000                               0.000
##     Atn_PE_3              0.000                               0.000
##     Atn_PE_4              0.000                               0.000
##   f2 =~
##     Rlt_PE_1              0.000                               0.000
##     Rlt_PE_2              0.000                               0.000
##     Rlt_PE_3              0.000                               0.000
```

```
##   f3 =~
##     Cmp_PE_1             0.000                               0.000
##     Cmp_PE_2             0.000                               0.000
##     Cmp_PE_3             0.000                               0.000
##     Cmp_PE_4             0.000                               0.000
##   Autonomy_PE_1b =~
##     Atn_PE_1 (sqc)       1.000                               1.750
##   Autonomy_PE_2b =~
##     Atn_PE_2 (sqc)       1.000                               1.173
##   Autonomy_PE_3b =~
##     Atn_PE_3 (sqc)       1.000                               1.253
##   Autonomy_PE_4b =~
##     Atn_PE_4 (sqc)       1.000                               1.490
##   Relatedness_PE_1b =~
##     Rlt_PE_1 (sqc)       1.000                               0.677
##   Relatedness_PE_2b =~
##     Rlt_PE_2 (sqc)       1.000                               0.754
##   Relatedness_PE_3b =~
##     Rlt_PE_3 (sqc)       1.000                               0.235
##   Competence_PE_1b =~
##     Cmp_PE_1 (sqc)       1.000                               0.825
##   Competence_PE_2b =~
##     Cmp_PE_2 (sqc)       1.000                               0.853
##   Competence_PE_3b =~
##     Cmp_PE_3 (sqc)       1.000                               0.844
##   Competence_PE_4b =~
##     Cmp_PE_4 (sqc)       1.000                               0.781
##   f1b =~
##     Atn_PE_1             1.000                               0.809
##     Atn_PE_2             0.878    0.163    5.381    0.000    1.060
##     Atn_PE_3             0.956    0.183    5.228    0.000    1.080
##     Atn_PE_4             1.010    0.186    5.429    0.000    0.961
##   f2b =~
##     Rlt_PE_1             1.000                               1.005
##     Rlt_PE_2             1.256    0.369    3.401    0.001    1.134
##     Rlt_PE_3             0.734    0.217    3.386    0.001    2.124
##   f3b =~
##     Cmp_PE_1             1.000                               0.890
##     Cmp_PE_2             1.095    0.283    3.873    0.000    0.942
##     Cmp_PE_3             1.058    0.289    3.658    0.000    0.920
##     Cmp_PE_4             0.931    0.259    3.590    0.000    0.876
##   Std.all
##
##     0.000
##     0.000
##     0.000
##     0.000
##
##     0.000
##     0.000
##     0.000
##
##     0.000
##     0.000
##     0.000
##     0.000
##
##     0.918
##
##     0.853
```

```
##
##     0.814
##
##     0.949
##
##     0.641
##
##     0.844
##
##     0.316
##
##     0.851
##
##     0.787
##
##     0.756
##
##     0.778
##
##     0.809
##     1.060
##     1.080
##     0.961
##
##     1.005
##     1.134
##     2.124
##
##     0.890
##     0.942
##     0.920
##     0.876
##
## Covariances:
##                    Estimate  Std.Err  z-value  P(>|z|)   Std.lv  Std.all
##   f1 ~~
##     f2                0.000                                  NaN      NaN
##     f3                0.000                                  NaN      NaN
##   f2 ~~
##     f3                0.000                                  NaN      NaN
##   f1 ~~
##     f1b               0.000                                  NaN      NaN
##   f2 ~~
##     f1b               0.000                                  NaN      NaN
##   f3 ~~
##     f1b               0.000                                  NaN      NaN
##   f1b ~~
##     f2b               0.192    0.193    0.993    0.321    0.199    0.199
##     f3b               0.584    0.263    2.219    0.026    0.561    0.561
##   f1 ~~
##     f2b               0.000                                  NaN      NaN
##   f2 ~~
##     f2b               0.000                                  NaN      NaN
##   f3 ~~
##     f2b               0.000                                  NaN      NaN
##   f2b ~~
##     f3b               0.206    0.124    1.665    0.096    0.413    0.413
##   f1 ~~
##     f3b               0.000                                  NaN      NaN
##   f2 ~~
```

```
##     f3b               0.000                               NaN      NaN
##   f3 ~~
##     f3b               0.000                               NaN      NaN
##
## Variances:
##                    Estimate  Std.Err  z-value  P(>|z|)   Std.lv  Std.all
##    .Atnmy_PE_1 (j)    0.570    0.044   13.090    0.000    0.570    0.157
##    .Atnmy_PE_2 (k)    0.517    0.047   11.079    0.000    0.517    0.273
##    .Atnmy_PE_3 (l)    0.797    0.065   12.352    0.000    0.797    0.337
##    .Atnmy_PE_4 (m)    0.244    0.040    6.091    0.000    0.244    0.099
##    .Rltdn_PE_1 (n)    0.658    0.052   12.736    0.000    0.658    0.589
##    .Rltdn_PE_2 (o)    0.229    0.057    4.049    0.000    0.229    0.288
##    .Rltdn_PE_3 (p)    0.500    0.056    8.879    0.000    0.500    0.900
##    .Cmptn_PE_1 (r)    0.259    0.029    8.850    0.000    0.259    0.276
##    .Cmptn_PE_2 (s)    0.446    0.043   10.376    0.000    0.446    0.380
##    .Cmptn_PE_3 (t)    0.534    0.043   12.416    0.000    0.534    0.428
##    .Cmptn_PE_4 (u)    0.397    0.035   11.213    0.000    0.397    0.394
##     f1                0.000                               NaN      NaN
##     f2                0.000                               NaN      NaN
##     f3                0.000                               NaN      NaN
##     Atnmy_PE_1        1.056    0.441    2.396    0.017    0.345    0.345
##     Atnmy_PE_2       -0.171    0.136   -1.261    0.207   -0.125   -0.125
##     Atnmy_PE_3       -0.263    0.187   -1.403    0.161   -0.167   -0.167
##     Atnmy_PE_4        0.170    0.166    1.025    0.305    0.077    0.077
##     Rltdn_PE_1       -0.005    0.196   -0.023    0.982   -0.010   -0.010
##     Rltdn_PE_2       -0.162    0.164   -0.990    0.322   -0.285   -0.285
##     Rltdn_PE_3       -0.194    0.108   -1.798    0.072   -3.510   -3.510
##     Cmptn_PE_1        0.142    0.140    1.013    0.311    0.208    0.208
##     Cmptn_PE_2        0.081    0.179    0.454    0.650    0.112    0.112
##     Cmptn_PE_3        0.109    0.201    0.545    0.586    0.153    0.153
##     Cmptn_PE_4        0.141    0.165    0.856    0.392    0.232    0.232
##     f1b               2.007    0.820    2.448    0.014    1.000    1.000
##     f2b               0.463    0.244    1.898    0.058    1.000    1.000
##     f3b               0.539    0.230    2.347    0.019    1.000    1.000
```

Path diagramm

```
semPlot::semPaths(models$mod_3x3_sep_fit,"std",edge.label.cex=0.5, curvePivot = TRUE)
```

```
## Warning in qgraph::qgraph(Edgelist, labels = nLab, bidirectional = Bidir, :
## Non-finite weights are omitted

## Warning in qgraph::qgraph(Edgelist, labels = nLab, bidirectional = Bidir, :
## Non-finite weights are omitted
```

1

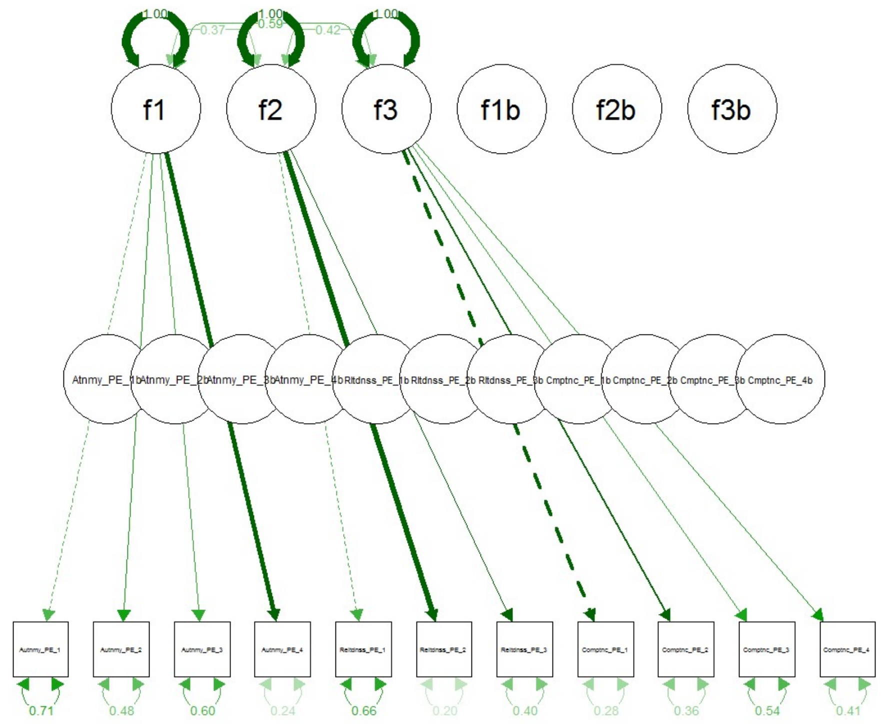

2

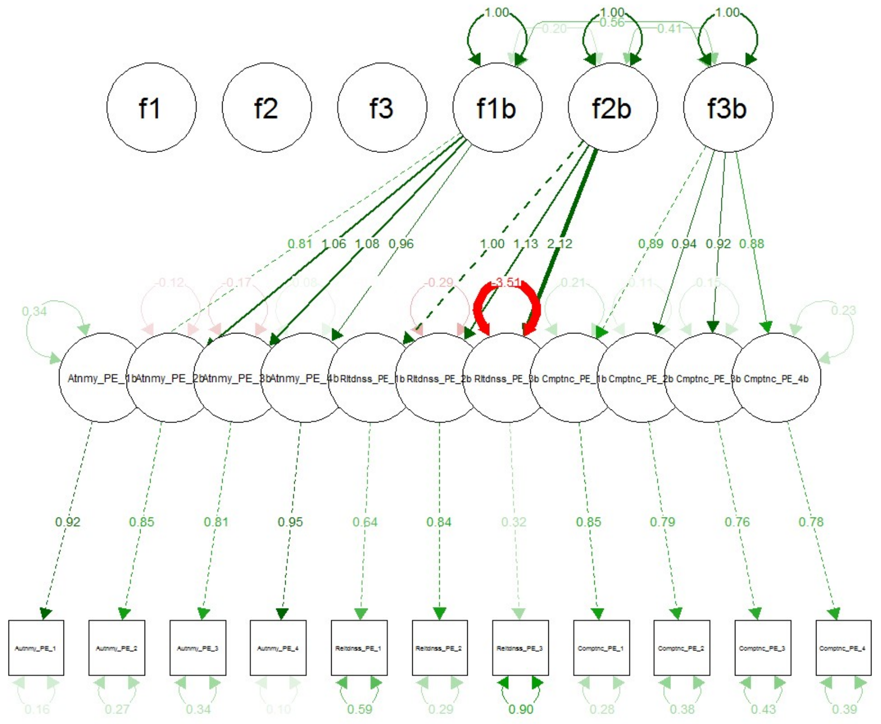

3x3, separate, manifest variances fixed where necessary

Model C

```
mod_3x3_sep_var <- '
f1 =~ c(1,0)*Autonomy_PE_1 + c(NA,0)*Autonomy_PE_2 + c(NA,0)*Autonomy_PE_3 + c(NA,0)*Autonom
y_PE_4
f2 =~ c(1,0)*Relatedness_PE_1 + c(NA,0)*Relatedness_PE_2 + c(NA,0)*Relatedness_PE_3
f3 =~ c(1,0)*Competence_PE_1 + c(NA,0)*Competence_PE_2 + c(NA,0)*Competence_PE_3 + c(NA,0)*C
ompetence_PE_4
Autonomy_PE_1 ~~ c(j,j)*Autonomy_PE_1
Autonomy_PE_2 ~~ c(NA,0)*Autonomy_PE_2
Autonomy_PE_3 ~~ c(NA,0)*Autonomy_PE_3
Autonomy_PE_4 ~~ c(m,m)*Autonomy_PE_4
Relatedness_PE_1 ~~ c(NA,0)*Relatedness_PE_1
Relatedness_PE_2 ~~ c(NA,0)*Relatedness_PE_2
Relatedness_PE_3 ~~ c(NA,0)*Relatedness_PE_3
Competence_PE_1 ~~ c(r,r)*Competence_PE_1
Competence_PE_2 ~~ c(s,s)*Competence_PE_2
Competence_PE_3 ~~ c(t,t)*Competence_PE_3
Competence_PE_4 ~~ c(u,u)*Competence_PE_4
f1 ~~ c(NA,0)*f1
f2 ~~ c(NA,0)*f2
f3 ~~ c(NA,0)*f3
f1 ~~ c(NA,0)*f2
f1 ~~ c(NA,0)*f3
f2 ~~ c(NA,0)*f3
Autonomy_PE_1b =~ c(0,x$sqc)*Autonomy_PE_1
Autonomy_PE_1b ~~ c(0,NA)*Autonomy_PE_1b
Autonomy_PE_2b =~ c(0,x$sqc)*Autonomy_PE_2
Autonomy_PE_2b ~~ c(0,NA)*Autonomy_PE_2b
Autonomy_PE_3b =~ c(0,x$sqc)*Autonomy_PE_3
Autonomy_PE_3b ~~ c(0,NA)*Autonomy_PE_3b
Autonomy_PE_4b =~ c(0,x$sqc)*Autonomy_PE_4
Autonomy_PE_4b ~~ c(0,NA)*Autonomy_PE_4b
Relatedness_PE_1b =~ c(0,x$sqc)*Relatedness_PE_1
Relatedness_PE_1b ~~ c(0,NA)*Relatedness_PE_1b
Relatedness_PE_2b =~ c(0,x$sqc)*Relatedness_PE_2
Relatedness_PE_2b ~~ c(0,NA)*Relatedness_PE_2b
Relatedness_PE_3b =~ c(0,x$sqc)*Relatedness_PE_3
Relatedness_PE_3b ~~ c(0,NA)*Relatedness_PE_3b
Competence_PE_1b =~ c(0,x$sqc)*Competence_PE_1
Competence_PE_1b ~~ c(0,NA)*Competence_PE_1b
Competence_PE_2b =~ c(0,x$sqc)*Competence_PE_2
Competence_PE_2b ~~ c(0,NA)*Competence_PE_2b
Competence_PE_3b =~ c(0,x$sqc)*Competence_PE_3
Competence_PE_3b ~~ c(0,NA)*Competence_PE_3b
Competence_PE_4b =~ c(0,x$sqc)*Competence_PE_4
Competence_PE_4b ~~ c(0,NA)*Competence_PE_4b
f1b =~ c(0,1)*Autonomy_PE_1b + c(0,NA)*Autonomy_PE_2b + c(0,NA)*Autonomy_PE_3b + c(0,NA)*Aut
onomy_PE_4b
f2b =~ c(0,1)*Relatedness_PE_1b + c(0,NA)*Relatedness_PE_2b + c(0,NA)*Relatedness_PE_3b
f3b =~ c(0,1)*Competence_PE_1b + c(0,NA)*Competence_PE_2b + c(0,NA)*Competence_PE_3b + c(0,N
A)*Competence_PE_4b
f1b ~~ c(0,NA)*f1b + c(0,0)*f1 + c(0,0)*f2 + c(0,0)*f3 + c(0,NA)*f2b + c(0,NA)*f3b
f2b ~~ c(0,NA)*f2b + c(0,0)*f1 + c(0,0)*f2 + c(0,0)*f3 + c(0,NA)*f3b
f3b ~~ c(0,NA)*f3b + c(0,0)*f1 + c(0,0)*f2 + c(0,0)*f3
'

# Fit hypothesized model
models$mod_3x3_sep_var_fit<- cfa(mod_3x3_sep_var, sample.cov = combined.cov, sample.nobs = c
ombined.n, orthogonal = T)
# Summary
summary(models$mod_3x3_sep_var_fit, fit.measures = T, standardized = T)
```

```
## lavaan 0.6-3 ended normally after 86 iterations
##
##   Optimization method                           NLMINB
##   Number of free parameters                         56
##   Number of equality constraints                     6
##
##   Number of observations per group
##   within                                           400
##   between                                           33
##
##   Estimator                                         ML
##   Model Fit Test Statistic                     186.377
##   Degrees of freedom                                82
##   P-value (Chi-square)                           0.000
##
## Chi-square for each group:
##
##   within                                       124.159
##   between                                       62.218
##
## Model test baseline model:
##
##   Minimum Function Test Statistic             2130.224
##   Degrees of freedom                               110
##   P-value                                        0.000
##
## User model versus baseline model:
##
##   Comparative Fit Index (CFI)                    0.948
##   Tucker-Lewis Index (TLI)                       0.931
##
## Loglikelihood and Information Criteria:
##
##   Loglikelihood user model (H0)              -5960.555
##   Loglikelihood unrestricted model (H1)      -5867.367
##
##   Number of free parameters                         50
##   Akaike (AIC)                               12021.111
##   Bayesian (BIC)                             12224.647
##   Sample-size adjusted Bayesian (BIC)        12065.975
##
## Root Mean Square Error of Approximation:
##
##   RMSEA                                          0.077
##   90 Percent Confidence Interval          0.062  0.091
##   P-value RMSEA <= 0.05                          0.002
##
## Standardized Root Mean Square Residual:
##
##   SRMR                                           0.058
##
## Parameter Estimates:
##
##   Information                                 Expected
##   Information saturated (h1) model          Structured
##   Standard Errors                             Standard
##
##
## Group 1 [within]:
##
```

```
## Latent Variables:
##                        Estimate  Std.Err  z-value  P(>|z|)   Std.lv
##   f1 =~
##     Atnmy_PE_1            1.000                                0.482
##     Atnmy_PE_2            1.545    0.157    9.842    0.000    0.745
##     Atnmy_PE_3            1.514    0.165    9.155    0.000    0.730
##     Atnmy_PE_4            1.815    0.174   10.446    0.000    0.876
##   f2 =~
##     Rltdn_PE_1            1.000                                0.589
##     Rltdn_PE_2            1.642    0.148   11.120    0.000    0.966
##     Rltdn_PE_3            1.466    0.130   11.258    0.000    0.863
##   f3 =~
##     Cmptn_PE_1            1.000                                0.811
##     Cmptn_PE_2            1.102    0.063   17.612    0.000    0.894
##     Cmptn_PE_3            0.829    0.058   14.299    0.000    0.672
##     Cmptn_PE_4            0.922    0.055   16.662    0.000    0.748
##   Autonomy_PE_1b =~
##     Atnmy_PE_1 (x)        1.000                                0.000
##   Autonomy_PE_2b =~
##     Atnmy_PE_2 (x)        1.000                                0.000
##   Autonomy_PE_3b =~
##     Atnmy_PE_3 (x)        1.000                                0.000
##   Autonomy_PE_4b =~
##     Atnmy_PE_4 (x)        1.000                                0.000
##   Relatedness_PE_1b =~
##     Rltdn_PE_1 (x)        1.000                                0.000
##   Relatedness_PE_2b =~
##     Rltdn_PE_2 (x)        1.000                                0.000
##   Relatedness_PE_3b =~
##     Rltdn_PE_3 (x)        1.000                                0.000
##   Competence_PE_1b =~
##     Cmptn_PE_1 (x)        1.000                                0.000
##   Competence_PE_2b =~
##     Cmptn_PE_2 (x)        1.000                                0.000
##   Competence_PE_3b =~
##     Cmptn_PE_3 (x)        1.000                                0.000
##   Competence_PE_4b =~
##     Cmptn_PE_4 (x)        1.000                                0.000
##   f1b =~
##     Atnmy_PE_1            0.000                                  NaN
##     Atnmy_PE_2            0.000                                  NaN
##     Atnmy_PE_3            0.000                                  NaN
##     Atnmy_PE_4            0.000                                  NaN
##   f2b =~
##     Rltdn_PE_1            0.000                                  NaN
##     Rltdn_PE_2            0.000                                  NaN
##     Rltdn_PE_3            0.000                                  NaN
##   f3b =~
##     Cmptn_PE_1            0.000                                  NaN
##     Cmptn_PE_2            0.000                                  NaN
##     Cmptn_PE_3            0.000                                  NaN
##     Cmptn_PE_4            0.000                                  NaN
##   Std.all
##
##     0.538
##     0.720
##     0.633
##     0.871
##
##     0.587
```

```
##     0.896
##     0.773
##
##     0.847
##     0.801
##     0.677
##     0.765
##
##     0.000
##
##     0.000
##
##     0.000
##
##     0.000
##
##     0.000
##
##     0.000
##
##     0.000
##
##     0.000
##
##     0.000
##
##     0.000
##
##     0.000
##
##       NaN
##       NaN
##       NaN
##       NaN
##
##       NaN
##       NaN
##       NaN
##
##       NaN
##       NaN
##       NaN
##       NaN
##
## Covariances:
##                    Estimate  Std.Err  z-value  P(>|z|)   Std.lv  Std.all
##   f1 ~~
##     f2                 0.104    0.021    4.924    0.000    0.366    0.366
##     f3                 0.233    0.033    7.057    0.000    0.595    0.595
##   f2 ~~
##     f3                 0.199    0.034    5.923    0.000    0.416    0.416
##   f1 ~~
##     f1b                0.000                                 NaN      NaN
##   f2 ~~
##     f1b                0.000                                 NaN      NaN
##   f3 ~~
##     f1b                0.000                                 NaN      NaN
##   f1b ~~
##     f2b                0.000                                 NaN      NaN
##     f3b                0.000                                 NaN      NaN
```

```
##   f1 ~~
##     f2b              0.000                               NaN      NaN
##   f2 ~~
##     f2b              0.000                               NaN      NaN
##   f3 ~~
##     f2b              0.000                               NaN      NaN
##   f2b ~~
##     f3b              0.000                               NaN      NaN
##   f1 ~~
##     f3b              0.000                               NaN      NaN
##   f2 ~~
##     f3b              0.000                               NaN      NaN
##   f3 ~~
##     f3b              0.000                               NaN      NaN
##
## Variances:
##                    Estimate  Std.Err  z-value  P(>|z|)   Std.lv  Std.all
##    .Atnmy_PE_1 (j)    0.570    0.044   13.090    0.000    0.570    0.710
##    .Atnmy_PE_2        0.517    0.047   11.079    0.000    0.517    0.482
##    .Atnmy_PE_3        0.797    0.065   12.352    0.000    0.797    0.599
##    .Atnmy_PE_4 (m)    0.244    0.040    6.091    0.000    0.244    0.242
##    .Rltdn_PE_1        0.658    0.052   12.736    0.000    0.658    0.655
##    .Rltdn_PE_2        0.229    0.057    4.049    0.000    0.229    0.197
##    .Rltdn_PE_3        0.500    0.056    8.879    0.000    0.500    0.402
##    .Cmptn_PE_1 (r)    0.259    0.029    8.850    0.000    0.259    0.283
##    .Cmptn_PE_2 (s)    0.446    0.043   10.376    0.000    0.446    0.358
##    .Cmptn_PE_3 (t)    0.534    0.043   12.416    0.000    0.534    0.541
##    .Cmptn_PE_4 (u)    0.397    0.035   11.213    0.000    0.397    0.415
##     f1                0.233    0.043    5.383    0.000    1.000    1.000
##     f2                0.346    0.058    5.955    0.000    1.000    1.000
##     f3                0.658    0.066    9.928    0.000    1.000    1.000
##     Atnmy_PE_1        0.000                                 NaN      NaN
##     Atnmy_PE_2        0.000                                 NaN      NaN
##     Atnmy_PE_3        0.000                                 NaN      NaN
##     Atnmy_PE_4        0.000                                 NaN      NaN
##     Rltdn_PE_1        0.000                                 NaN      NaN
##     Rltdn_PE_2        0.000                                 NaN      NaN
##     Rltdn_PE_3        0.000                                 NaN      NaN
##     Cmptn_PE_1        0.000                                 NaN      NaN
##     Cmptn_PE_2        0.000                                 NaN      NaN
##     Cmptn_PE_3        0.000                                 NaN      NaN
##     Cmptn_PE_4        0.000                                 NaN      NaN
##     f1b               0.000                                 NaN      NaN
##     f2b               0.000                                 NaN      NaN
##     f3b               0.000                                 NaN      NaN
##
##
## Group 2 [between]:
##
## Latent Variables:
##                        Estimate  Std.Err  z-value  P(>|z|)   Std.lv
##   f1 =~
##     Atn_PE_1              0.000                               0.000
##     Atn_PE_2              0.000                               0.000
##     Atn_PE_3              0.000                               0.000
##     Atn_PE_4              0.000                               0.000
##   f2 =~
##     Rlt_PE_1              0.000                               0.000
##     Rlt_PE_2              0.000                               0.000
##     Rlt_PE_3              0.000                               0.000
```

```
##    f3 =~
##      Cmp_PE_1             0.000                               0.000
##      Cmp_PE_2             0.000                               0.000
##      Cmp_PE_3             0.000                               0.000
##      Cmp_PE_4             0.000                               0.000
##    Autonomy_PE_1b =~
##      Atn_PE_1 (sqc)       1.000                               1.750
##    Autonomy_PE_2b =~
##      Atn_PE_2 (sqc)       1.000                               1.375
##    Autonomy_PE_3b =~
##      Atn_PE_3 (sqc)       1.000                               1.539
##    Autonomy_PE_4b =~
##      Atn_PE_4 (sqc)       1.000                               1.490
##    Relatedness_PE_1b =~
##      Rlt_PE_1 (sqc)       1.000                               1.057
##    Relatedness_PE_2b =~
##      Rlt_PE_2 (sqc)       1.000                               0.893
##    Relatedness_PE_3b =~
##      Rlt_PE_3 (sqc)       1.000                               0.745
##    Competence_PE_1b =~
##      Cmp_PE_1 (sqc)       1.000                               0.825
##    Competence_PE_2b =~
##      Cmp_PE_2 (sqc)       1.000                               0.853
##    Competence_PE_3b =~
##      Cmp_PE_3 (sqc)       1.000                               0.844
##    Competence_PE_4b =~
##      Cmp_PE_4 (sqc)       1.000                               0.781
##    f1b =~
##      Atn_PE_1             1.000                               0.809
##      Atn_PE_2             0.878    0.163    5.381    0.000    0.904
##      Atn_PE_3             0.956    0.183    5.228    0.000    0.880
##      Atn_PE_4             1.010    0.186    5.429    0.000    0.961
##    f2b =~
##      Rlt_PE_1             1.000                               0.644
##      Rlt_PE_2             1.256    0.369    3.401    0.001    0.957
##      Rlt_PE_3             0.734    0.217    3.386    0.001    0.670
##    f3b =~
##      Cmp_PE_1             1.000                               0.890
##      Cmp_PE_2             1.095    0.283    3.873    0.000    0.942
##      Cmp_PE_3             1.058    0.289    3.658    0.000    0.920
##      Cmp_PE_4             0.931    0.259    3.590    0.000    0.876
##    Std.all
##
##      0.000
##      0.000
##      0.000
##      0.000
##
##      0.000
##      0.000
##      0.000
##
##      0.000
##      0.000
##      0.000
##      0.000
##
##      0.918
##
##      1.000
```

```
##
##     1.000
##
##     0.949
##
##     1.000
##
##     1.000
##
##     1.000
##
##     0.851
##
##     0.787
##
##     0.756
##
##     0.778
##
##     0.809
##     0.904
##     0.880
##     0.961
##
##     0.644
##     0.957
##     0.670
##
##     0.890
##     0.942
##     0.920
##     0.876
##
## Covariances:
##                    Estimate  Std.Err  z-value  P(>|z|)   Std.lv  Std.all
##   f1 ~~
##     f2                0.000                                NaN      NaN
##     f3                0.000                                NaN      NaN
##   f2 ~~
##     f3                0.000                                NaN      NaN
##   f1 ~~
##     f1b               0.000                                NaN      NaN
##   f2 ~~
##     f1b               0.000                                NaN      NaN
##   f3 ~~
##     f1b               0.000                                NaN      NaN
##   f1b ~~
##     f2b               0.192    0.193    0.993    0.321    0.199    0.199
##     f3b               0.584    0.263    2.219    0.027    0.561    0.561
##   f1 ~~
##     f2b               0.000                                NaN      NaN
##   f2 ~~
##     f2b               0.000                                NaN      NaN
##   f3 ~~
##     f2b               0.000                                NaN      NaN
##   f2b ~~
##     f3b               0.206    0.124    1.665    0.096    0.413    0.413
##   f1 ~~
##     f3b               0.000                                NaN      NaN
##   f2 ~~
```

```
##     f3b              0.000                                    NaN      NaN
##   f3 ~~
##     f3b              0.000                                    NaN      NaN
##
## Variances:
##                    Estimate  Std.Err  z-value  P(>|z|)   Std.lv  Std.all
##    .Atnmy_PE_1 (j)    0.570    0.044   13.090    0.000    0.570    0.157
##    .Atnmy_PE_2        0.000                               0.000    0.000
##    .Atnmy_PE_3        0.000                               0.000    0.000
##    .Atnmy_PE_4 (m)    0.244    0.040    6.091    0.000    0.244    0.099
##    .Rltdn_PE_1        0.000                               0.000    0.000
##    .Rltdn_PE_2        0.000                               0.000    0.000
##    .Rltdn_PE_3        0.000                               0.000    0.000
##    .Cmptn_PE_1 (r)    0.259    0.029    8.850    0.000    0.259    0.276
##    .Cmptn_PE_2 (s)    0.446    0.043   10.376    0.000    0.446    0.380
##    .Cmptn_PE_3 (t)    0.534    0.043   12.416    0.000    0.534    0.428
##    .Cmptn_PE_4 (u)    0.397    0.035   11.213    0.000    0.397    0.394
##     f1               0.000                                    NaN      NaN
##     f2               0.000                                    NaN      NaN
##     f3               0.000                                    NaN      NaN
##     Atnmy_PE_1       1.056    0.441    2.396    0.017    0.345    0.345
##     Atnmy_PE_2       0.345    0.128    2.707    0.007    0.183    0.183
##     Atnmy_PE_3       0.534    0.176    3.037    0.002    0.226    0.226
##     Atnmy_PE_4       0.170    0.166    1.025    0.305    0.077    0.077
##     Rltdn_PE_1       0.654    0.189    3.458    0.001    0.585    0.585
##     Rltdn_PE_2       0.067    0.153    0.440    0.660    0.085    0.085
##     Rltdn_PE_3       0.306    0.092    3.319    0.001    0.551    0.551
##     Cmptn_PE_1       0.142    0.140    1.013    0.311    0.208    0.208
##     Cmptn_PE_2       0.081    0.179    0.454    0.650    0.112    0.112
##     Cmptn_PE_3       0.109    0.201    0.545    0.586    0.153    0.153
##     Cmptn_PE_4       0.141    0.165    0.856    0.392    0.232    0.232
##     f1b              2.007    0.820    2.448    0.014    1.000    1.000
##     f2b              0.463    0.244    1.898    0.058    1.000    1.000
##     f3b              0.539    0.230    2.347    0.019    1.000    1.000
```

see fit indices of model C in table 2 ** Factor loadings in table 3**

Path diagramm

```
semPlot::semPaths(models$mod_3x3_sep_var_fit,"std",edge.label.cex=0.5, curvePivot = TRUE)
```

```
## Warning in qgraph::qgraph(Edgelist, labels = nLab, bidirectional = Bidir, :
## Non-finite weights are omitted

## Warning in qgraph::qgraph(Edgelist, labels = nLab, bidirectional = Bidir, :
## Non-finite weights are omitted
```

1

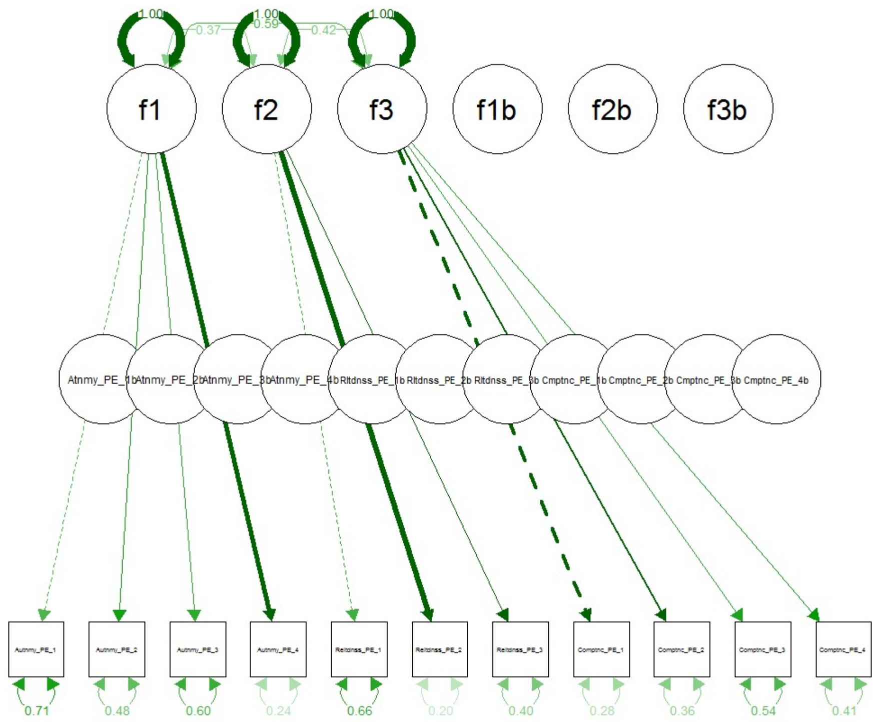

2

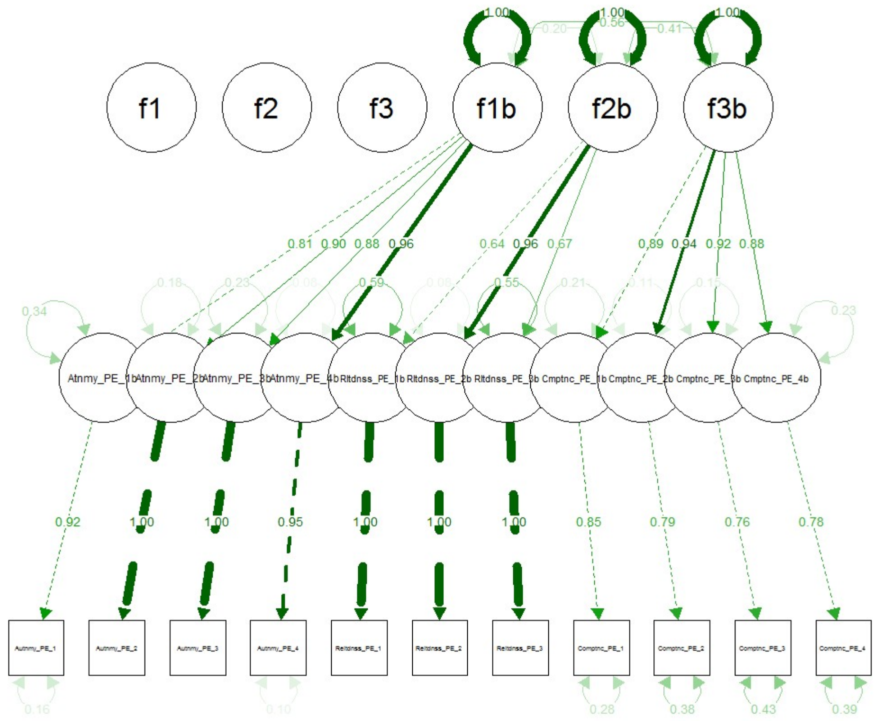

Combine all converging models into table and see a subset of fit measures to compare the indices

```
overview <- round(sapply(models, function(X) fitmeasures(X, c("npar", "chisq", "df", "cfi",
"tli", "rmsea", "rmsea.ci.lower", "rmsea.ci.upper", "srmr", "aic"))),3)
overview
```

```
##                mod_one_fac mod_three_fac nullmodel_fit mod_ind_fit
## npar                22.000        25.000        25.000      35.000
## chisq              650.536       124.159       273.515     244.328
## df                  44.000        41.000       107.000      97.000
## cfi                  0.667         0.954         0.918       0.927
## tli                  0.584         0.939         0.915       0.917
## rmsea                0.186         0.071         0.085       0.084
## rmsea.ci.lower       0.173         0.057         0.072       0.071
## rmsea.ci.upper       0.198         0.086         0.097       0.097
## srmr                 0.117         0.054         0.091       0.136
## aic              11541.438     11021.062     12058.249   12049.061
##                mod_sat_fit mod_3x1_fit mod_3x1_var_fit
## npar                90.000      46.000          46.000
## chisq              142.171     209.600         209.600
## df                  42.000      86.000          86.000
## cfi                  0.950       0.939           0.939
## tli                  0.870       0.922           0.922
## rmsea                0.105       0.081           0.081
## rmsea.ci.lower       0.086       0.068           0.068
## rmsea.ci.upper       0.124       0.096           0.096
## srmr                 0.104       0.119           0.119
## aic              12056.904   12036.333       12036.333
##                mod_3x1_free_vareq_fit mod_3x1_free_vareq_var_fit
## npar                           61.000                     61.000
## chisq                         157.964                    157.964
## df                             71.000                     71.000
## cfi                             0.957                      0.957
## tli                             0.933                      0.933
## rmsea                           0.075                      0.075
## rmsea.ci.lower                  0.059                      0.059
## rmsea.ci.upper                  0.091                      0.091
## srmr                            0.055                      0.055
## aic                         12014.698                  12014.698
##                mod_3x1_sep_fit mod_3x1_sep_var_fit mod_3x3_fit
## npar                    45.000              45.000      49.000
## chisq                  253.129             253.129     189.720
## df                      87.000              87.000      83.000
## cfi                      0.918               0.918       0.947
## tli                      0.896               0.896       0.930
## rmsea                    0.094               0.094       0.077
## rmsea.ci.lower           0.081               0.081       0.063
## rmsea.ci.upper           0.107               0.107       0.092
## srmr                     0.065               0.065       0.118
## aic                  12077.862           12077.862   12022.453
##                mod_3x3_var_fit mod_3x3_free_vareq_fit
## npar                    49.000                 64.000
## chisq                  189.720                146.746
## df                      83.000                 68.000
## cfi                      0.947                  0.961
## tli                      0.930                  0.937
## rmsea                    0.077                  0.073
## rmsea.ci.lower           0.063                  0.057
## rmsea.ci.upper           0.092                  0.089
## srmr                     0.118                  0.056
## aic                  12022.453              12009.480
##                mod_3x3_free_vareq_var_fit mod_3x3_sep_fit
## npar                               64.000          50.000
## chisq                             146.746         186.377
## df                                 68.000          82.000
## cfi                                 0.961           0.948
```

```
## tli                                  0.937          0.931
## rmsea                                0.073          0.077
## rmsea.ci.lower                       0.057          0.062
## rmsea.ci.upper                       0.089          0.091
## srmr                                 0.056          0.058
## aic                              12009.480      12021.111
##               mod_3x3_sep_var_fit
## npar                       50.000
## chisq                     186.377
## df                         82.000
## cfi                         0.948
## tli                         0.931
## rmsea                       0.077
## rmsea.ci.lower              0.062
## rmsea.ci.upper              0.091
## srmr                        0.058
## aic                     12021.111
```

Test differences of model fits

```
# Chi Square Difference Test
anova(models$nullmodel_fit, models$mod_ind_fit)
```

	Df <int>	AIC <dbl>	BIC <dbl>	Chisq <dbl>	Chisq diff <dbl>	Df diff <int>	Pr(>Chisq) <dbl>
models$mod_ind_fit	97	12049.06	12191.54	244.3276	NA	NA	NA
models$nullmodel_fit	107	12058.25	12160.02	273.5153	29.18766	10	0.001161778

2 rows

```
anova(models$mod_ind_fit, models$mod_sat_fit)
```

	Df <int>	AIC <dbl>	BIC <dbl>	Chisq <dbl>	Chisq diff <dbl>	Df diff <int>	Pr(>Chisq) <dbl>
models$mod_sat_fit	42	12056.90	12423.27	142.1710	NA	NA	NA
models$mod_ind_fit	97	12049.06	12191.54	244.3276	102.1566	55	0.0001167777

2 rows

```
anova(models$mod_three_fac, models$mod_3x3_sep_var_fit)
```

	Df <int>	AIC <dbl>	BIC <dbl>	Chisq <dbl>	Chisq diff <dbl>	Df diff <int>	Pr(>Chisq) <dbl>
models$mod_three_fac	41	11021.06	11120.85	124.1593	NA	NA	NA
models$mod_3x3_sep_var_fit	82	12021.11	12224.65	186.3772	62.21789	41	0.01785366

2 rows

```
anova(models$mod_3x1_var_fit, models$mod_3x3_sep_var_fit)
```

	Df <int>	AIC <dbl>	BIC <dbl>	Chisq <dbl>	Chisq diff <dbl>	Df diff <int>	Pr(>Chisq) <dbl>

	Df	AIC	BIC	Chisq	Chisq diff	Df diff	Pr(>Chisq)
	<int>	<dbl>	<dbl>	<dbl>	<dbl>	<int>	<dbl>
models$mod_3x3_sep_var_fit	82	12021.11	12224.65	186.3772	NA	NA	NA
models$mod_3x1_var_fit	86	12036.33	12223.59	209.5997	23.22259	4	0.000114298

2 rows

```
anova(models$mod_3x1_sep_var_fit, models$mod_3x3_sep_var_fit)
```

	Df	AIC	BIC	Chisq	Chisq diff	Df diff	Pr(>Chisq)
	<int>	<dbl>	<dbl>	<dbl>	<dbl>	<int>	<dbl>
models$mod_3x3_sep_var_fit	82	12021.11	12224.65	186.3772	NA	NA	NA
models$mod_3x1_sep_var_fit	87	12077.86	12261.05	253.1289	66.75172	5	4.85208e-13

2 rows

Reliability

```
x$ab.cov
```

```
##                 Autonomy_PE_1 Autonomy_PE_2 Autonomy_PE_3 Autonomy_PE_4
## Autonomy_PE_1     0.2254660370   0.110041888   0.129882792   0.125317946
## Autonomy_PE_2     0.1100418876   0.067123572   0.092198916   0.091947551
## Autonomy_PE_3     0.1298827917   0.092198916   0.084881247   0.103989004
## Autonomy_PE_4     0.1253179459   0.091947551   0.103989004   0.117008386
## Relatedness_PE_1  0.0004557546   0.011154914   0.013058193   0.008440437
## Relatedness_PE_2 -0.0215688141   0.001286220   0.007795585  -0.007891732
## Relatedness_PE_3 -0.0155121325  -0.003668756  -0.009609168  -0.011968075
## Competence_PE_1   0.0207874610   0.003979269   0.023246469  -0.008261176
## Competence_PE_2   0.0287635700   0.024944084   0.022987399   0.015401268
## Competence_PE_3   0.0209655811   0.037019698   0.037187500   0.036701265
## Competence_PE_4  -0.0052223562   0.003691356   0.013155642   0.004594645
##                 Relatedness_PE_1 Relatedness_PE_2 Relatedness_PE_3
## Autonomy_PE_1       0.0004557546     -0.021568814     -0.015512132
## Autonomy_PE_2       0.0111549142      0.001286220     -0.003668756
## Autonomy_PE_3       0.0130581933      0.007795585     -0.009609168
## Autonomy_PE_4       0.0084404374     -0.007891732     -0.011968075
## Relatedness_PE_1    0.0110719214      0.001738461     -0.010398825
## Relatedness_PE_2    0.0017384607     -0.026341136     -0.030565812
## Relatedness_PE_3   -0.0103988253     -0.030565812     -0.051696955
## Competence_PE_1    -0.0041897644     -0.006816801     -0.016217660
## Competence_PE_2    -0.0134527685     -0.014032045     -0.010806001
## Competence_PE_3     0.0384777481      0.017494043     -0.003063422
## Competence_PE_4    -0.0145234439     -0.003085762     -0.009944725
##                 Competence_PE_1 Competence_PE_2 Competence_PE_3
## Autonomy_PE_1       0.0207874610     0.028763570    0.0209655811
## Autonomy_PE_2       0.0039792690     0.024944084    0.0370196982
## Autonomy_PE_3       0.0232464687     0.022987399    0.0371874996
## Autonomy_PE_4      -0.0082611760     0.015401268    0.0367012652
## Relatedness_PE_1   -0.0041897644    -0.013452768    0.0384777481
## Relatedness_PE_2   -0.0068168011    -0.014032045    0.0174940433
## Relatedness_PE_3   -0.0162176599    -0.010806001   -0.0030634223
## Competence_PE_1     0.0038339171    -0.002428686    0.0008792136
## Competence_PE_2    -0.0024286857    -0.002926074   -0.0079702029
## Competence_PE_3     0.0008792136    -0.007970203    0.0227905380
## Competence_PE_4    -0.0069076756    -0.004585732    0.0049245767
##                 Competence_PE_4
## Autonomy_PE_1       -0.005222356
## Autonomy_PE_2        0.003691356
## Autonomy_PE_3        0.013155642
## Autonomy_PE_4        0.004594645
## Relatedness_PE_1    -0.014523444
## Relatedness_PE_2    -0.003085762
## Relatedness_PE_3    -0.009944725
## Competence_PE_1     -0.006907676
## Competence_PE_2     -0.004585732
## Competence_PE_3      0.004924577
## Competence_PE_4      0.006037650
```

```
x$pw.cov
```

```
##                   Autonomy_PE_1 Autonomy_PE_2 Autonomy_PE_3 Autonomy_PE_4
## Autonomy_PE_1       0.80503267     0.4275278     0.3438085     0.4098760
## Autonomy_PE_2       0.42752784     1.0750267     0.4778976     0.6658384
## Autonomy_PE_3       0.34380854     0.4778976     1.3335666     0.6409524
## Autonomy_PE_4       0.40987601     0.6658384     0.6409524     1.0140446
## Relatedness_PE_1    0.10565606     0.1773651     0.1644968     0.1927337
## Relatedness_PE_2    0.09208792     0.2032998     0.2986302     0.2923553
## Relatedness_PE_3    0.15243330     0.2248915     0.4111192     0.3547002
## Competence_PE_1     0.21328195     0.3492620     0.4217084     0.3964246
## Competence_PE_2     0.16498056     0.3521735     0.5657179     0.4406246
## Competence_PE_3     0.25023771     0.3161776     0.4881670     0.4411432
## Competence_PE_4     0.17408258     0.2597811     0.3481657     0.3708210
##                   Relatedness_PE_1 Relatedness_PE_2 Relatedness_PE_3
## Autonomy_PE_1            0.1056561       0.09208792        0.1524333
## Autonomy_PE_2            0.1773651       0.20329984        0.2248915
## Autonomy_PE_3            0.1644968       0.29863022        0.4111192
## Autonomy_PE_4            0.1927337       0.29235528        0.3547002
## Relatedness_PE_1         1.0070472       0.57598628        0.4701164
## Relatedness_PE_2         0.5759863       1.16610226        0.8409045
## Relatedness_PE_3         0.4701164       0.84090453        1.2474591
## Competence_PE_1          0.3203346       0.31994410        0.2641034
## Competence_PE_2          0.3221775       0.32151851        0.3153799
## Competence_PE_3          0.2190911       0.34112304        0.3701932
## Competence_PE_4          0.2687741       0.23473665        0.1945566
##                   Competence_PE_1 Competence_PE_2 Competence_PE_3
## Autonomy_PE_1           0.2132820       0.1649806       0.2502377
## Autonomy_PE_2           0.3492620       0.3521735       0.3161776
## Autonomy_PE_3           0.4217084       0.5657179       0.4881670
## Autonomy_PE_4           0.3964246       0.4406246       0.4411432
## Relatedness_PE_1        0.3203346       0.3221775       0.2190911
## Relatedness_PE_2        0.3199441       0.3215185       0.3411230
## Relatedness_PE_3        0.2641034       0.3153799       0.3701932
## Competence_PE_1         0.9198146       0.7405328       0.5170700
## Competence_PE_2         0.7405328       1.2493319       0.5849585
## Competence_PE_3         0.5170700       0.5849585       0.9884608
## Competence_PE_4         0.6180336       0.6683458       0.5179660
##                   Competence_PE_4
## Autonomy_PE_1           0.1740826
## Autonomy_PE_2           0.2597811
## Autonomy_PE_3           0.3481657
## Autonomy_PE_4           0.3708210
## Relatedness_PE_1        0.2687741
## Relatedness_PE_2        0.2347367
## Relatedness_PE_3        0.1945566
## Competence_PE_1         0.6180336
## Competence_PE_2         0.6683458
## Competence_PE_3         0.5179660
## Competence_PE_4         0.9589129
```

```
y_ab <- sqrt(x$ab.cov^2)
```

```
# Calculate the nearest positive definite matrices
pw_mat <- data.matrix(nearPD(x$pw.cov)$mat)
ab_mat <- data.matrix(nearPD(x$ab.cov)$mat)

# Combined
## Within
psych::alpha(pw_mat)
```

```
##
## Reliability analysis
## Call: psych::alpha(x = pw_mat)
##
##   raw_alpha std.alpha G6(smc) average_r S/N median_r
##       0.85      0.85    0.88      0.34 5.8     0.32
##
##  Reliability if an item is dropped:
##                  raw_alpha std.alpha G6(smc) average_r S/N var.r med.r
## Autonomy_PE_1         0.85      0.85    0.88      0.37 5.8 0.022  0.32
## Autonomy_PE_2         0.84      0.84    0.87      0.35 5.4 0.024  0.32
## Autonomy_PE_3         0.84      0.84    0.87      0.34 5.2 0.025  0.31
## Autonomy_PE_4         0.83      0.83    0.86      0.33 5.0 0.024  0.31
## Relatedness_PE_1      0.85      0.85    0.88      0.36 5.7 0.023  0.32
## Relatedness_PE_2      0.84      0.84    0.86      0.35 5.4 0.022  0.33
## Relatedness_PE_3      0.84      0.84    0.87      0.35 5.4 0.023  0.32
## Competence_PE_1       0.83      0.83    0.86      0.33 4.9 0.021  0.31
## Competence_PE_2       0.83      0.83    0.86      0.33 5.0 0.021  0.32
## Competence_PE_3       0.83      0.83    0.87      0.33 5.0 0.025  0.31
## Competence_PE_4       0.84      0.84    0.87      0.34 5.2 0.022  0.32
##
##  Item statistics
##                     r r.cor r.drop
## Autonomy_PE_1    0.50  0.42   0.38
## Autonomy_PE_2    0.61  0.57   0.50
## Autonomy_PE_3    0.65  0.60   0.55
## Autonomy_PE_4    0.72  0.70   0.64
## Relatedness_PE_1 0.53  0.46   0.42
## Relatedness_PE_2 0.59  0.56   0.49
## Relatedness_PE_3 0.59  0.55   0.49
## Competence_PE_1  0.74  0.73   0.66
## Competence_PE_2  0.71  0.68   0.62
## Competence_PE_3  0.70  0.67   0.62
## Competence_PE_4  0.66  0.63   0.56
```

```
## Between
psych::alpha(ab_mat, check.keys = TRUE)
```

```
## Warning in psych::alpha(ab_mat, check.keys = TRUE): Some items were negatively correlated
with total scale and were automatically reversed.
##  This is indicated by a negative sign for the variable name.
```

```
##
## Reliability analysis
## Call: psych::alpha(x = ab_mat, check.keys = TRUE)
##
##   raw_alpha std.alpha G6(smc) average_r S/N median_r
##       0.84      0.83       1      0.31 4.9     0.27
##
##  Reliability if an item is dropped:
##                   raw_alpha std.alpha G6(smc) average_r S/N var.r med.r
## Autonomy_PE_1          0.82      0.81       1      0.30 4.2  0.14  0.26
## Autonomy_PE_2          0.77      0.78       1      0.26 3.6  0.15  0.23
## Autonomy_PE_3          0.77      0.78       1      0.26 3.5  0.15  0.23
## Autonomy_PE_4          0.78      0.80       1      0.28 3.9  0.15  0.26
## Relatedness_PE_1       0.84      0.84       1      0.34 5.2  0.14  0.35
## Relatedness_PE_2       0.85      0.85       1      0.36 5.6  0.13  0.35
## Relatedness_PE_3-      0.83      0.79       1      0.27 3.7  0.17  0.22
## Competence_PE_1        0.84      0.84       1      0.34 5.2  0.17  0.38
## Competence_PE_2        0.83      0.84       1      0.35 5.4  0.13  0.31
## Competence_PE_3        0.82      0.80       1      0.29 4.1  0.15  0.26
## Competence_PE_4        0.85      0.85       1      0.36 5.7  0.16  0.45
##
##  Item statistics
##                      r r.cor r.drop
## Autonomy_PE_1     0.70  0.70  0.724
## Autonomy_PE_2     0.92  0.92  0.973
## Autonomy_PE_3     0.96  0.96  0.988
## Autonomy_PE_4     0.80  0.80  0.860
## Relatedness_PE_1  0.40  0.40  0.226
## Relatedness_PE_2  0.29  0.29 -0.011
## Relatedness_PE_3- 0.90  0.90  0.739
## Competence_PE_1   0.41  0.41  0.238
## Competence_PE_2   0.35  0.35  0.441
## Competence_PE_3   0.75  0.75  0.570
## Competence_PE_4   0.26  0.26  0.075
```

Autonomy subscale

```
## Within
psych::alpha(pw_mat[c(1:4), c(1:4)])
```

```
##
## Reliability analysis
## Call: psych::alpha(x = pw_mat[c(1:4), c(1:4)])
##
##   raw_alpha std.alpha G6(smc) average_r S/N median_r
##       0.78      0.78    0.75      0.47 3.6     0.46
##
##  Reliability if an item is dropped:
##                 raw_alpha std.alpha G6(smc) average_r S/N  var.r med.r
## Autonomy_PE_1      0.77      0.77    0.71      0.53 3.4 0.0146  0.55
## Autonomy_PE_2      0.70      0.71    0.63      0.45 2.4 0.0121  0.45
## Autonomy_PE_3      0.76      0.76    0.69      0.52 3.2 0.0109  0.46
## Autonomy_PE_4      0.66      0.66    0.57      0.40 2.0 0.0041  0.40
##
##  Item statistics
##                  r r.cor r.drop
## Autonomy_PE_1 0.72  0.56   0.50
## Autonomy_PE_2 0.80  0.72   0.62
## Autonomy_PE_3 0.73  0.59   0.52
## Autonomy_PE_4 0.85  0.81   0.71
```

```
## Between
psych::alpha(ab_mat[c(1:4), c(1:4)])
```

```
##
## Reliability analysis
## Call: psych::alpha(x = ab_mat[c(1:4), c(1:4)])
##
##   raw_alpha std.alpha G6(smc) average_r S/N median_r
##       0.95      0.97    0.97      0.88  29     0.87
##
##  Reliability if an item is dropped:
##                 raw_alpha std.alpha G6(smc) average_r S/N  var.r med.r
## Autonomy_PE_1      0.97      0.98    0.99      0.94  49 0.0018  0.95
## Autonomy_PE_2      0.92      0.94    0.92      0.84  15 0.0042  0.85
## Autonomy_PE_3      0.91      0.94    0.94      0.85  17 0.0089  0.82
## Autonomy_PE_4      0.92      0.96    0.96      0.88  23 0.0071  0.85
##
##  Item statistics
##                  r r.cor r.drop
## Autonomy_PE_1 0.90  0.84   0.83
## Autonomy_PE_2 0.98  0.99   0.96
## Autonomy_PE_3 0.98  0.98   0.95
## Autonomy_PE_4 0.95  0.94   0.89
```

Relatedness subscale

```
## Within
psych::alpha(pw_mat[c(5:7), c(5:7)], check.keys = T)
```

```
##
## Reliability analysis
## Call: psych::alpha(x = pw_mat[c(5:7), c(5:7)], check.keys = T)
##
##   raw_alpha std.alpha G6(smc) average_r S/N median_r
##       0.79      0.79    0.74      0.55 3.7     0.53
##
##  Reliability if an item is dropped:
##                  raw_alpha std.alpha G6(smc) average_r S/N var.r med.r
## Relatedness_PE_1      0.82      0.82    0.70      0.70 4.6    NA  0.70
## Relatedness_PE_2      0.59      0.59    0.42      0.42 1.4    NA  0.42
## Relatedness_PE_3      0.69      0.69    0.53      0.53 2.3    NA  0.53
##
##  Item statistics
##                     r r.cor r.drop
## Relatedness_PE_1 0.78  0.58   0.52
## Relatedness_PE_2 0.89  0.83   0.73
## Relatedness_PE_3 0.84  0.75   0.64
```

```
## Between
psych::alpha(ab_mat[c(5:7), c(5:7)], check.keys = T)
```

```
## Warning in psych::alpha(ab_mat[c(5:7), c(5:7)], check.keys = T): Some items were negative
ly correlated with total scale and were automatically reversed.
##  This is indicated by a negative sign for the variable name.
```

```
##
## Reliability analysis
## Call: psych::alpha(x = ab_mat[c(5:7), c(5:7)], check.keys = T)
##
##   raw_alpha std.alpha G6(smc) average_r S/N median_r
##       0.65      0.75    0.69       0.5   3     0.48
##
##  Reliability if an item is dropped:
##                   raw_alpha std.alpha G6(smc) average_r S/N var.r med.r
## Relatedness_PE_1       0.60      0.65    0.48      0.48 1.9    NA  0.48
## Relatedness_PE_2       0.35      0.55    0.38      0.38 1.2    NA  0.38
## Relatedness_PE_3-      0.68      0.79    0.65      0.65 3.7    NA  0.65
##
##  Item statistics
##                      r r.cor r.drop
## Relatedness_PE_1  0.83  0.71   0.63
## Relatedness_PE_2  0.87  0.79   0.69
## Relatedness_PE_3- 0.76  0.54   0.45
```

Competence subscale

```
## Within
psych::alpha(pw_mat[c(8:11), c(8:11)], check.keys = T)
```

```
##
## Reliability analysis
## Call: psych::alpha(x = pw_mat[c(8:11), c(8:11)], check.keys = T)
##
##   raw_alpha std.alpha G6(smc) average_r S/N median_r
##       0.85      0.85    0.82      0.59 5.8     0.58
##
##  Reliability if an item is dropped:
##                 raw_alpha std.alpha G6(smc) average_r S/N  var.r med.r
## Competence_PE_1      0.79      0.79    0.72      0.56 3.8 0.0022  0.53
## Competence_PE_2      0.80      0.80    0.74      0.58 4.1 0.0049  0.54
## Competence_PE_3      0.85      0.85    0.79      0.65 5.6 0.0016  0.66
## Competence_PE_4      0.81      0.81    0.75      0.59 4.3 0.0082  0.54
##
##  Item statistics
##                    r r.cor r.drop
## Competence_PE_1 0.87  0.82   0.75
## Competence_PE_2 0.85  0.78   0.72
## Competence_PE_3 0.78  0.65   0.61
## Competence_PE_4 0.84  0.76   0.70
```

```
## Between
psych::alpha(ab_mat[c(8:11), c(8:11)], check.keys = T)
```

```
## Warning in psych::alpha(ab_mat[c(8:11), c(8:11)], check.keys = T): Some items were negati
vely correlated with total scale and were automatically reversed.
##  This is indicated by a negative sign for the variable name.
```

```
##
## Reliability analysis
## Call: psych::alpha(x = ab_mat[c(8:11), c(8:11)], check.keys = T)
##
##   raw_alpha std.alpha G6(smc) average_r  S/N median_r
##       0.18      0.22    0.22     0.065 0.28    0.019
##
##  Reliability if an item is dropped:
##                  raw_alpha std.alpha G6(smc) average_r   S/N   var.r
## Competence_PE_1       0.21      0.24    0.20     0.094  0.31 0.02116
## Competence_PE_2      -0.13     -0.16   -0.10    -0.048 -0.14 0.00041
## Competence_PE_3-      0.18      0.19    0.16     0.071  0.23 0.01982
## Competence_PE_4       0.27      0.33    0.28     0.142  0.50 0.02357
##                   med.r
## Competence_PE_1   0.072
## Competence_PE_2  -0.039
## Competence_PE_3-  0.072
## Competence_PE_4   0.210
##
##  Item statistics
##                     r  r.cor r.drop
## Competence_PE_1  0.51  0.162  0.027
## Competence_PE_2  0.70  0.636  0.335
## Competence_PE_3- 0.54  0.246  0.086
## Competence_PE_4  0.44 -0.019 -0.030
```

Criterion validity
